# Excavating Precursors from Herb Pairs *Polygala tenuifolia* and *Acori tatarinowii*: Synthesis and Anticonvulsant Activity Evaluation of 3,4,5-Trimethoxycinnamic Acid (TMCA) Piperazine Amide Derivatives

**DOI:** 10.3390/ph18091312

**Published:** 2025-09-01

**Authors:** Zefeng Zhao, Mengchen Lei, Yongqi Wang, Yujun Bai, Haifa Qiao

**Affiliations:** 1 Institute for Chinese Medicine Frontier Interdisciplinary Science and Technology, Shaanxi University of Chinese Medicine, Xianyang 712046, China; 2 Biomedicine Key Laboratory of Shaanxi Province, Northwest University, 229 Taibai Road, Xi’an 710069, China

**Keywords:** synthesis, 3,4,5-trimethoxycinnamic acid, piperazine amide derivatives, anticonvulsant, docking

## Abstract

**Background**: Epilepsy is a cluster of central nervous system (CNS) disorders identified by recurrent seizures, which affects about 60 million people around the world. In this research, a total of 40 types of 3,4,5-trimethoxycinnamic acid (TMCA) piperazine amide derivatives were designed and synthesized, inspired by the traditional Chinese medicine (TCM) herb pair drugs *Polygala tenuifolia* and *Acori tatarinowii*, followed by determination of their anticonvulsant potency. **Methods**: All the TMCA analogues were tested for their anticonvulsant potential through two acute models of seizures induced in mice: the maximal electroshock (MES) and sc-pentylenetetrazole (PTZ) models. In addition, the lactate dehydrogenase (LDH) inhibitory activity was determined in vitro. **Results**: The results showed that compounds **A3**, **A9**, **A12**, **A14**, **B9**, and **B12** exhibited preferable anticonvulsant activity in the primary evaluation. In addition, the molecular docking results predicted good interactions of screened analogues with the LDH. Molecular dynamic simulation was used to reveal the consensual binding affinity between the most promising compound (**B9**) and active site interactions with LDH. Electroencephalogram (EEG) analysis and silver and immunofluorescence staining were performed to illustrate the anti-epilepsy potential of compound **B9**. **Conclusions**: Novel derivatives in this study provide new cores for the further design and optimization inspired by TCM herb pair drugs *P. tenuifolia* and *A. tatarinowii*, with the aim to explore new anticonvulsant agents.

## 1. Introduction

**Figure 1:**
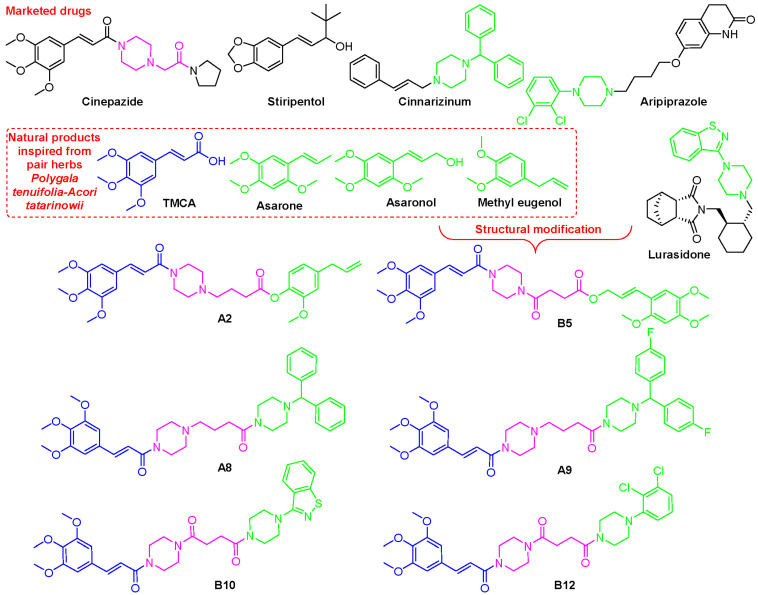
Design of TMCA piperazine amide derivatives as anticonvulsant agents inspired by TCM herb pairs *P. tenuifolia* and *A. tatarinowii*.

Epilepsy is a severe chronic neurological disorder affecting 0.5–1.0% of the global population [1,2]. Anti-epileptic drugs (AEDs) remain the primary therapeutic approach. Natural products offer promising avenues for novel interventions in this field [3,4]. Traditional Chinese medicine (TCM), recognized as a valuable natural resource, demonstrates favorable safety and efficacy profiles while mitigating treatment-associated side effects [5]. Building upon prior research [6,7,8], we identified 3,4,5-trimethoxycinnamic acid (TMCA) from *P. tenuifolia* as a precursor exhibiting significant anticonvulsant potential, providing a rationale for exploring TMCA derivatives (Figure 1).

**Scheme 1:**
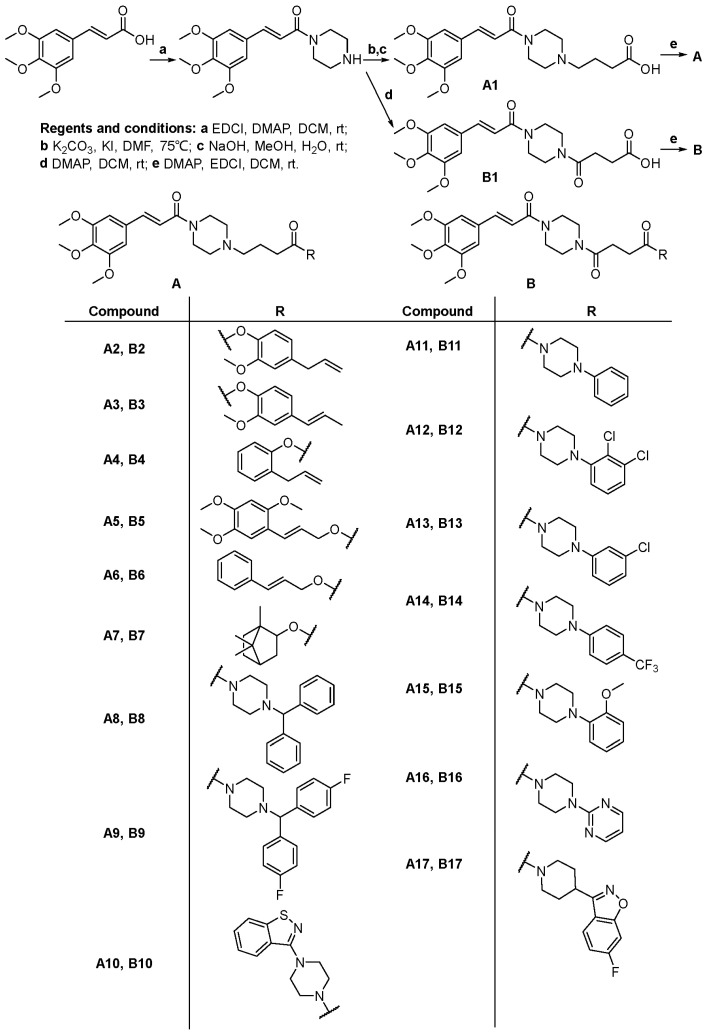
Synthesis of compounds **A1–17** and **B1–17**.

In this study, we synthesized a series of TMCA piperazine amide derivatives (Scheme 1, **A1–17**, **B1–17**). The synthetic derivatives were classified into two categories according to the linker of butyryl moiety (**A1–17**) and butanedioic amide (**B1–17**), and the substitutions mainly constituted five kinds of *A. tatarinowii* component derivatives (substitutions **S2–6**) and nine kinds of arylpiperazine derivatives (**S8–16**). TMCA is an established central nervous system (CNS) agent targeting the GABA_A_/BZ receptor [9], 5-HT (5-hydroxytryptamine) [10], AChE (acetylcholine) [11] and EP2 [12]. Its role as the functional core in marketed drugs, such as cinepazide and cinepaxadil, underscores its potential as a novel drug precursor.

The rationale for selecting the residual groups for amide/ester bond formation comprised three elements: (1) TCM-inspired neural actives—incorporation of *A. tatarinowii* constituents/derivatives, including methyl eugenol (**S2**), isoeugenol (**S3**), and asaronol (**S5**), to explore anticonvulsant potential and leverage TCM herb-pairing principles [13,14,15]. Borneol (**S7**), a recognized CNS-active TCM compound, was also included. (2) Market-drug-derived arylpiperazines—inclusion of arylpiperazine moieties from established drugs (cinnarizine (S8), flunarizine (S9), lurasidone (**S10**), aripiprazole (**S12**), known for neural activity [16,17,18]; (3) integration of piperidine analogue tetrahydroisoquinoline (**S17**) to broaden structure–activity relationship (SAR) exploration. All synthesized derivatives were fully characterized. Their anticonvulsant potency was evaluated using classical seizure models. The article details results including inhibition of lactate dehydrogenase (LDH), neurotoxicity assessment, electroencephalogram (EEG) analysis, silver and immunofluorescence staining, molecular docking studies and SAR investigations.

## 2. Results

### 2.1. Chemistry

All titled compounds **A1–17** and **B1–17** were successfully prepared through the synthetic protocols presented in Scheme 1, and the structurally simple intermediates **A1** and **B1** can be characterized by the subsequent derivatives. The spectra are listed in the Supplementary Materials.

*4-allyl-2-methoxyphenyl (E)-4-(4-(3-(3,4,5-trimethoxyphenyl)acryloyl)piperazin-1-yl)butanoate* (***A2***): yellow liquid; yield: 80%; R_f_ = 0.7 (DCM/Me = 10:1, *v*/*v*); ^1^H NMR (400 MHz, CDCl_3_) δ 7.78 (d, *J* = 15.8 Hz, 1H), 7.03 (d, *J* = 7.9 Hz, 1H), 6.87–6.76 (m, 4H), 6.75–6.66 (m, 1H), 6.58 (d, *J* = 15.9 Hz, 1H), 6.05–5.89 (m, 1H), 5.17–5.01 (m, 3H), 3.90 (d, *J* = 1.1 Hz, 9H), 3.87 (d, *J* = 2.1 Hz, 2H), 3.82 (d, *J* = 10.2 Hz, 3H), 3.36 (dd, *J* = 32.9, 6.8 Hz, 3H), 2.68–2.34 (m, 2H), 1.41 (s, 1H), 1.27 (d, *J* = 13.6 Hz, 1H); ^13^C NMR (101 MHz, CDCl_3_) δ 165.47, 165.25, 153.57, 151.10, 146.54, 140.47, 139.14, 138.08, 137.18, 129.90, 122.77, 120.84, 116.29, 115.63, 112.88, 105.56, 105.13, 61.12, 56.34, 56.29, 56.01, 55.97, 55.92, 40.25, 31.75, 29.77; HRMS (ESI^+^) calcd for C_30_H_38_N_2_O_7_ (*m*/*z* [M + H]^+^): 539.2757; found: 539.2725.

*methoxy-4-((E)-prop-1-en-1-yl)phenyl 4-(4-((E)-3-(3,4,5-trimethoxyphenyl)acryloyl)piperazin-1-yl)butanoate* (***A3***): white liquid; yield: 73%; R_f_ = 0.65 (DCM/Me = 10:1, *v*/*v*);^1^H NMR (400 MHz, CDCl_3_) δ 6.95 (d, *J* = 4.0 Hz, 1H), 6.77–6.71 (m, 1H), 6.64–6.55 (m, 2H), 5.91–5.83 (m, 1H), 5.05–4.93 (m, 2H), 3.78 (s, 3H), 3.65 (s, 9H), 3.34 (s, 14H);^13^C NMR (101 MHz, CDCl_3_) δ 171.59, 165.26, 153.39, 153.35, 150.95, 146.48, 143.87, 139.00, 137.85, 137.08, 131.84, 122.63, 121.12, 120.68, 115.46, 111.13, 105.39, 105.04, 60.92, 60.89, 56.11, 56.06, 55.77, 55.75, 40.08, 39.85, 39.62; HRMS (ESI^+^) calcd for C_30_H_38_N_2_O_7_ (*m*/*z* [M + H]^+^): 539.2757; found: 539.2757.

*2-allylphenyl (E)-4-(4-(3-(3,4,5-trimethoxyphenyl)acryloyl)piperazin-1-yl)butanoate* (***A4***): yellow solid; yield: 75%; R_f_ = 0.8 (DCM/Me = 10:1, *v*/*v*); ^1^H NMR (400 MHz, CDCl_3_) δ 7.28 (s, 1H), 7.08 (s, 1H), 6.82 (s, 2H), 6.05–5.87 (m, 2H), 5.09–5.06 (m, 2H), 5.05–5.02 (m, 3H), 3.89 (d, *J* = 3.8 Hz, 9H), 3.84 (s, 1H), 3.39 (s, 1H), 3.37 (s, 8H), 3.09 (s, 6H);^13^C NMR (101 MHz, CDCl_3_) δ 154.20, 139.68, 136.51, 130.57, 128.04, 125.38, 121.08, 116.64, 115.93, 35.26, 29.78; HRMS (ESI^+^) calcd for C_29_H_36_N_2_O_6_ (*m*/*z* [M + H]^+^): 509.2652; found: 509.2621.

*(E)-3-(2,4,5-trimethoxyphenyl)allyl 4-(4-((E)-3-(3,4,5-trimethoxyphenyl)acryloyl)piperazin-1-yl)butanoate* (***A5***): yellow solid; yield: 64%; R_f_ = 0.9 (DCM/Me = 10:1, *v*/*v*); ^1^H NMR (400 MHz, CDCl_3_) δ 7.62 (d, *J* = 15.9 Hz, 1H), 6.97 (t, *J* = 8.0 Hz, 2H), 6.73 (s, 2H), 6.48 (s, 1H), 6.37 (d, *J* = 15.9 Hz, 1H), 6.28–6.20 (m, 1H), 4.86–4.83 (m, 2H), 3.87 (s, 3H), 3.85 (s, 9H), 3.84 (s, 2H), 3.81 (s, 3H), 2.88 (d, *J* = 27.8 Hz, 1H), 1.93 (d, *J* = 68.8 Hz, 1H), 1.30–1.16 (m, 7H), 0.94–0.79 (m, 3H); ^13^C NMR (101 MHz, CDCl_3_) δ 166.83, 162.59, 153.47, 151.78, 150.04, 144.93, 143.36, 140.15, 129.98, 129.28, 121.37, 117.39, 116.94, 110.10, 105.27, 97.56, 66.00, 60.99, 56.61, 56.56, 56.17, 56.10, 31.46, 29.74, 22.73; HRMS (ESI^+^) calcd for C_33_H_42_N_2_O_9_ (*m*/*z* [M + H]^+^): 599.2969; found: 599.1808.

*cinnamyl 4-(4-((E)-3-(3,4,5-trimethoxyphenyl)acryloyl)piperazin-1-yl)butanoate* (***A6***): yellow solid; yield: 68%; R_f_ = 0.8 (DCM/Me = 10:1, *v*/*v*); ^1^H NMR (400 MHz, CDCl_3_) δ 7.64 (d, *J* = 15.9 Hz, 1H), 7.41–7.32 (m, 7H), 6.75 (s, 2H), 6.60 (dt, *J* = 15.9, 1.6 Hz, 2H), 4.87 (dd, *J* = 6.5, 1.3 Hz, 2H), 3.87 (s, 9H), 3.70 (q, *J* = 7.0 Hz, 2H), 2.93 (s, 1H), 2.87 (s, 1H), 1.89 (s, 6H), 1.23 (t, *J* = 7.0 Hz, 4H); ^13^C NMR (101 MHz, CDCl_3_) δ 173.40, 162.74, 153.51, 153.48, 140.23, 136.81, 136.30, 134.43, 129.96, 128.71, 128.67, 128.20, 127.74, 123.30, 117.21, 105.36, 105.11, 65.27, 61.06, 58.48, 57.52, 56.30, 56.24, 31.56, 22.04; HRMS (ESI^+^) calcd for C_29_H_36_N_2_O_6_ (*m*/*z* [M + H]^+^): 509.2652; found: 509.2553.

*rel-(1R,2S,4R)-1,7,7-trimethylbicyclo [2.2.1]heptan-2-yl 4-(4-((E)-3-(3,4,5-trimethoxyphenyl)acryloyl)piperazin-1-yl)butanoate* (***A7***): yellow solid; yield: 75%; R_f_ = 0.9 (DCM/Me = 10:1, *v*/*v*); ^1^H NMR (400 MHz, CDCl_3_) δ 8.54 (d, *J* = 8.5 Hz, 1H), 8.06–7.96 (m, 2H), 7.58 (d, *J* = 15.5 Hz, 2H), 3.93 (d, *J* = 11.4 Hz, 9H), 2.31–2.26 (m, 1H), 2.26 (s, 1H), 2.11 (s, 5H), 1.92–1.88 (m, 1H), 1.88–1.84 (m, 2H), 1.74–1.68 (m, 3H), 1.61 (t, *J* = 4.6 Hz, 2H), 1.29–1.17 (m, 4H), 1.01 (s, 2H), 0.89 (s, 2H), 0.86 (s, 4H), 0.81 (s, 3H); ^13^C NMR (101 MHz, CDCl_3_) δ 161.86, 153.70, 153.56, 149.52, 141.58, 133.11, 129.40, 127.07, 106.40, 105.35, 80.06, 61.19, 61.10, 56.50, 56.29, 51.86, 49.62, 49.09, 48.15, 45.21, 45.15, 40.50, 39.13, 34.04, 28.40, 26.04, 20.60, 20.31, 20.23, 18.80; HRMS (ESI^+^) calcd for C_30_H_44_N_2_O_6_ (*m*/*z* [M + H]^+^): 529.3278; found: 529.3278.

*(E)-1-(4-benzhydrylpiperazin-1-yl)-4-(4-(3-(3,4,5-trimethoxyphenyl)acryloyl)piperazin-1-yl)butan-1-one* (***A8***): white solid; yield: 78%; R_f_ = 0.9 (DCM/Me = 10:1, *v*/*v*); ^1^H NMR (400 MHz, CDCl_3_) δ 7.56 (d, *J* = 15.3 Hz, 1H), 7.45–7.37 (m, 4H), 7.32–7.23 (m, 5H), 7.19 (t, *J* = 7.3 Hz, 2H), 6.69 (s, 2H), 4.26 (s, 1H), 3.86 (d, *J* = 2.3 Hz, 9H), 3.79–3.55 (m, 5H), 2.50–2.29 (m, 5H), 1.91–1.61 (m, 2H), 1.41 (d, *J* = 5.6 Hz, 1H), 1.26 (d, *J* = 12.8 Hz, 2H); ^13^C NMR (101 MHz, CDCl_3_) δ 165.38, 153.54, 153.51, 142.83, 142.23, 139.66, 131.00, 128.75, 128.73, 128.01, 127.31, 116.45, 105.12, 105.03, 76.08, 61.08, 56.34, 52.32, 51.74, 46.19, 42.50, 31.77, 29.77; HRMS (ESI^+^) calcd for C_37_H_46_N_4_O_5_ (*m*/*z* [M + H]^+^): 627.3546; found: 627.3509.

*(E)-1-(4-(bis(4-fluorophenyl)methyl)piperazin-1-yl)-4-(4-(3-(3,4,5-trimethoxyphenyl)acryloyl)piperazin-1-yl)butan-1-one* (***A9***): white solid; yield: 69%; R_f_ = 0.9 (DCM/Me = 10:1, *v*/*v*);^1^H NMR (400 MHz, CDCl_3_) δ 7.60–7.54 (m, 1H), 7.35 (s, 5H), 7.01 (s, 2H), 6.97 (s, 2H), 6.72 (d, *J* = 13.6 Hz, 3H), 3.87 (q, *J* = 2.1 Hz, 9H), 3.73 (s, 2H), 3.66 (s, 3H), 2.41 (s, 4H), 2.03 (d, *J* = 17.2 Hz, 1H), 1.59 (t, *J* = 1.5 Hz, 11H); ^13^C NMR (101 MHz, CDCl_3_) δ 165.39, 153.52, 143.04, 139.64, 137.74, 130.93, 129.39, 129.31, 122.41, 116.24, 115.85, 115.64, 104.97, 92.30, 74.43, 61.12, 56.29, 51.65, 34.48, 28.81; HRMS (ESI^+^) calcd for C_37_H_44_F_2_N_4_O_5_ (*m*/*z* [M]^+^): 662.3280; found: 662.3478.

*(E)-1-(4-(benzo[d]isothiazol-3-yl)piperazin-1-yl)-4-(4-(3-(3,4,5-trimethoxyphenyl)acryloyl)piperazin-1-yl)butan-1-one* (***A10***): white solid; yield: 74%; R_f_ = 0.9 (DCM/Me = 10:1, *v*/*v*); ^1^H NMR (400 MHz, CDCl_3_) δ 7.90 (d, *J* = 8.0 Hz, 1H), 7.81 (d, *J* = 8.2 Hz, 2H), 7.62 (d, *J* = 15.3 Hz, 1H), 7.47 (t, *J* = 7.5 Hz, 2H), 7.37 (t, *J* = 7.6 Hz, 2H), 3.88 (d, *J* = 5.3 Hz, 9H), 3.86 (s, 4H), 3.85 (s, 1H), 3.69 (pd, *J* = 7.8, 4.3 Hz, 1H), 3.57 (t, *J* = 5.1 Hz, 5H), 3.50 (dd, *J* = 7.8, 4.3 Hz, 1H), 2.45 (dd, *J* = 17.4, 10.2 Hz, 2H), 1.99–1.47 (m, 1H), 1.43–1.19 (m, 3H); ^13^C NMR (101 MHz, CDCl_3_) δ 171.42, 165.72, 163.36, 153.44, 153.40, 152.83, 143.37, 139.63, 130.77, 127.84, 127.82, 124.23, 123.64, 123.41, 120.73, 105.02, 104.94, 61.02, 61.00, 57.67, 56.24, 56.21, 53.43, 50.39, 50.07, 49.89, 45.63, 45.29, 42.04, 30.76, 22.17; HRMS (ESI^+^) calcd for C_31_H_39_N_5_O_5_S (*m*/*z* [M + H]^+^): 594.2750; found: 594.2787.

*(E)-1-(4-phenylpiperazin-1-yl)-4-(4-(3-(3,4,5-trimethoxyphenyl)acryloyl)piperazin-1-yl)butan-1-one* (***A11***): yellow solid; yield: 75%; R_f_ = 0.6 (DCM/Me = 10:1, *v*/*v*); ^1^H NMR (400 MHz, CDCl_3_) δ 7.62 (d, *J* = 15.4 Hz, 1H), 7.24 (s, 1H), 6.95 (s, 1H), 6.94 (s, 6H), 3.90 (s, 6H), 3.88 (s, 3H), 3.24 (s, 2H), 3.15 (s, 5H), 3.14 (s, 2H), 3.03 (d, *J* = 4.5 Hz, 1H), 3.00 (s, 5H), 2.23 (s, 7H); ^13^C NMR (101 MHz, CDCl_3_) δ 165.58, 154.53, 153.55, 149.23, 143.32, 139.81, 130.88, 129.40, 129.19, 120.70, 119.94, 116.76, 116.29, 106.69, 105.18, 61.09, 57.79, 56.35, 51.67, 50.43, 49.27, 46.16, 31.77, 22.31; HRMS (ESI^+^) calcd for C_30_H_40_N_4_O_5_ (*m*/*z* [M + H]^+^): 537.3077; found: 537.3027.

*(E)-1-(4-(2,3-dichlorophenyl)piperazin-1-yl)-4-(4-(3-(3,4,5-trimethoxyphenyl)acryloyl)piperazin-1-yl)butan-1-one* (***A12***): white solid; yield: 77%; R_f_ = 0.9 (PE/EA = 2:1, *v*/*v*); ^1^H NMR (400 MHz, CDCl_3_) δ 7.63 (d, *J* = 15.3 Hz, 1H), 7.20–7.18 (m, 1H), 6.93 (dd, *J* = 7.7, 1.8 Hz, 1H), 6.80 (d, *J* = 15.3 Hz, 1H), 6.75 (s, 3H), 3.89 (d, *J* = 3.0 Hz, 9H), 3.87 (d, *J* = 2.4 Hz, 11H), 3.07 (t, *J* = 4.9 Hz, 5H), 2.50 (s, 1H), 1.26 (d, *J* = 13.8 Hz, 5H); ^13^C NMR (101 MHz, CDCl_3_) δ 171.41, 165.69, 153.53, 153.50, 150.65, 143.40, 139.72, 134.33, 130.85, 127.84, 127.72, 125.37, 118.89, 116.14, 105.07, 105.02, 61.11, 57.79, 56.32, 51.99, 51.24, 46.26, 45.88, 42.53, 41.93, 32.04, 29.82; HRMS (ESI^+^) calcd for C_30_H_38_Cl_2_N_4_O_5_ (*m*/*z* [M + H]^+^): 605.2298; found: 605.2329.

*(E)-1-(4-(3-chlorophenyl)piperazin-1-yl)-4-(4-(3-(3,4,5-trimethoxyphenyl)acryloyl)piperazin-1-yl)butan-1-one* (***A13***): yellow solid; yield: 73%; R_f_ = 0.8 (DCM/Me = 10:1, *v*/*v*);^1^H NMR (400 MHz, CDCl_3_) δ 7.62 (d, *J* = 15.3 Hz, 1H), 7.21–7.15 (m, 1H), 6.88 (t, *J* = 2.1 Hz, 1H), 6.85 (ddd, *J* = 7.8, 1.9, 0.9 Hz, 1H), 6.81–6.78 (m, 1H), 6.75 (s, 1H), 6.75 (s, 2H), 3.89 (s, 6H), 3.87 (s, 3H), 3.84 (d, *J* = 15.3 Hz, 6H), 3.81 (s, 3H), 3.25 (s, 1H), 3.23 (s, 3H), 3.23 (s, 4H), 3.22 (s, 1H), 2.55–2.36 (m, 1H), 1.77 (s, 2H), 1.43–1.21 (m, 1H); ^13^C NMR (101 MHz, CDCl_3_) δ 171.32, 165.62, 153.56, 153.53, 152.01, 143.51, 139.89, 135.19, 130.80, 130.32, 120.27, 116.44, 116.02, 114.51, 105.23, 105.14, 61.09, 57.76, 56.35, 56.34, 49.31, 45.64, 45.35, 31.75, 23.15; HRMS (ESI^+^) calcd for C_30_H_39_ClN_4_O_5_ (*m*/*z* [M + H]^+^): 571.2687; found: 571.2643.

*(E)-1-(4-(4-(trifluoromethyl)phenyl)piperazin-1-yl)-4-(4-(3-(3,4,5-trimethoxyphenyl)acryloyl)piperazin-1-yl)butan-1-one* (***A14***): yellow solid; yield: 67%; R_f_ = 0.8 (DCM/Me = 10:1, *v*/*v*);^1^H NMR (400 MHz, CDCl_3_) δ 7.64 (d, *J* = 15.3 Hz, 1H), 7.52 (s, 1H), 7.50 (s, 2H), 6.95 (s, 1H), 6.75 (s, 3H), 3.90 (s, 8H), 3.88 (s, 1H), 3.36 (s, 2H), 3.35 (s, 4H), 3.34 (s, 1H), 3.31–3.24 (m, 1H), 2.61–2.37 (m, 1H), 1.68 (s, 8H), 1.41 (s, 1H), 1.25 (s, 4H); ^13^C NMR (101 MHz, CDCl_3_) δ 165.64, 153.57, 143.68, 139.86, 130.77, 126.68, 115.87, 115.12, 105.16, 61.14, 56.35, 48.23, 41.85, 29.83; HRMS (ESI^+^) calcd for C_29_H_35_F_3_N_4_O_5_ (*m*/*z* [M + H]^+^): 577.2638; found: 577.2661.

*(E)-1-(4-(2-methoxyphenyl)piperazin-1-yl)-4-(4-(3-(3,4,5-trimethoxyphenyl)acryloyl)piperazin-1-yl)butan-1-one* (***A15***): yellow solid; yield: 75%; R_f_ = 0.8 (DCM/Me = 10:1, *v*/*v*); ^1^H NMR (400 MHz, CDCl_3_) δ 7.61 (d, *J* = 15.2 Hz, 1H), 7.05–7.03 (m, 1H), 6.94–6.88 (m, 3H), 6.81 (d, *J* = 15.3 Hz, 1H), 6.74 (d, *J* = 6.9 Hz, 2H), 3.96–3.90 (m, 2H), 3.89 (s, 6H), 3.88–3.87 (m, 12H), 3.87 (s, 3H), 3.84–3.62 (m, 1H), 3.50–3.31 (m, 1H), 3.21–2.99 (m, 5H), 2.57–2.39 (m, 2H), 1.45–1.21 (m, 2H);^13^C NMR (101 MHz, CDCl_3_) δ 171.21, 165.55, 165.49, 153.49, 152.33, 143.10, 140.66, 139.63, 130.95, 123.73, 121.16, 118.55, 116.35, 115.94, 111.38, 105.04, 61.08, 57.77, 56.29, 55.59, 55.54, 51.26, 51.12, 50.69, 47.88, 46.29, 45.91, 44.10, 42.53, 31.74, 22.18; HRMS (ESI^+^) calcd for C_31_H_42_N_4_O_6_ (*m*/*z* [M + H]^+^): 567.3183; found: 567.3197.

*(E)-1-(4-(pyrimidin-2-yl)piperazin-1-yl)-4-(4-(3-(3,4,5-trimethoxyphenyl)acryloyl)piperazin-1-yl)butan-1-one* (***A16***): white solid; yield: 71%; R_f_ = 0.9 (DCM/Me = 10:1, *v*/*v*);^1^H NMR (400 MHz, CDCl_3_) δ 8.25 (s, 1H), 8.24 (s, 1H), 7.53 (d, *J* = 15.3 Hz, 1H), 6.75 (d, *J* = 15.3 Hz, 1H), 6.69 (s, 2H), 6.47 (t, *J* = 4.8 Hz, 1H), 3.82 (s, 6H), 3.78 (s, 3H), 3.71 (t, *J* = 9.1 Hz, 6H), 3.62 (d, *J* = 7.0 Hz, 2H), 3.58 (d, *J* = 7.0 Hz, 2H), 3.35 (s, 1H), 2.47 (s, 11H); ^13^C NMR (101 MHz, CDCl_3_) δ 165.68, 162.55, 161.40, 157.76, 153.34, 143.17, 139.57, 130.72, 116.15, 110.45, 105.05, 60.87, 57.97, 56.17, 50.33, 45.61, 43.55, 41.95, 36.45, 31.35; HRMS (ESI^+^) calcd for C_28_H_38_N_6_O_5_ (*m*/*z* [M + H]^+^): 539.2982; found: 539.2891.

(*E)-1-(4-(6-fluorobenzo[d]isoxazol-3-yl)piperidin-1-yl)-4-(4-(3-(3,4,5-trimethoxyphenyl)acryloyl)piperazin-1-yl)butan-1-one* (***A17***): white solid; yield: 76%; R_f_ = 0.9 (DCM/Me = 10:1, *v*/*v*); ^1^H NMR (400 MHz, CDCl_3_) δ 7.63 (s, 1H), 7.59 (s, 1H), 7.27 (d, J = 2.2 Hz, 1H), 7.08 (d, J = 2.2 Hz, 1H), 6.75 (s, 4H), 3.90 (s, 11H), 3.87 (s, 5H), 2.21 (d, J = 3.6 Hz, 1H), 2.18 (s, 2H), 2.03 (s, 5H), 1.67 (s, 6H); ^13^C NMR (101 MHz, CDCl_3_) δ 165.69, 164.16, 163.10, 160.30, 153.57, 143.23, 139.80, 130.95, 122.38, 117.19, 116.50, 112.93, 112.68, 105.18, 97.89, 61.11, 56.37, 45.99, 42.23, 34.51, 30.48; HRMS (ESI^+^) calcd for C_32_H_39_FN_4_O_6_ (*m*/*z* [M + H]^+^): 595.2932; found: 595.2935.

*4-allyl-2-methoxyphenyl (E)-4-oxo-4-(4-(3-(3,4,5-trimethoxyphenyl)acryloyl)piperazin-1-yl)butanoate* (***B2***): yellow liquid; yield: 77%; R_f_ = 0.9 (DCM/Me = 10:1, *v*/*v*); ^1^H NMR (400 MHz, CDCl_3_) δ 7.62 (d, *J* = 15.3 Hz, 1H), 6.98 (d, *J* = 7.9 Hz, 1H), 6.80–6.64 (m, 5H), 5.95 (ddt, *J* = 16.9, 10.1, 6.7 Hz, 1H), 5.15–5.01 (m, 2H), 3.89 (d, *J* = 9.0 Hz, 9H), 3.81 (s, 3H), 3.71 (q, *J* = 5.9 Hz, 4H), 3.60 (d, *J* = 5.3 Hz, 2H), 3.34 (dd, *J* = 20.6, 6.7 Hz, 2H), 3.05–2.93 (m, 2H), 2.77 (t, *J* = 6.8 Hz, 2H);^13^C NMR (101 MHz, CDCl_3_) δ 171.57, 165.78, 153.56, 150.90, 143.94, 139.89, 139.16, 138.01, 137.16, 130.66, 122.71, 120.85, 116.31, 115.67, 112.82, 105.16, 61.13, 56.35, 55.99, 40.22, 40.02, 31.76, 29.24, 28.05; HRMS (ESI^+^) calcd for C_30_H_36_N_2_O_8_ (*m*/*z* [M + H]^+^): 553.2550; found: 553.2551.

*2-methoxy-4-((E)-prop-1-en-1-yl)phenyl 4-oxo-4-(4-((E)-3-(3,4,5-trimethoxyphenyl)acryloyl)piperazin-1-yl)butanoate* (***B3***): yellow liquid; yield: 78%; R_f_ = 0.8 (DCM/Me = 10:1, *v*/*v*) ^1^H NMR (400 MHz, CDCl_3_) δ 7.63 (d, *J* = 15.3 Hz, 1H), 6.97 (d, *J* = 8.0 Hz, 1H), 6.84 (s, 2H), 6.82 (s, 3H), 6.77 (s, 1H), 6.75 (s, 2H), 3.87 (s, 9H), 3.79 (s, 3H), 3.69 (d, *J* = 7.1 Hz, 4H), 3.46–3.34 (m, 3H), 3.04–2.95 (m, 2H), 2.74 (t, *J* = 6.8 Hz, 2H), 2.24–2.14 (m, 2H); ^13^C NMR (101 MHz, CDCl_3_) δ 143.99, 137.95, 121.29, 115.68, 114.34, 111.18, 105.17, 56.36, 55.98, 40.03, 26.93, 14.99; HRMS (ESI^+^) calcd for C_30_H_36_N_2_O_8_ (*m*/*z* [M + H]^+^): 553.2550; found: 553.2551.

*2-allylphenyl (E)-4-oxo-4-(4-(3-(3,4,5-trimethoxyphenyl)acryloyl)piperazin-1-yl)butanoate* (***B4***): white solid; yield: 69%; R_f_ = 0.8 (DCM/Me = 10:1, *v*/*v*);^1^H NMR (400 MHz, CDCl_3_) δ 7.51 (d, *J* = 15.3 Hz, 1H), 7.14 (d, *J* = 1.4 Hz, 1H), 6.95 (s, 2H), 6.67 (s, 2H), 5.96–5.89 (m, 2H), 4.99 (s, 2H), 4.93 (s, 2H), 3.79 (d, *J* = 11.3 Hz, 9H), 3.30 (s, 3H), 3.28 (s, 4H), 2.87 (s, 4H), 2.78 (s, 2H); ^13^C NMR (101 MHz, CDCl_3_) δ 170.21, 166.02, 162.94, 154.51, 153.17, 148.66, 147.00, 144.02, 139.48, 137.71, 136.86, 135.79, 129.69, 126.91, 119.40, 114.99, 114.93, 111.55, 105.05, 60.65, 57.52, 57.15, 55.94, 55.51, 49.34, 45.30, 33.98, 31.37, 29.27; HRMS (ESI^+^) calcd for C_29_H_34_N_2_O_7_ (*m*/*z* [M + H]^+^): 523.2444; found: 523.2444.

*(E)-3-(2,4,5-trimethoxyphenyl)allyl 4-oxo-4-(4-((E)-3-(3,4,5-trimethoxyphenyl)acryloyl)piperazin-1-yl)butanoate* (***B5***): yellow solid; yield: 72%; R_f_ = 0.9 (DCM/Me = 10:1, *v*/*v*); ^1^H NMR (400 MHz, CDCl_3_) δ 7.64 (d, *J* = 15.9 Hz, 1H), 7.03–6.97 (m, 2H), 6.76 (s, 2H), 6.50 (s, 1H), 6.39 (dd, *J* = 15.9, 0.9 Hz, 1H), 6.26 (dt, *J* = 16.0, 6.7 Hz, 1H), 4.87 (d, *J* = 6.7 Hz, 2H), 3.91 (s, 3H), 3.89–3.88 (m, 9H), 3.87 (s, 3H), 3.85 (d, *J* = 0.9 Hz, 3H), 1.56 (s, 10H), 1.25 (s, 2H);^13^C NMR (101 MHz, CDCl_3_) δ 173.98, 168.46, 158.98, 153.56, 148.27, 145.04, 140.31, 129.40, 121.42, 110.06, 105.28, 97.54, 61.13, 56.72, 56.27, 47.90, 41.81, 36.81, 27.42, 21.27; HRMS (ESI^+^) calcd for C_32_H_39_N_2_NaO_10_ (*m*/*z* [M + Na]^+^): 635.2581; found: 635.2593.

*(E)-3-(2,4,5-trimethoxyphenyl)allyl 4-oxo-4-(4-((E)-3-(3,4,5-trimethoxyphenyl)acryloyl)piperazin-1-yl)butanoate* (***B6***): yellow solid; yield: 75%; R_f_ = 0.8 (DCM/Me = 10:1, *v*/*v*); ^1^H NMR (400 MHz, CDCl_3_) δ 7.58 (d, *J* = 15.3 Hz, 1H), 7.32 (s, 3H), 7.24 (s, 1H), 7.18 (s, 1H), 6.76–6.72 (m, 3H), 6.54 (s, 1H), 6.34 (s, 1H), 4.74 (s, 1H), 4.72 (s, 2H), 3.85 (d, *J* = 5.0 Hz, 9H), 3.73–3.69 (m, 3H), 3.51 (s, 2H), 3.38 (s, 2H), 2.73–2.67 (m, 2H), 2.63 (t, *J* = 5.8 Hz, 2H); ^13^C NMR (101 MHz, CDCl_3_) δ 172.73, 165.73, 162.64, 153.22, 143.66, 139.58, 136.77, 133.99, 130.14, 128.97, 128.92, 128.43, 128.35, 127.27, 122.87, 115.45, 105.05, 65.18, 60.73, 57.68, 56.02, 51.69, 49.92, 45.22, 41.96, 31.27, 29.06; HRMS (ESI^+^) calcd for C_29_H_34_N_2_O_7_ (*m*/*z* [M + H]^+^): 523.2444; found: 523.2458.

*(1R,2S,4R)-1,7,7-trimethylbicyclo [2.2.1]heptan-2-yl 4-oxo-4-(4-((E)-3-(3,4,5-trimethoxyphenyl)acryloyl)piperazin-1-yl)butanoate* (***B7***): white solid; yield: 78%; R_f_ = 0.9 (DCM/Me = 10:1, *v*/*v*); ^1^H NMR (400 MHz, CDCl_3_) δ 7.60 (d, *J* = 15.3 Hz, 1H), 6.73 (s, 1H), 6.72 (s, 3H), 6.70 (d, *J* = 1.9 Hz, 1H), 4.87 (dd, *J* = 10.1, 2.8 Hz, 1H), 3.87 (d, *J* = 1.9 Hz, 7H), 3.85 (s, 3H), 2.65 (s, 3H), 2.05 (s, 5H), 1.59 (s, 1H), 1.21 (s, 2H), 0.99 (d, *J* = 2.0 Hz, 3H), 0.87 (d, *J* = 2.1 Hz, 4H), 0.79 (s, 2H); ^13^C NMR (101 MHz, CDCl_3_) δ 173.34, 165.79, 162.72, 153.49, 143.93, 139.80, 130.59, 115.58, 105.11, 80.29, 61.06, 56.29, 49.54, 49.01, 48.86, 48.07, 47.86, 46.40, 45.11, 39.01, 33.96, 29.54, 27.30, 27.17, 19.76, 18.90, 13.42; HRMS (ESI^+^) calcd for C_30_H_42_N_2_O_7_ (*m*/*z* [M + H]^+^): 543.3070; found: 543.3071.

*(E)-1-(4-benzhydrylpiperazin-1-yl)-4-(4-(3-(3,4,5-trimethoxyphenyl)acryloyl)piperazin-1-yl)butane-1,4-dione* (***B8***): white solid; yield: 76%; R_f_ = 0.8 (DCM/Me = 10:1, *v*/*v*); ^1^H NMR (400 MHz, CDCl_3_) δ 7.59 (d, *J* = 15.3 Hz, 1H), 7.39 (d, *J* = 7.6 Hz, 5H), 7.28 (s, 2H), 7.24 (s, 1H), 7.18 (d, *J* = 7.2 Hz, 1H), 6.75–6.67 (m, 3H), 4.22 (s, 1H), 3.87 (d, *J* = 8.3 Hz, 9H), 3.69–3.64 (m, 3H), 3.59 (t, *J* = 5.1 Hz, 3H), 3.51 (t, *J* = 5.0 Hz, 2H), 2.89 (d, *J* = 28.1 Hz, 3H), 2.66 (s, 4H), 2.37 (dt, *J* = 14.6, 5.0 Hz, 5H); ^13^C NMR (101 MHz, CDCl_3_) δ 170.26, 165.76, 162.66, 153.53, 143.75, 142.22, 139.88, 130.66, 128.71, 127.95, 127.25, 115.79, 105.20, 76.02, 61.06, 56.33, 51.98, 51.62, 45.56, 42.06, 31.53, 27.97; HRMS (ESI^+^) calcd for C_37_H_44_N_4_O_6_ (*m*/*z* [M + H]^+^): 641.3339; found: 641.3338.

(*E)-1-(4-(bis(4-fluorophenyl)methyl)piperazin-1-yl)-4-(4-(3-(3,4,5-trimethoxyphenyl)acryloyl)piperazin-1-yl)butane-1,4-dione* (***B9***): white solid; yield: 77%; R_f_ = 0.9 (DCM/Me = 10:1, *v*/*v*); ^1^H NMR (400 MHz, CDCl_3_) δ 7.61 (d, *J* = 15.3 Hz, 1H), 7.35 (s, 1H), 7.33 (s, 1H), 7.32 (s, 1H), 7.31 (s, 2H), 6.99 (s, 1H), 6.97 (s, 2H), 6.95 (s, 2H), 6.72 (d, *J* = 13.2 Hz, 3H), 3.88 (d, *J* = 8.5 Hz, 9H), 3.79–3.65 (m, 5H), 3.60 (s, 4H), 3.52 (s, 2H), 2.94 (s, 1H), 2.87 (s, 1H), 2.67 (s, 4H), 2.38–2.35 (m, 2H); ^13^C NMR (101 MHz, CDCl_3_) δ 165.77, 163.25, 160.81, 153.56, 143.79, 139.93, 137.76, 130.68, 129.37, 129.29, 115.80, 115.58, 105.22, 74.35, 61.09, 56.35, 51.87, 51.51, 46.52, 45.53, 42.01, 31.55, 27.98; HRMS (ESI^+^) calcd for C_37_H_42_F_2_N_4_O_6_ (*m*/*z* [M + H]^+^): 677.3151; found: 677.3121.

*(E)-1-(4-(benzo[d]isothiazol-3-yl)piperazin-1-yl)-4-(4-(3-(3,4,5-trimethoxyphenyl)acryloyl)piperazin-1-yl)butane-1,4-dione* (***B10***): white solid; yield: 74%; R_f_ = 0.9 (DCM/Me = 10:1, *v*/*v*); ^1^H NMR (400 MHz, CDCl_3_) δ 7.89 (d, *J* = 8.4 Hz, 2H), 7.81 (t, *J* = 8.2 Hz, 2H), 7.62 (d, *J* = 15.3 Hz, 1H), 7.48 (ddd, *J* = 8.4, 7.0, 1.2 Hz, 1H), 7.42–7.31 (m, 2H), 3.88 (d, *J* = 9.2 Hz, 9H), 3.78 (s, 2H), 3.70 (s, 3H), 3.64 (s, 2H), 3.56 (s, 5H), 3.52 (d, *J* = 5.4 Hz, 3H), 2.91 (d, *J* = 29.1 Hz, 1H), 2.78 (s, 4H); ^13^C NMR (101 MHz, CDCl_3_) δ 170.67, 165.77, 163.49, 153.56, 152.97, 152.85, 143.81, 139.93, 130.68, 127.94, 127.88, 124.27, 124.07, 123.73, 120.79, 105.22, 61.09, 56.36, 50.26, 49.95, 45.24, 41.72, 28.10; HRMS (ESI^+^) calcd for C_31_H_37_N_5_O_6_S (*m*/*z* [M + H]^+^): 608.2543; found: 608.2543.

*(E)-1-(4-phenylpiperazin-1-yl)-4-(4-(3-(3,4,5-trimethoxyphenyl)acryloyl)piperazin-1-yl)butane-1,4-dione* (***B11***): yellow solid; yield: 68%; R_f_ = 0.7 (DCM/Me = 10:1, *v*/*v*); ^1^H NMR (400 MHz, CDCl_3_) δ 7.62 (d, *J* = 15.3 Hz, 1H), 7.30–7.26 (m, 2H), 6.94 (s, 2H), 6.92 (s, 2H), 6.74 (s, 2H), 3.89 (d, *J* = 9.2 Hz, 9H), 3.77 (t, *J* = 5.3 Hz, 3H), 3.70 (s, 8H), 3.67–3.61 (m, 4H), 3.20 (s, 1H), 2.75 (s, 4H); ^13^C NMR (101 MHz, CDCl_3_) δ 171.00, 170.44, 165.79, 153.55, 151.03, 143.81, 139.91, 130.67, 129.35, 120.65, 116.75, 115.77, 105.22, 61.08, 56.34, 49.72, 49.45, 45.41, 41.84, 29.79, 28.05; HRMS (ESI^+^) calcd for C_30_H_38_N_4_O_6_ (*m*/*z* [M + H]^+^): 551.2870; found: 551.2868.

*(E)-1-(4-(2,3-dichlorophenyl)piperazin-1-yl)-4-(4-(3-(3,4,5-trimethoxyphenyl)acryloyl)piperazin-1-yl)butane-1,4-dione* (***B12***): white solid; yield: 75%; R_f_ = 0.8 (DCM/Me = 10:1, *v*/*v*); ^1^H NMR (400 MHz, CDCl_3_) δ 7.61 (d, *J* = 15.3 Hz, 1H), 7.19–7.12 (m, 2H), 6.90 (dd, *J* = 7.7, 1.9 Hz, 1H), 6.76–6.70 (m, 3H), 3.87 (d, *J* = 9.6 Hz, 9H), 3.78 (s, 4H), 3.70 (s, 7H), 3.63 (s, 1H), 2.99 (s, 1H), 2.94 (s, 1H), 2.86 (s, 1H), 2.74 (s, 5H); ^13^C NMR (101 MHz, CDCl_3_) δ 170.97, 170.53, 165.76, 150.70, 143.78, 139.90, 134.27, 130.66, 127.83, 127.66, 125.29, 118.87, 115.76, 105.20, 61.07, 56.33, 51.62, 51.21, 45.69, 42.10, 31.53, 28.04; HRMS (ESI^+^) calcd for C_30_H_36_Cl_2_N_4_O_6_ (*m*/*z* [M + H]^+^): 619.2090; found: 619.2091.

*(E)-1-(4-(3-chlorophenyl)piperazin-1-yl)-4-(4-(3-(3,4,5-trimethoxyphenyl)acryloyl)piperazin-1-yl)butane-1,4-dione* (***B13***): yellow solid; yield: 74%; R_f_ = 0.8 (DCM/Me = 10:1, *v*/*v*); ^1^H NMR (400 MHz, CDCl_3_) δ 7.62 (d, *J* = 15.3 Hz, 1H), 7.18 (t, *J* = 8.1 Hz, 1H), 6.87–6.86 (m, 2H), 6.78 (dd, *J* = 8.5, 2.4 Hz, 2H), 6.74 (s, 2H), 3.88 (d, *J* = 9.0 Hz, 9H), 3.77 (s, 1H), 3.76 (s, 3H), 3.70 (s, 2H), 3.65–3.62 (m, 4H), 3.21 (s, 2H), 3.16 (t, *J* = 5.3 Hz, 3H), 2.91 (d, *J* = 29.1 Hz, 1H), 2.75 (s, 4H); ^13^C NMR (101 MHz, CDCl_3_) δ 170.62, 170.51, 165.80, 153.59, 152.06, 143.84, 139.99, 135.19, 130.69, 130.30, 120.24, 120.20, 116.47, 115.79, 114.52, 105.28, 61.11, 56.38, 51.96, 49.16, 48.96, 45.23, 41.65, 36.60, 29.82, 28.12, 28.07; HRMS (ESI^+^) calcd for C_30_H_37_ClN_4_O_6_ (*m*/*z* [M + H]^+^): 585.2480; found: 585.2480.

(E)*-1-(4-(4-(trifluoromethyl)phenyl)piperazin-1-yl)-4-(4-(3-(3,4,5-trimethoxyphenyl)acryloyl)piperazin-1-yl)butane-1,4-dione* (***B14***): yellow solid; yield: 71%; R_f_ = 0.8 (DCM/Me = 10:1, *v*/*v*); ^1^H NMR (400 MHz, CDCl_3_) δ 7.61 (d, *J* = 15.3 Hz, 1H), 7.50 (s, 1H), 7.48 (s, 1H), 6.93 (s, 1H), 6.91 (s, 1H), 6.75–6.70 (m, 3H), 3.89 (s, 6H), 3.87 (s, 3H), 3.78 (t, *J* = 5.3 Hz, 3H), 3.72 (t, *J* = 5.2 Hz, 1H), 3.69 (s, 9H), 3.63 (s, 2H), 3.29 (d, *J* = 12.4 Hz, 1H), 2.74 (s, 4H); ^13^C NMR (101 MHz, CDCl_3_) δ 165.78, 154.80, 153.57, 139.90, 130.67, 126.69, 126.00, 121.18, 115.37, 105.17, 61.14, 56.36, 48.54, 48.38, 45.03, 41.45, 28.11; HRMS (ESI^+^) calcd for C_31_H_37_F_3_N_4_O_6_ (*m*/*z* [M + H]^+^): 619.274.

*(E)-1-(4-(2-methoxyphenyl)piperazin-1-yl)-4-(4-(3-(3,4,5-trimethoxyphenyl)acryloyl)piperazin-1-yl)butane-1,4-dione* (***B15***): white solid; yield: 73%; R_f_ = 0.7 (DCM/Me = 10:1, *v*/*v*); ^1^H NMR (400 MHz, CDCl_3_) δ 7.61 (d, *J* = 15.3 Hz, 1H), 7.03 (s, 1H), 6.90 (s, 3H), 6.89 (s, 1H), 6.74 (s, 2H), 3.89 (s, 6H), 3.87 (s, 6H), 3.79 (s, 3H), 3.70 (s, 8H), 3.70 (s, 5H), 2.74 (d, *J* = 6.3 Hz, 4H); ^13^C NMR (101 MHz, CDCl_3_) δ 170.32, 165.75, 153.55, 152.34, 143.81, 140.76, 139.84, 130.69, 123.70, 121.16, 118.55, 115.76, 111.38, 105.14, 61.11, 56.34, 55.56, 51.01, 50.67, 45.72, 42.11, 29.82, 28.06; HRMS (ESI^+^) calcd for C_31_H_40_N_4_O_7_ (*m*/*z* [M + H]^+^): 581.2975; found: 581.2964.

*(E)-1-(4-(pyrimidin-2-yl)piperazin-1-yl)-4-(4-(3-(3,4,5-trimethoxyphenyl)acryloyl)piperazin-1-yl)butane-1,4-dione* (***B16***): white solid; yield: 76%; R_f_ = 0.9 (DCM/Me = 10:1, *v*/*v*);^1^H NMR (400 MHz, CDCl_3_) δ 8.32 (s, 1H), 8.31 (s, 1H), 7.61 (d, *J* = 15.3 Hz, 1H), 6.77–6.68 (m, 3H), 6.53 (td, *J* = 4.8, 2.3 Hz, 1H), 3.89 (s, 6H), 3.87 (s, 3H), 3.82 (dd, *J* = 6.6, 4.1 Hz, 2H), 3.69 (s, 6H), 3.62 (dd, *J* = 6.6, 4.1 Hz, 2H), 2.75 (dd, *J* = 7.8, 3.8 Hz, 4H), 1.75 (d, *J* = 3.7 Hz, 4H), 1.26 (d, *J* = 12.2 Hz, 2H); ^13^C NMR (101 MHz, CDCl_3_) δ 165.80, 161.62, 157.91, 153.56, 143.83, 139.93, 130.69, 115.78, 110.58, 105.23, 61.10, 56.36, 45.29, 43.75, 43.61, 41.74, 31.75, 29.81; HRMS (ESI^+^) calcd for C_28_H_36_N_6_O_6_ (*m*/*z* [M + H]^+^): 553.2775; found: 553.2775.

*(E)-1-(4-(6-fluorobenzo[d]isoxazol-3-yl)piperidin-1-yl)-4-(4-(3-(3,4,5-trimethoxyphenyl)acryloyl)piperazin-1-yl)butane-1,4-dione* (***B17***): white solid; yield: 71%; R_f_ = 0.8 (DCM/Me = 10:1, *v*/*v*);^1^H NMR (400 MHz, CDCl_3_) δ 7.67 (dd, *J* = 8.7, 5.1 Hz, 1H), 7.60 (d, *J* = 15.3 Hz, 1H), 7.25–7.21 (m, 1H), 7.05 (td, *J* = 8.8, 2.1 Hz, 1H), 6.76–6.69 (m, 3H), 3.88 (s, 6H), 3.86 (s, 3H), 3.70 (s, 3H), 3.63 (s, 1H), 3.33 (s, 2H), 3.31 (s, 1H), 3.28 (d, *J* = 12.9 Hz, 1H), 2.98–2.83 (m, 2H), 2.74 (s, 4H), 2.09 (dt, *J* = 16.2, 4.0 Hz, 2H), 1.87 (s, 4H); ^13^C NMR (101 MHz, CDCl_3_) δ 170.41, 165.79, 165.54, 164.08, 163.04, 153.53, 143.79, 139.89, 130.66, 122.51, 117.14, 115.78, 112.58, 105.21, 97.52, 61.07, 56.33, 45.39, 41.87, 34.41, 31.53, 30.47, 30.20, 28.15; HRMS (ESI^+^) calcd for C_32_H_37_FN_4_O_7_ (*m*/*z* [M + H]^+^): 609.2725; found: 609.2725.

### 2.2. Pharmacology

#### 2.2.1. Maximal Electroshock (MES) Test

In the MES test at 100 mg/kg via intraperitoneal administration, several TMCA piperazine amide derivatives demonstrated protective effects against tonic–clonic seizures at 0.5 h post-administration (Table 1). Compounds achieving complete protection (4/4 mice) included **A3**, **A9**, **A12**, **A14**, **B9**, and **B12**, indicating their potent activity in the early phase. While efficacy generally diminished over time, compounds **A3**, **A12**, and **A14** exhibited sustained activity at 3 h (1/4 protection), with **A14** uniquely maintaining complete protection at both 0.5 h and 1 h (4/4). Compound **B12** also showed residual activity at 3 h (1/4). The initial drug design, inspired by traditional Chinese medicine (TCM), revealed that compounds with moieties derived from *A. tatarinowii* and conjugated to TMCA piperazine exhibited anticonvulsant activity. Analogues **B3**, **A5** and **B5** exhibited favorable results (3/4 in both 0.5 and 1 h), suggesting this research preliminarily developed novel potential derivatives reflecting the function of initial pair herbs. Compounds **A7** and **B7** (3/4) introduced borneol, which partially elucidated the concept of compatibility in TCM. Despite the passage of time, natural products, including TCM, continue to provide valuable references for the development of antiepileptic drugs (AEDs).

Compounds demonstrating complete protection at 0.5 h were advanced to quantitative ED_50_ determination. As shown in Table 2, compound **B9** displayed the highest potency (ED_50_: 23.58 mg/kg), followed by compound **A9** (ED_50_: 37.46 mg/kg) and **A14** (ED_50_: 52.39 mg/kg). Crucially, the most promising candidate **B9** exhibited the highest protective index (PI: 9.97), significantly exceeding the stiripentol (STP) established PI threshold of 1.74 for standard therapeutic products (STPs). While compound **A9** also demonstrated a favorable PI value (8.14), compounds **A3** (PI: 6.05) and **A12** (PI: 5.20) exhibited lower safety margins compared to **B9**.

#### 2.2.2. Pentylenetetrazole (PTZ)-Induced Seizure Test

The anticonvulsant effects of selected TMCA piperazine amide derivatives **A3**, **A9**, **A12**, **A14**, **B9**, and **B12** were further evaluated against *sc*PTZ-induced seizures (Table 2). Compounds demonstrating complete protection (4/4) in the MES model at 0.5h were prioritized for *sc*PTZ assessment. Notably, compound **B9** exhibited the highest protective ratio (3/4 mice), followed by derivative **A14** (both 2/4), while analogue **A12** showed no protection (0/4) in this model. Regarding the latency period of epileptic seizures, most tested compounds exhibited activity in extending this latency. As the most promising compound, **B9** significantly prolonged seizure latency to 274.3 s, exceeding that of the reference drug STP and indicating robust suppression of seizure initiation. Initial structural analysis revealed that derivatives featuring arylpiperazine pharmacophores (e.g., *bis*(4-fluorophenyl)methylpiperazine moiety of compound **B9**) and rigid heterocyclic elements demonstrated enhanced efficacy in this model. The extended latency observed with compounds **B9** compared to **A12** further underscores the critical influence of *para*-fluorinated aryl groups on elevating seizure thresholds, potentially through enhanced receptor affinity or pharmacokinetic optimization [19].

#### 2.2.3. Acute Neurotoxicity Screening

In the neurotoxicity evaluation, heterocyclic substituted analogues **A3**, **A9**, **A12**, **A14**, **B9**, and **B12** showed clear neurotoxicity with the TD_50_ values ranging from 235 to 443 mg/kg, and the ester compound **A3** displayed weak neurotoxicity (Table 2). All the tested compounds exhibited higher PI values than the reference standard, among which compound **B9** was considered to be the most promising candidate according to the perspective of anticonvulsant activity and protective index.

#### 2.2.4. Validation of Anti-Seizure Potential of B9 Through EEG

**Figure 2:**
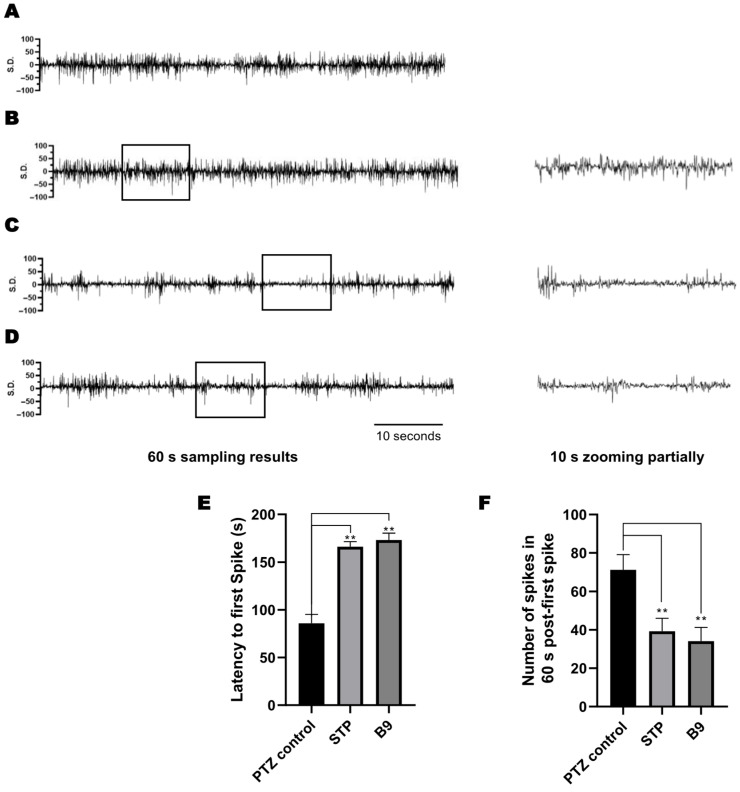
After pretreatment with STP and **B9** to the respective groups (*n* = 4), the animals were injected with PTZ. Typical EEG traces recorded during the experiment: (**A**) Representative portion of baseline EEG before administration of the convulsant; 60 s of electrical activity recorded after appearance of first epileptic spike post-PTZ injection, showing EEG of (**B**) PTZ control (PTZ only), (**C**) standard control (PTZ + STP) and (**D**) treatment group (**B9** + PTZ). All data are represented as mean ± SEM and ** indicates significance at *p* < 0.01. (**E**) Latency to the first epileptic spike and (**F**) the number of spikes in post-first spike 60 s were monitored with EEG.

The EEG results revealed that compound **B9** showed a coherent neurophysiological anticonvulsant profile: (1) early spike delay—similar to the effect of positive standard STP, compound **B9** extended the latency to the first PTZ-induced epileptic spike (Figure 2E); (2) spike density reduction—compound **B9** significantly reduced spike frequency within 60 s post-initial spike compared to PTZ controls (Figure 2D,F). Animal EEG spike density is a validated predictor of clinical seizure severity [20]. Dual efficacy of compound **B9** in suppressing both EEG spikes and behavioral convulsions positions it as a high-priority candidate for further development.

#### 2.2.5. Silver Staining Results

**Figure 3:**
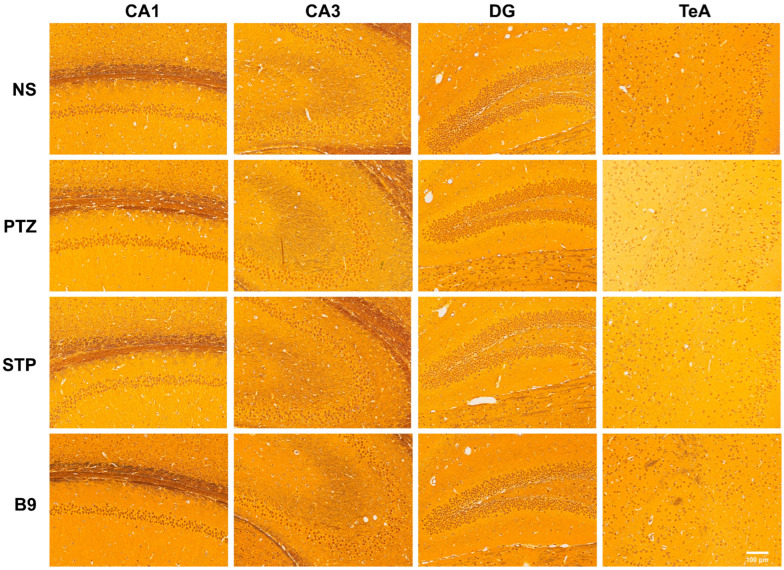
Silver staining of hippocampal tissue CA1, CA3, DG and temporal lobe in the control group, PTZ-induced seizure mice and treatment groups with STP and **B9** intervention (scale bar: 100 μm).

From the silver staining, we observed that the neuronal cell bodies in the CA1 and CA3 regions of the hippocampus in the PTZ group mice appeared blurry, with shorter axons and a significant reduction in the number of neurons compared with the control group (Figure 3). Conversely, the treatments of STP group and the **B9** group alleviated symptoms.

#### 2.2.6. Immunofluorescence Staining

**Figure 4:**
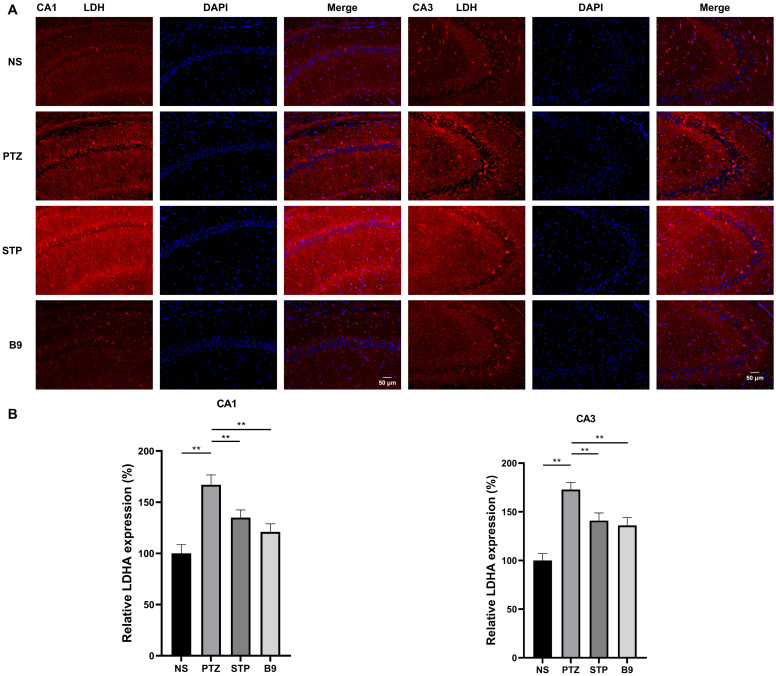
Immunofluorescence staining of hippocampal tissue CA1 and CA3 areas in the control group, PTZ-induced seizure mice and treatment groups intervened with STP and **B9** (scale bar: 50 μm). (**A**) Representative LDH immunofluorescence staining images and (**B**) quantification of fluorescence density of LDH in each group. All data are represented as mean ± SEM and ** indicates significance at *p* < 0.01.

The immunofluorescence analysis for LDH (Figure 4) showed in the CA1 a 1.6-fold significant increase in labeling in the PTZ group compared to NS (*p* < 0.05). The immunolabelling intensity in both SPT and **B9** groups was lower than the values found in the PTZ group (Figure 4). Similar data were also seen in the CA3 area, in which a respective 1.7-fold (*p* < 0.05) increase was present. Values were similar to those of the LDH level in both SPT and **B9** groups, providing evidence for potential of **B9** mediating LDH in PTZ-induced epilepsy mice.

#### 2.2.7. In Vitro LDH Assay

In order to investigate more potential mechanisms about LDH modulatory action of TMCA piperazine amide derivatives, in vitro enzymatic assay was performed. The active compounds **A3**, **A9**, **A12**, **A14**, **B9**, and **B12** were evaluated for the LDH inhibitory potency at the dose of 0.1 mM (Table 3). The result showed that compounds **A9** and **B9** exhibited a more than 50% inhibitory ratio while other derivatives were weaker inhibitors. Compared with the marketed anti-epilepsy drug STP targeting LDH and the original ligand for the LDH co-crystal complex GEN-140 (IC_50_: 0.003 μM) [21], the test analogues exhibited moderate inhibitory potency, representing a feasible route to develop promising LDH inhibitors with a novel skeleton.

#### 2.2.8. Molecular Docking Analysis

**Figure 5:**
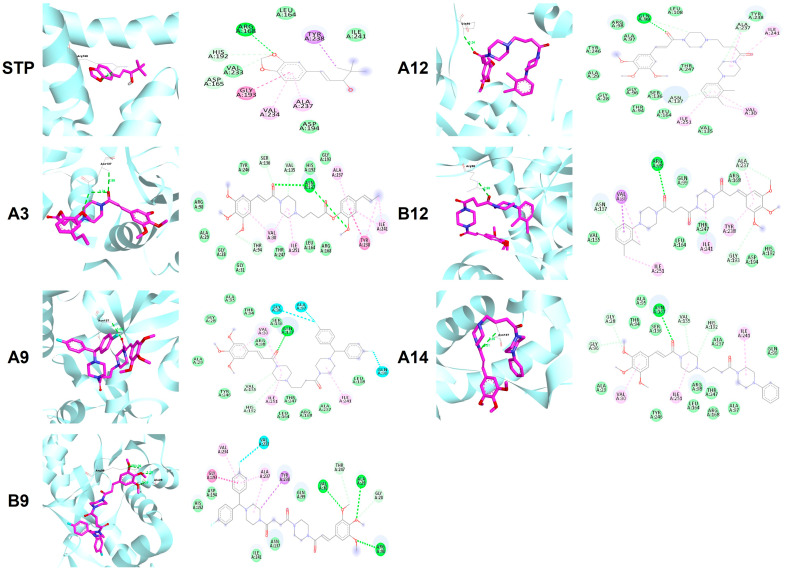
Docking mode of compounds **A3**, **A9**, **A12**, **A14**, **B9**, and **B12** at LDH (PDB cod: 4ZVV). The left columns are 3D interaction patterns, and the right columns are 2D interaction patterns in the binding pocket. The legend inset represents the type of interaction between the ligand atoms and the amino acid residues of the protein, and the important H-bonds.

Based on comprehensive pharmacological evaluation, the potential TMCA piperazine amide derivatives **A3**, **A9**, **A12**, **A14**, **B9**, and **B12** were systematically analyzed for their binding mechanisms with LDH through molecular docking studies, comparing with the positive drug STP. As quantified in Table 3, these compounds exhibited binding affinities ranging from −6.3 kcal/mol (**A3**) to −9.3 kcal/mol (**B9**), with all potential hydrogen bonds (<3 Å) indicating stable protein–ligand interactions (Figure 4). Crucially, the affinity ranking (**A3** < **A14** < **A12** < **B12** < **A9** < **B9**) demonstrated a direct correlation with their established anticonvulsant potency in MES and PTZ models, where **B9** showed exceptional activity surpassing the reference drug STP. Structural analysis revealed that the TMCA core anchors to LDH’s catalytic cleft through conserved interactions: the carbonyl group formed hydrogen bonds with key residues ASN 137 (**A9**, **A14**) and ARG 98 (**B9**, **B12**), while aromatic moieties engaged TYR 238 via π-stacking (**A3**, STP) and hydrophobic sub-pockets lined by ILE 241, ILE 251, and VAL 30 (Figure 5). Notably, **B9** achieved its superior affinity (−9.3 kcal/mol vs. STP’s −7.3 kcal/mol) by simultaneously occupying the NAD^+^-binding site (ARG 98 hydrogen bond) and the adjacent hydrophobic cavity (VAL 30/ALA 29 contacts). These findings establish the TMCA–piperazine scaffold as a novel chemotype for LDH modulation, with future optimization focusing on enhancing interactions with the defined pharmacophoric anchors to improve target engagement and anticonvulsant efficacy.

#### 2.2.9. MD Simulation

Based on comprehensive MD simulations and MM/GBSA analysis, derivative **B9** demonstrated superior binding stability and energetic favorability against LDH compared to the reference drug STP. Analysis of the trajectory revealed that compound **B9** maintained exceptional conformational stability within the enzyme’s catalytic cleft, as evidenced by ligand root-mean-square deviation (RMSD) values converging to 1.5 Å after 20 ns and persisting within this narrow threshold throughout the simulation. This remarkable structural fidelity substantially exceeded the stability profile of the reference compound STP, which exhibited RMSD fluctuations up to 2.8 Å. Critically, compound **B9** preserved its dual-residue binding mode: persistent hydrogen bonding interactions with ARG 98 anchored the ligand to the NAD^+^ cofactor binding site, while extensive hydrophobic contacts with residues VAL 30 and ALA 29 stabilized its occupation of the adjacent hydrophobic sub-pocket.

**Figure 6:**
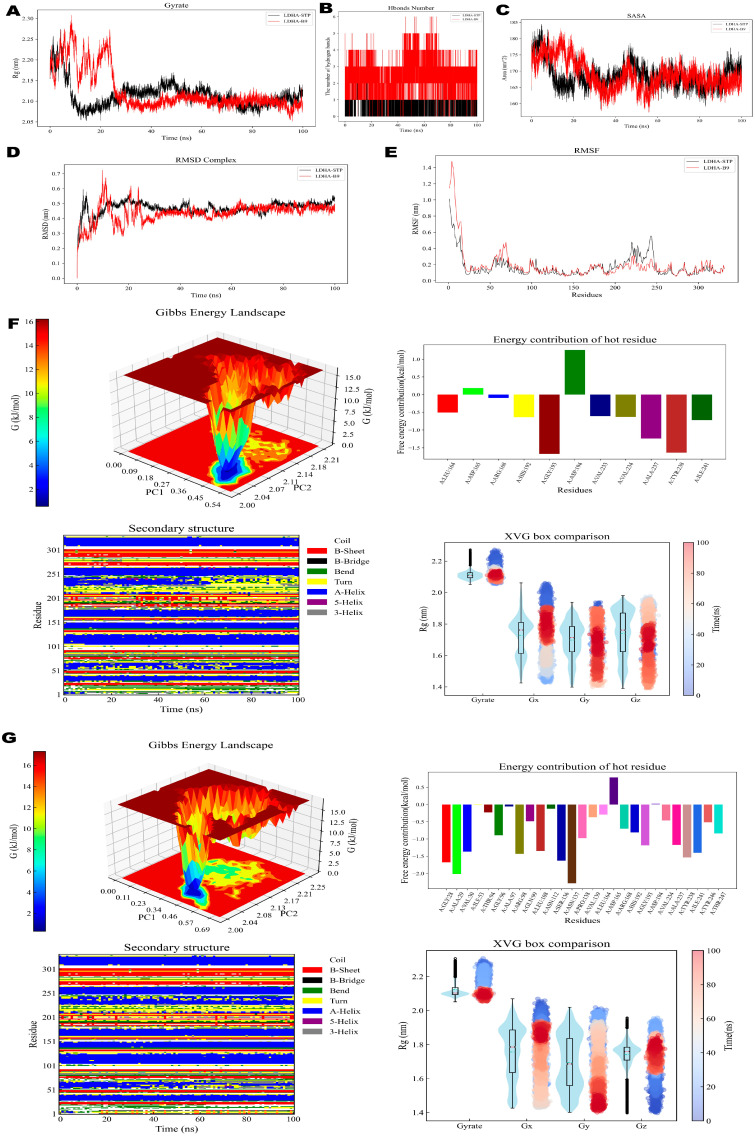
The 100 ns MD simulation results for STP and **B9**: (**A**) gyrate; (**B**) H-bond number; (**C**) SASA; (**D**) RMSD plot for the backbone atoms; (**E**) RMSF; (**F**) MM/GBSA free energy calculations for STP; (**G**) MM/GBSA free energy calculations for **B9**.

Complementary MM/GBSA free energy calculations (Table 4) quantified the thermodynamic drivers of this superior binding, yielding a net binding free energy (Δ_Gbind_) of −58.45 kcal/mol for **B9**-a threefold enhancement over STP (−19.16 kcal/mol). Deconvolution of energy components demonstrated that van der Waals interactions (Δ_EVDW_: −72.55 kcal/mol) constituted the dominant stabilizing force, primarily mediated through optimal shape complementarity with the VAL 30/ALA 29 hydrophobic cavity. Electrostatic contributions (Δ_Eelec_: −19.91 kcal/mol), largely attributable to the high-fidelity hydrogen bond with ARG 98, provided substantial additional stabilization despite partial compensation by polar solvation penalties (Figure 6). This energetically favorable binding profile directly correlated with exceptional functional performance of compound **B9**, including its >50% inhibition of LDH activity at 0.1 mM concentration in biochemical assays. The simultaneous occupation of both the NAD^+^-binding site and hydrophobic cavity explained the superior anticonvulsant efficacy of **B9** in MES and PTZ models. Collectively, these computational and experimental results established the TMCA–piperazine scaffold as a privileged chemotype for LDH modulation, with the **B9** binding paradigm informing future structure-based optimization strategies targeting residues 98, 30, and 29 to enhance hydrophobic packing and electrostatic complementarity.

#### 2.2.10. In Silico ADMET Predictions

Given that compound **B9** exhibited the highest potential for pharmacological effects, computational instruments (ADMET lab 3, VNN-ADMET, PreADMET, and PkCSM) were employed to forecast its pharmacokinetic characteristics. Based on the ADMET properties of compound **B9** summarized in Table 5, compound **B9** showed reduced solubility in water and compromised skin permeability, indicating difficulties in formulation for either parenteral or transdermal administration. Nonetheless, the observation of moderate Caco-2 permeability and significant absorption in the human intestines underscores its potential for oral bioavailability. This substance regularly acted as an inhibitor of P-gp, potentially affecting its distribution and the pattern of interactions between drugs. Research on its distribution indicated a minimal distribution volume (VDss) and successful penetration into the blood–brain barrier, despite the presence of contradictory computational data (PkCSM: log BB = −1.872). From a metabolic standpoint, compound **B9** demonstrated an inhibition effect on CYP2C9 and CYP3A4, exhibited an unclear level of inhibition on CYP1A2 (with some agreement), and showed no suppression of CYP2C19 or CYP2D6. The minimal overall clearance suggested a possibility for extended half-life. From a toxicological standpoint, compound **B9** showed no mutations (AMES negative) or skin sensitivity, yet it exhibited notable hERG inhibition, suggesting a cardiotoxicity risk that defies initial beliefs. Consequently, despite **B9**’s advantageous intestinal uptake and metabolic steadiness, its growth capabilities were offset by challenges in solubility, inconsistent evidence of CNS penetration, and significant hERG vulnerability. Furthermore, we studied the ADMET properties of compounds **A14** and STP as well (details in Supplementary Materials). The results revealed that active compounds screened from this study showed worse pharmaceutical properties compared to STP, especially in cardiac toxicity and water solubility.

#### 2.2.11. Structure–Activity Relationships (SAR)

**Figure 7:**
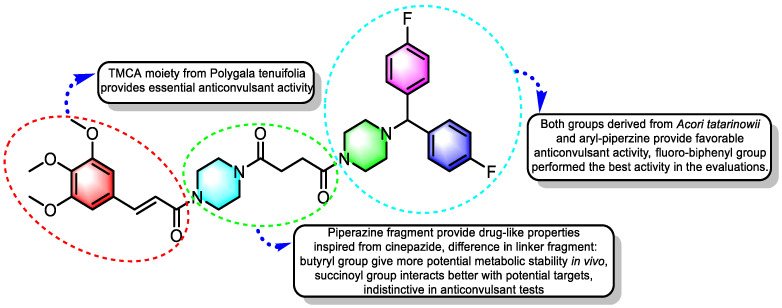
Preliminary SAR analysis of TMCA piperazine amide derivatives.

In general, we acquired hopeful anticonvulsant derivatives after our chemical modification. The SAR study can be summarized as follows (Figure 7): (1) TMCA moiety from *P. tenuifolia* provides essential anticonvulsant activity; (2) a piperazine fragment provides drug-like properties inspired by cinepazide, with a difference in the linker fragment, i.e., the butyryl group gives more potential metabolic stability in vivo and the succinoyl group interacts better with potential targets, indistinctive in anticonvulsant tests; (3) both groups derived from *A. tatarinowii* and aryl-piperzine provide favorable anticonvulsant activity in initial MES tests, where aryl-piperzine derivatives exhibited better effects in the PTZ model. The fluoro-biphenyl group performed the best in the evaluations. Specifically, both the electron-withdrawing group (the *para*-fluoro group) and the electron-donating group (the *ortho*-methoxy group) promote anticonvulsant activity.

## 3. Discussion

In search of better anticonvulsant agents, a cluster of novel derivatives of TMCA piperazine amide derivatives were synthesized through nucleophilic addition and amide coupling reactions. Cinnamyl piperazine has been disclosed as a hopeful bioactive motif in our previous article [22]; herein, the piperazine moiety is introduced to the analogues inspired by marketed drugs cinepazide and cinnarizine. Moreover, structural modification with TMCA from *P. tenuifolia* [8] and constituents from *A. tatarinowii* for anti-epilepsy agent development complies with our consistent previous research strategy. In this study, all the derivatives were screened for their anticonvulsant activities in vivo, among which compounds **A3**, **A9**, **A12**, **A14**, **B9**, and **B12** were found to exhibit significant anticonvulsive potential in the MES model; furthermore, using the scPTZ model, in silico docking and in vitro LDH inhibition, neurotoxicity of the mentioned compounds was investigated. The most promising candidate **(B9)** emerged and then EEG and silver staining were carried out to illustrate the anti-epilepsy property of compound **B9**. In addition, immunofluorescence staining and MD simulation were executed to prove the effects of **B9** modulating LDH. MES, PTZ and EEG models have been widely used as a primary drug screening test for anticonvulsant efficacy [23]. The MES test has been the most important model for clinical drug activity against tonic–clonic seizures because of its feasibility in the laboratory. The PTZ model is the most useful chemoconvulsant in the predictivity of clinical efficacy for seizures of the petit mal type according to its block inhibition or enhanced excitation of neurons.

As for the potential target, LDH was reported to play an important role in electrically regulating astrocyte–neuron lactate shuttle, suggesting the potential to identify metabolic enzymes that control epilepsy. Inhibition of the enzyme LDH hyperpolarized neurons, which was reversed by the downstream metabolite pyruvate. STP, along with its structurally modified derivative isosafrole with a necessary methylenedioxy-substituted allyl benzene skeleton, was revealed as novel group of antiepileptic drugs because of its unique antiepileptic mechanism of inhibiting LDH. Our previous research [13] proved that TMCA-asaronol ester extracted from *P. tenuifolia* and *A. tatarinowii* showed similar antiepileptic activity to STP, which provided the rationality of drug design of this article. In this study, we prepared potential derivative **B9** with superior activities to STP in both in vivo anti-epilepsy models and in vitro LDH inhibitory models. Above all, it could be concluded that the most promising compound **(B9)** is a worthwhile scaffold for further investigation in the development of anticonvulsant agents inspired by TCM.

## 4. Materials and Methods

### 4.1. Chemistry

The chemicals and reagents were purchased from several chemical companies such as Alfa-Aesar (Ward Hill, MA, USA) and Macklin (Shanghai, China). The progress of the reaction was monitored by thin layer chromatography analysis using silica gel G plates through a UV chamber at 256 nm for visualization of TLC spots. The mixtures were purified by flash column chromatography using silica gel. ^1^H NMR and ^13^C NMR spectra were obtained by a Bruker 400 NMR spectrometer (Bruker Corporation, Billerica, MA, USA) using CDCl_3_ as a solvent and TMS as an internal standard. The chemical shifts were expressed in ppm. Mass spectral ESI measurements were executed on SCIEX 5600 LC/MS instruments (SCIEX, Framingham, MA, USA).

#### General Procedure for the Synthesis of TMCA Peptide Derivatives

All titled compounds, including **A1–17** and **B1–17,** were successfully prepared through the synthetic protocols presented in Scheme 1. Core scaffold TMCA (1.0 equiv) was activated with EDCI (1.3 equiv) and DMAP (0.15 equiv) in DCM at rt (**Step a**) [24] to trigger condensation reaction conditions with piperazine (1.2 equiv). The intermediate underwent *O*-alkylation with alkyl halides (1.5 equiv) using K_2_CO_3_ (2.0 equiv) and catalytic KI in DMF at 75 °C (**Step b**) [25]. Subsequent hydrolysis with NaOH (2.0 M, 3.0 equiv) in MeOH/H_2_O (1:1) at rt (**Step c**) yielded carboxylic acid intermediates. EDCI (1.5 equiv)/DMAP (0.2 equiv) activation in DCM (rt, **Step d**), followed by coupling with amines (1.2 equiv) under EDCI/DMAP conditions (**Step e**), afforded targets **A2**–**A17**/**B2**–**B17**. The obtained product was washed with sodium bicarbonate water solution and diluted hydrochloric acid. The organic layer was dried over anhydrous sodium sulfate and evaporated to dryness to give the crude product. The mixtures were purified by flash column chromatography using silica gel in the system DCM/MeOH from 80:1 to 30:1.

### 4.2. Biological Activity

#### 4.2.1. Animals and Experimental Conditions

Male Kunming mice (23–28 g) were maintained under controlled conditions with ad libitum access to standard pellet diet and water. Test compounds were formulated immediately prior to administration in normal saline containing 0.5% Tween-80. Our experiment was authorized by the Institutional Animal Care and Use Committee of Shaanxi University of Chinese Medicine (animal ethics certificate number: SUCMDL20210309027).

#### 4.2.2. Maximal Electroshock (MES) Test

The MES test carried out following the method of Swinyard [26]. A 60 Hz current (50 mA; 0.25 s duration) was delivered via corneal electrodes using a YSD-4G electro convulsion meter (Zhenghua Instruments, Huaibei, China, SN: ZH0056232). Stiripentol (STP, 100 mg/kg in 1% *v*/*v* Tween-80/0.9% NaCl) served as the positive control. Test compounds were suspended in an identical vehicle. Protection was defined as abolition of tonic hindlimb extension. Test groups received intraperitoneal (i.p.) injections of compounds at 100 mg/kg for preliminary screening, followed by 75, 50, 25, and 12.5 mg/kg for ED_50_ determination.

#### 4.2.3. Pentylenetetrazole (PTZ)-Induced Seizures

Seizures were induced by subcutaneous (s.c.) administration of PTZ (85 mg/kg in 1% *v*/*v* Tween-80/0.9% NaCl), 30 min after compound treatment. This dose produces clonic seizures (≥5 s duration) in 97% of naive mice. Latency to seizure onset was recorded during continuous observation [27]. Compounds demonstrating complete protection at 100 mg/kg (i.p.) were evaluated for latent period prolongation.

#### 4.2.4. Acute Neurotoxicity Screening

Neurotoxicity was assessed via a rotarod test [28]. Mice were trained to maintain balance on a rotating rod (diameter: 4 cm; 24 rpm). Trained animals received i.p. injections of test compounds or reference drugs. At 30 min post-administration, neurologic impairment was defined as failure to remain on the rod for 3 min (≥3 falls within the testing period). Data were used to calculate TD_50_ values.

#### 4.2.5. Electroencephalogram (EEG)

Mice sedated with urethane were subjected to aseptic craniotomy, involving the stereotaxic insertion of cortical screw electrodes aligned with bregma coordinates (anterior–posterior: +3.0 mm; mediolateral: ±1.5 mm) and a reference electrode (anterior–posterior: −2.0 mm; mediolateral: −1.5 mm). The electrodes were secured using dental acrylic, followed by a recovery period of 5–7 days for the animals before the instrumentation. The continuous EEG data were captured through using 8-channel bioamplifiers linked to a PowerLab 8/30 system (AD Instruments, Shanghai, China). After a 30 min acclimatization period in the recording chambers, initial activity levels were documented for 15–20 min. The experimental substances (**STP** and **B9**, 100 mg/kg) were delivered intraperitoneally. After 60 min, pentylenetetrazol (PTZ, 85 mg/kg i.p.) was administered, initiating seizure. Sampling of EEG signals occurred at a frequency of 200 Hz, utilizing a bandpass filter ranging from 0.1 to 60 Hz [29]. Electrographic convulsions were characterized by spikes surpassing double the baseline amplitude, quantified by (1) the delay before the initial spike (seconds after PTZ); (2) the frequency of spikes (counts per 60 s period from the first spike) through established peak-detection methods.

#### 4.2.6. Silver Staining

Following the creation of the PTZ model, a modified version of the silver impregnation staining technique was applied for silver staining, enabling the observation of changes in neuronal fibers and synapses throughout diseases [30]. Brain tissue slices from each mouse, encased in paraffin, underwent deparaffinization using xylene and absolute ethanol, followed by a treatment in acidic formaldehyde, and then incubation in a glycine–silver mixture. Following the application of a reducing solution for staining, the samples were analyzed using an optical microscope. Against a yellow backdrop, nerve cells’ axons, dendrites, collagen fibers, and neurofibrillary tangles were colored in dark brown or black.

#### 4.2.7. Immunofluorescence Staining

Brain samples were extracted, preserved in 4% paraformaldehyde for a day, and subsequently immersed in a 30% sucrose solution to dehydrate. Post OCT embedding, slices of brain tissue measuring 16 μm in thickness were sliced using a cryostat. Following the permeabilization using 0.5% TritonX-100 and subsequent blocking with 10% goat serum at ambient temperature to remove nonspecific staining, the tissue samples were subjected to overnight incubation at 4 °C with the primary antibody, LDHA (Proteintech (Rosemont, IL, USA), Cat No. 19987-1-AP, Lot: 00106920, rabbit anti-rat 1:200). On the next day, the slides underwent incubation using a secondary antibody. The slides underwent counterstaining using DAPI. Ovarian slices were examined and visualized utilizing the VS200-BU Slide Scanner (Olympus Corporation, Tokyo, Japan).

#### 4.2.8. Statistical Analysis

All data were presented as mean ± standard deviation. The results were analyzed by a one-way ANOVA, followed by Student’s two-tailed *t*-test for comparison between test and control, and Dunnett’s test when the data involved three or more groups. The level of significance for all tests was set at *p* < 0.05.

#### 4.2.9. In Vitro LDH Assay

Employing the commercial LDH enzyme (Solarbio Life Science, Beijing, China, CAS:9001-60-9, Lot. Document No. 14240222001) includes a colorimetric assay kit designed to assess the synthesized substances’ capacity to suppress the LDH enzyme, using established techniques with slight alterations [31,32]. In summary, a 96-well microplate was filled with 30 μL of LDH enzyme (300 U/mL) and 70 μL of the inhibitor mixture (0.1 mM in final concentration). Following a 10 min pre-incubation process at ambient temperature, 110 μL of NADH (0.2 mM) was introduced. Subsequently, each well received 40 μL of phosphate buffer (20 mM, pH 6.8), reaching a total volume of 250 μL, followed by a 10 min incubation of the assay plate at 28 °C. Subsequently, dopachrome’s absorbance was gauged at 340 nm utilizing a SpectraMax 190 Absorbance Microplate Reader (Molecular Devices, San Jose, CA, USA). STP (0.1 mM) served as a benchmark inhibitor, while phosphate buffer functioned as the negative control. In the initial screening, the level of suppression by the test substances was denoted as a percentage (%). The calculation for the LDH inhibition ratio was as follows:

Inhibition (%) = [(A_sample_ − A_blank_)/A_control_] × 100%

A_sample_ denotes the OD340 absorbance of test compound and A_blank_ is the OD340 absorbance for the blank. A_control_ represents the OD_340_ absorbance of control.

#### 4.2.10. Molecular Modeling

The crystal structure of lactate dehydrogenase (LDH; PDB: 4ZVV) was retrieved from the Protein Data Bank [21]. Flexible docking simulations were performed using AutoDock Vina 1.1.2 to analyze interactions between the LDH active site and seven compounds (**STP**, **A3**, **A9**, **B9**, **A12**, **B12**, **A14**) [33]. The previous preparatory work for the complex was completed by the Amdock tool 4.2.6 [34]. The docking grid center was defined by the original ligand GEN-140 (X: 41.554545, Y: 14.328606, Z: 25.699061) with a 15 Å search radius [35]. Key active site residues (ASN 137, ASP 165, ARG 168, HIS 192, GLY 193, ASP 194, ALA 237, TYR 238, ILE 241, GLY 245, THR 247)—known to interact with native ligand GEN-140—were set as rotatable to enable conformational sampling. To ensure the rationality of the docking results, we also performed the docking of the original ligand GEN-140 and compared the RMSD value of the conformation obtained from the docking with that of the original ligand conformation (results shown in Supplementary Materials). Binding affinity (kcal/mol) and intermolecular interactions (hydrogen/hydrophobic bonds) were quantified through Discovery Studio Visualizer.

#### 4.2.11. Molecular Dynamic (MD) Simulation

The optimal docking pose of compounds **B9** and STP was selected for molecular dynamics (MD) simulations using GROMACS 2022.3. The ligand–protein complex was solvated in a TIP3P water box, energy-minimized, and equilibrated in NVT/NPT ensembles prior to 100 ns production MD simulation (2 fs timestep, 300 K, NPT ensemble). Simulations employed the AMBER force field with conformations sampled at discrete time points to assess ligand binding stability via VMD visualization. Binding free energies were calculated by MM/PBSA analysis with energy decomposition in GROMACS [36,37].

#### 4.2.12. In Silico ADMET Predictions

To evaluate the pharmacokinetic properties of the most promising compounds (**B9**, **A14** and STP), in silico ADMET predictions were conducted using several reliable prediction servers, including Preadmet (http://preadmet.bmdrc.kr/ accessed on 16 June 2025), vNN-ADMET (https://vnnadmet.bhsai.org/ accessed on 16 June 2025), pKCSM (http://biosig.unimelb.edu.au/pkcsm/ accessed on 16 June 2025), and ADMETlab 3.0 (https://admetmesh.scbdd.com/ accessed on 16 June 2025). A consensus prediction approach was employed, where a property was considered valid if it was indicated by more than two servers [38]. This method ensures higher confidence in the predicted pharmacokinetic profiles of the compounds. In brief, the chemical structures of the compounds were prepared in SMILES format and uploaded to each server. ADMET properties, including absorption, distribution, metabolism, excretion, and toxicity, were selected across all tools. Key pharmacokinetic properties evaluated include Caco-2 permeability, skin permeability, blood–brain barrier (BBB) penetration, volume of distribution (VDss), cytochrome P450 (CYP) inhibition, AMES toxicity and hERG inhibition.

## Figures and Tables

**Table 1:** Anticonvulsant activity in the MES model (100 mg/kg) of compounds administered i.p. to mice.

Com.	0.5 h	1 h	2 h	3 h	Com.	0.5 h	1 h	2 h	3 h
Ctrl	0/4 ^a^	0/4	0/4	0/4					
STP	4/4	2/4	1/4	0/4					
**A1**	1/4	1/4	0/4	0/4	**B1**	1/4	1/4	0/4	0/4
**A2**	2/4	2/4	1/4	0/4	**B2**	3/4	3/4	1/4	0/4
**A3**	4/4	3/4	2/4	1/4	**B3**	3/4	3/4	1/4	0/4
**A4**	2/4	2/4	1/4	0/4	**B4**	3/4	2/4	2/4	0/4
**A5**	3/4	3/4	2/4	0/4	**B5**	3/4	3/4	2/4	0/4
**A6**	2/4	2/4	1/4	0/4	**B6**	2/4	1/4	1/4	0/4
**A7**	3/4	2/4	1/4	0/4	**B7**	3/4	2/4	1/4	0/4
**A8**	3/4	2/4	1/4	0/4	**B8**	3/4	3/4	1/4	0/4
**A9**	4/4	3/4	2/4	0/4	**B9**	4/4	3/4	1/4	0/4
**A10**	3/4	1/4	1/4	0/4	**B10**	3/4	3/4	1/4	0/4
**A11**	1/4	1/4	1/4	0/4	**B11**	2/4	2/4	1/4	0/4
**A12**	4/4	3/4	3/4	1/4	**B12**	4/4	3/4	1/4	1/4
**A13**	1/4	1/4	0/4	0/4	**B13**	2/4	1/4	0/4	0/4
**A14**	4/4	4/4	3/4	1/4	**B14**	3/4	3/4	2/4	0/4
**A15**	3/4	3/4	1/4	0/4	**B15**	3/4	3/4	1/4	0/4
**A16**	3/4	3/4	2/4	0/4	**B16**	3/4	2/4	2/4	0/4
**A17**	2/4	2/4	1/4	0/4	**B17**	2/4	2/4	1/4	0/4

Notes: ^a^ protected mice/total mice.

**Table 2:** Further screening for some active compounds.

Com.	MES ED_50_	*_SC_*PTZ		TD_50_	PI ^a^ (MES)
		Protective Ratio (100 mg/kg)	Latent Time (100 mg/kg, s)		
**NS**	-	0/4	150.8 ± 16.3	-	-
**STP ^b^**	277 (1182)	2/4	269.5 ± 19.8 **	161.7 (146.2–256.2)	1.41
**A3**	73.31 (41.96–92.14)	1/4	222.0 ± 14.7 **	443.42 (327.60–626.48)	6.05
**A9**	37.46 (16.51–78.97)	1/4	204.5 ± 12.6 **	304.79 (188.99–474.43)	8.14
**B9**	23.58 (10.33–71.72)	3/4	274.3 ± 17.2 **	235.04 (107.18–373.21)	9.97
**A12**	54.71 (29.90–85.28)	0/4	161.5 ± 24.0	284.38 (173.91–448.98)	5.20
**B12**	60.27 (38.71–84.89)	1/4	230.3 ± 17.4 **	417.16 (293.63–614.75)	6.92
**A14**	52.39 (28.48–83.43)	2/4	233.5 ± 18.3 **	358.26 (231.95–528.49)	6.84

Notes: ^a^ PI: protective index (TD_50_/ED_50_); ^b^ reference drug, data from [13]; **: *p* < 0.01 vs. control; 95% confidence intervals given in parentheses.

**Table 3:** Molecular docking score and LDH inhibitory evaluation for active compounds in vitro.

Com.	Binding Affinity (Kcal/mol)	Key Interactions	LDH Inhibition Ratio (0.1 mM)
**STP**	−6.5	ARG 168 (H-bond), TYR 238 (Pi-Sigma), GLY 193 (Amide-Pi Stacked), VAL 234, VAL 237 (Pi-Alkyl), VAL 233, HIS 192 (Carbon Hydrogen Bond)	46.1 ± 6.2%
**A3**	−6.3	ASN 137 (H-bond), TYR 238 (Pi-Pi T-shaped), SER 136, VAL 135, THR 94 (Carbon Hydrogen Bond)	37.6 ± 8.1%
**A9**	−8.5	ASN 137 (H-bond), VAL 90, ILE241, ILE 251 (Pi-Alkyl), GLY96, ALA 97, GLN 99 (Halogen), ARG 98, VAL 135, HIS 192 (Carbon Hydrogen Bond)	52.6 ± 8.5%
**B9**	−9.3	VAL 30, ALA 29, ARG 98 (H-bond), TYR 238 (Pi-Sigma), VAL 233 (Halogen), GLY 193 (Amide-Pi Stacked), THR 247, GLY 28, VAL 234 (Carbon Hydrogen Bond)	54.9 ± 9.7%
**A12**	−7.6	GLN 99 (H-bond), ILE 241, ILE 251, VAL 30 (Alkyl)	41.9 ± 9.2%
**B12**	−7.7	ARG 98 (H-bond), ALA 237, VAL 30, ILE 251 (Alkyl)	44.3 ± 6.5%
**A14**	−6.9	ASN 137 (H-bond), VAL 30, ILE 251, ILE 241 (Alkyl), SER 136, HIS 192, GLY 96, GLY 28 (Carbon Hydrogen Bond)	42.6 ± 7.3%

**Table 4:** MM/GBSA free energy calculations for STP and **B9**.

Contribution Components	LDH-STP	LDH-B9
Δ_VDWAALS_	−29.93 ± 0.04	−72.55 ± 1.86
ΔE_elec_	−4.87 ± 0.01	−19.91 ± 0.77
ΔE_GB_	19.63 ± 0.14	43.11 ± 1.25
ΔE_surf_	−3.99 ± 0.01	−9.09 ± 0.19
ΔG_gas_	−34.81 ± 0.04	−92.47 ± 2.02
ΔG_solvation_	15.65 ± 0.14	34.02 ± 1.26
ΔTotal	−19.16 ± 0.15	−58.45 ± 2.38

**Table 5:** ADMET properties of compound **B9**.

Category	Property	ADMET Lab 3	VNN-ADMET	PreADMET	PkCSM	Consensus
**Absorption**	Water solubility		-	389.712 mg/L	−4.246 log mol/L	Low
	Caco-2 permeability	−5.454 log unit	-	52.768 nm/s	0.856 log Papp in 10^−6^ cm/s	Moderate
	Human intestinal absorption (%)	-	-	97.683	65.214	High
	Skin permeability (log Kp)	-	-	−2.563	−2.735	Low
	P-gp inhibitor	Yes	No	Yes	Yes	Yes
**Distribution**	VDss (human, log L/kg)	-	-	-	0.674	Low
	BBB permeability (log BB)	Yes	-	Yes	−1.872	Yes
**Metabolism**	CYP1A2 inhibitor	No	Yes	-	No	Partial
	CYP2C9 inhibitor	Yes	No	Yes	Yes	Yes
	CYP2C19 inhibitor	Yes	No	No	No	No
	CYP2D6 inhibitor	Yes	No	No	No	No
	CYP3A4 inhibitor	Yes	No	Yes	Yes	Yes
**Excretion**	Total clearance (log ml/min/kg)	-	-	-	0.367	Low
**Toxicity**	AMES toxicity	No	-	No	No	No
	hERG blocker	Yes	Yes	Medium risk	No	Yes

## Data Availability

The original contributions presented in this study are included in the article/Supplementary Material. Further inquiries can be directed to the corresponding authors.

## Supplementary Materials

The following supporting information can be downloaded at https://www.mdpi.com/article/10.3390/ph18091312/s1. HRMS and NMR spectra of derivatives **A2–17** and **B2–17**.
