# Supplementary material for: Excavating Precursors from Herb Pairs Polygala tenuifolia and Acori tatarinowii: Synthesis and Anticonvulsant Activity Evaluation of 3,4,5-Trimethoxycinnamic Acid (TMCA) Piperazine Amide Derivatives
Source: Pharmaceuticals (Basel). 2025 Sep 1;18(9):1312. doi: 10.3390/ph18091312 (PMC12472621; doi:10.3390/ph18091312)
Supplement: Supplementary file 1 [file pharmaceuticals-18-01312-s001.zip › Supplementary Materials.pdf]

Supporting information

Excavating precursors from pair herbs *Polygala tenuifolia* and *Acori tatarinowii*: Synthesis, anticonvulsant activity evaluation of 3,4,5-trimethoxycinnamic acid (TMCA) piperazine amide derivatives

*Zefeng Zhao [a, *], Mengchen Lei [a], Yongqi Wang [a], Yujun Bai [b] and Haifa Qiao [a, **]*

[a] Shaanxi University of Chinese Medicine, Xianyang, Shaanxi Province 712046, P. R. China;

[b] Northwest University, 229 Taibai Road, Xi'an, 710069, P. R. China;

** Correspondence: 2061046@sntcm.edu.cn and 1511006@sntcm.edu.cn.*

NMR spectrum of synthesized derivatives

*4-allyl-2-methoxyphenyl (E)-4-(4-(3-(3,4,5-trimethoxyphenyl)acryloyl)piperazin-1-yl)butanoate (**A2**):*

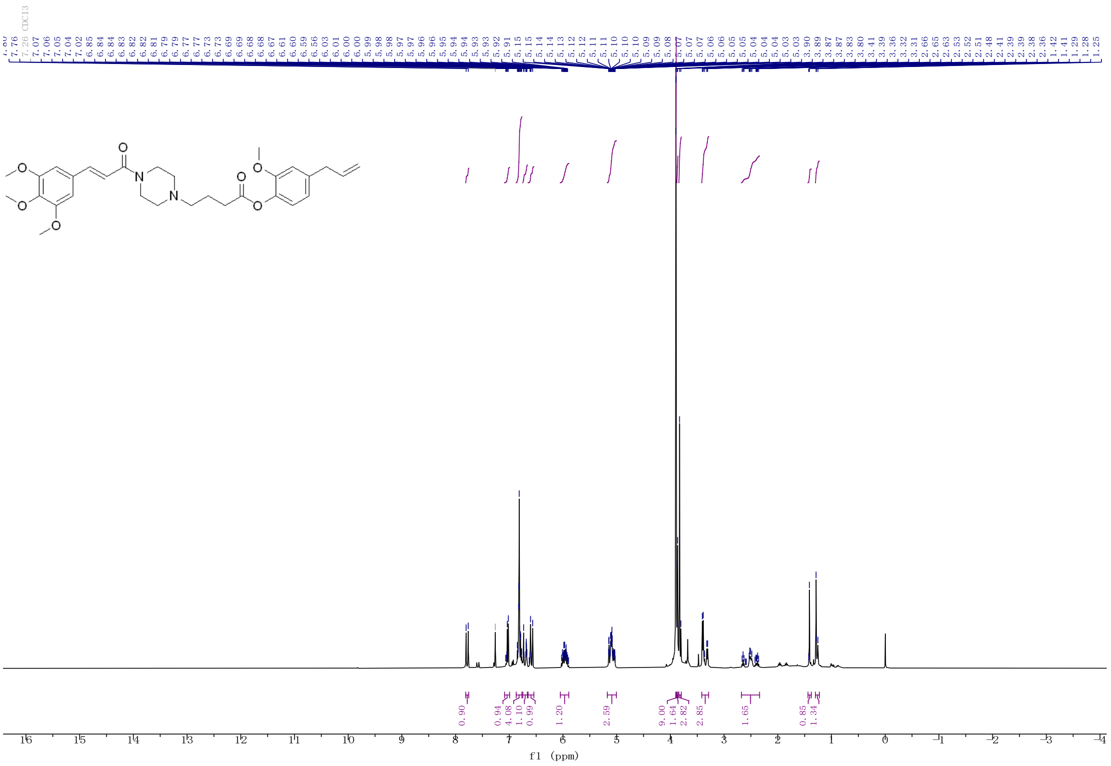

^{1}H NMR (600 MHz, $CDCl_3$) spectrum of **A2**

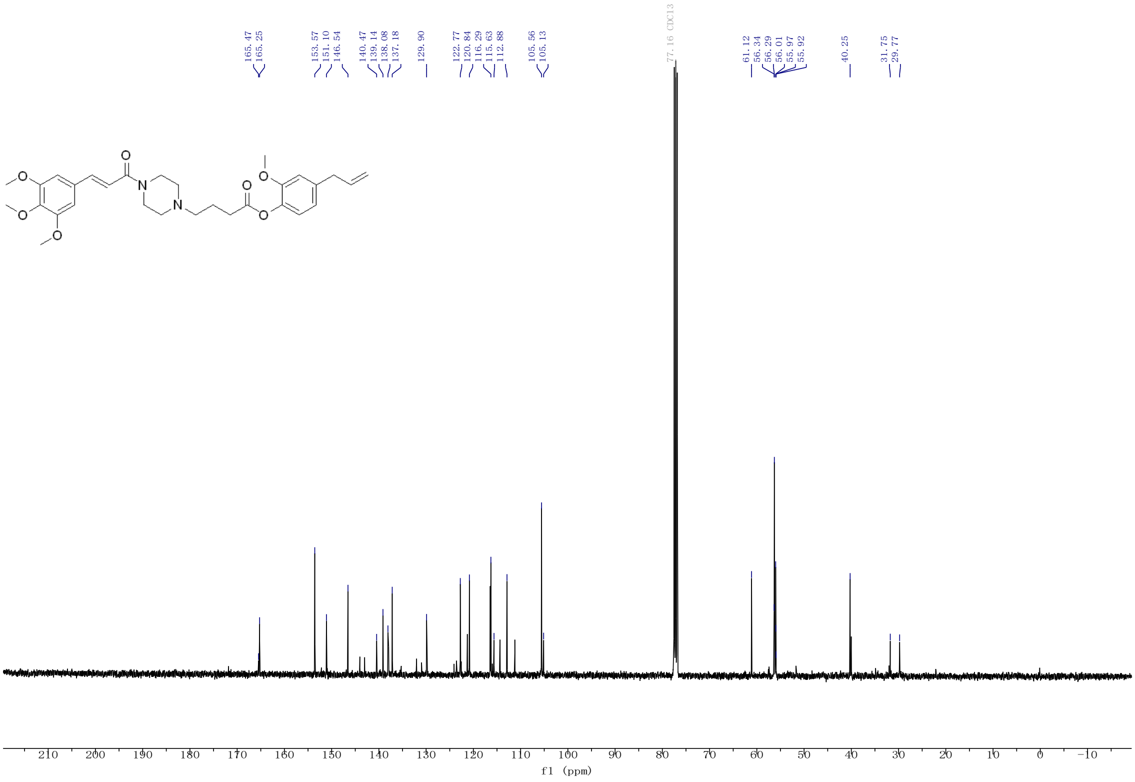

^{13}C NMR (600 MHz, $CDCl_3$) spectrum of **A2**

*5-allyl-2-methoxyphenyl (E)-4-oxo-4-(4-(3-(3,4,5-trimethoxyphenyl)acryloyl)piperazin-1-yl)butanoate (**B2**):*

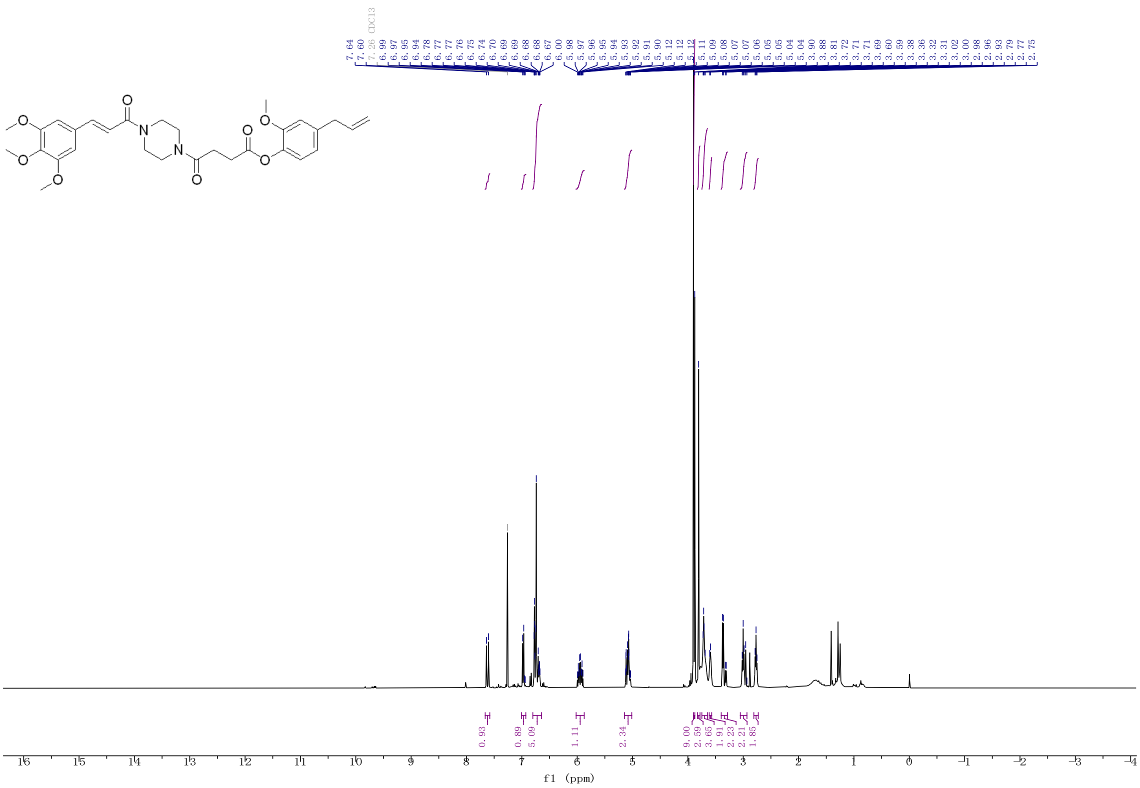

^{1}H NMR (600 MHz, $CDCl_3$) spectrum of **B2**

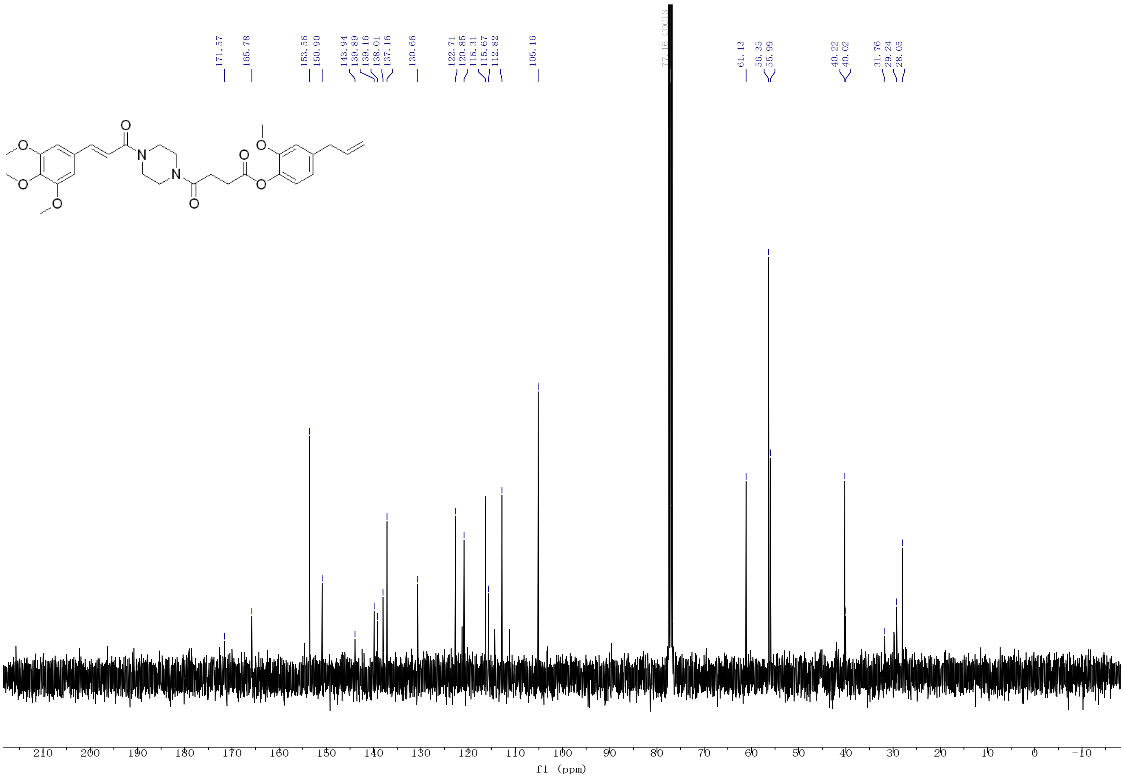

^{13}C NMR (600 MHz, $CDCl_3$) spectrum of **B2**

*methoxy-4-((E)-prop-1-en-1-yl)phenyl 4-(4-((E)-3-(3,4,5-trimethoxyphenyl)acryloyl)piperazin-1-yl)butanoate (**A3**):*

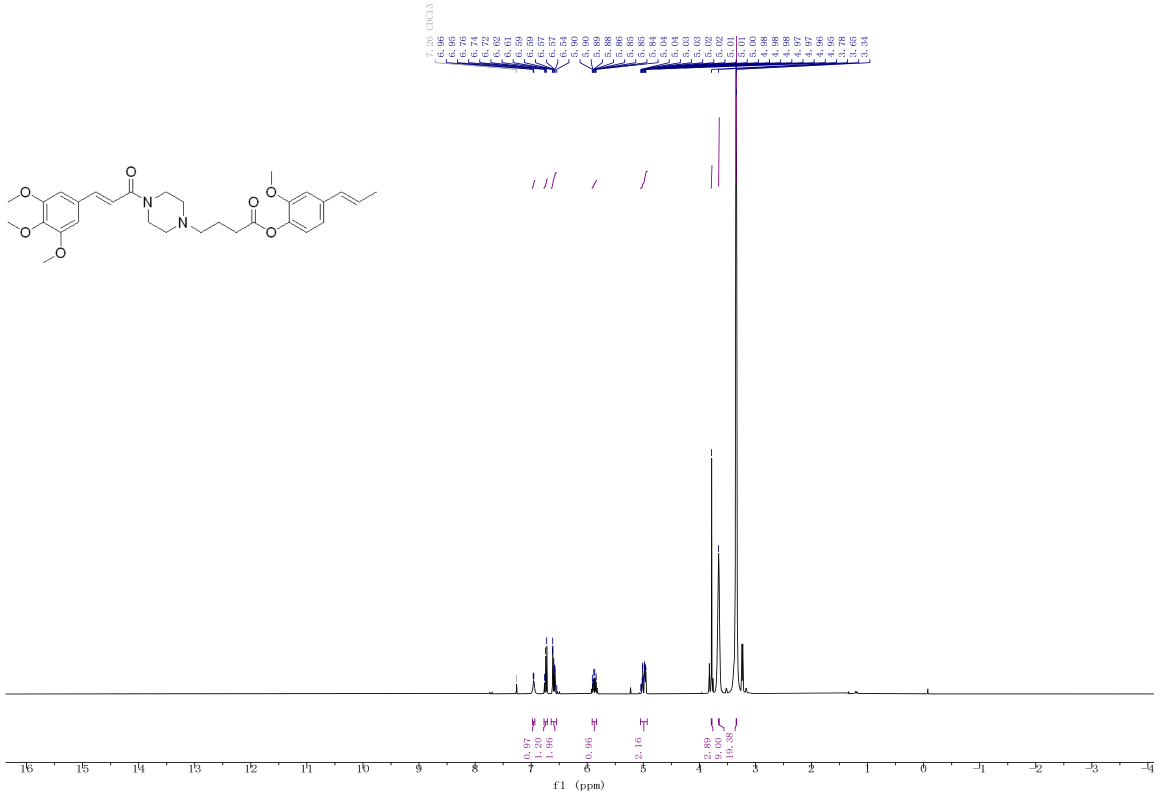

^{1}H NMR (600 MHz, $CDCl_3$) spectrum of **A3**

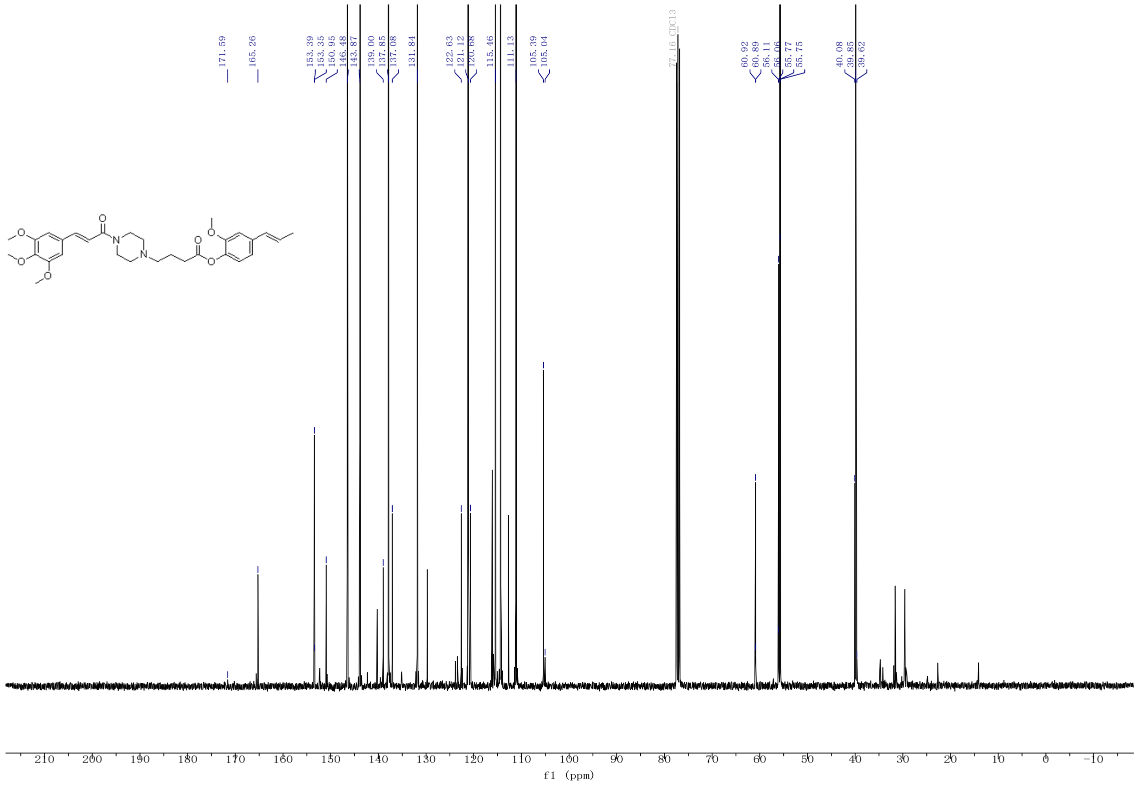

^{13}C NMR (600 MHz, $CDCl_3$) spectrum of **A3**

*2-methoxy-4-((E)-prop-1-en-1-yl)phenyl 4-oxo-4-(4-((E)-3-(3,4,5-trimethoxyphenyl)acryloyl)piperazin-1-yl)butanoate (**B3**):*

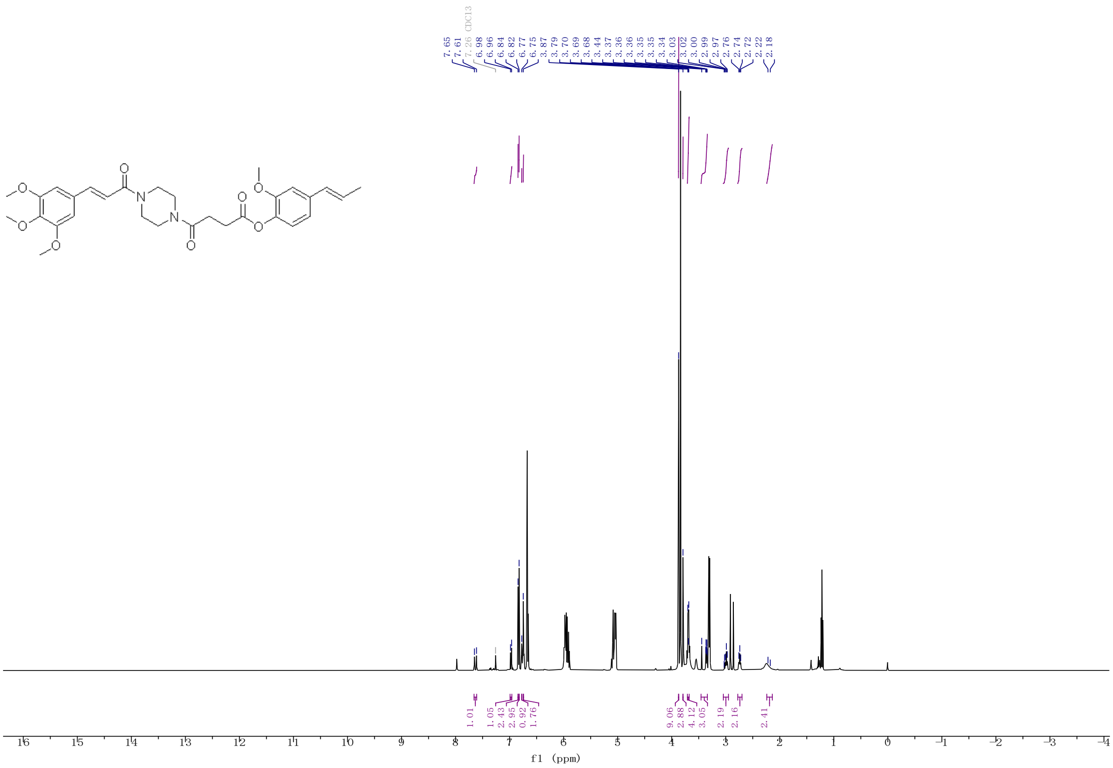

^{1}H NMR (600 MHz, $CDCl_3$) spectrum of **B3**

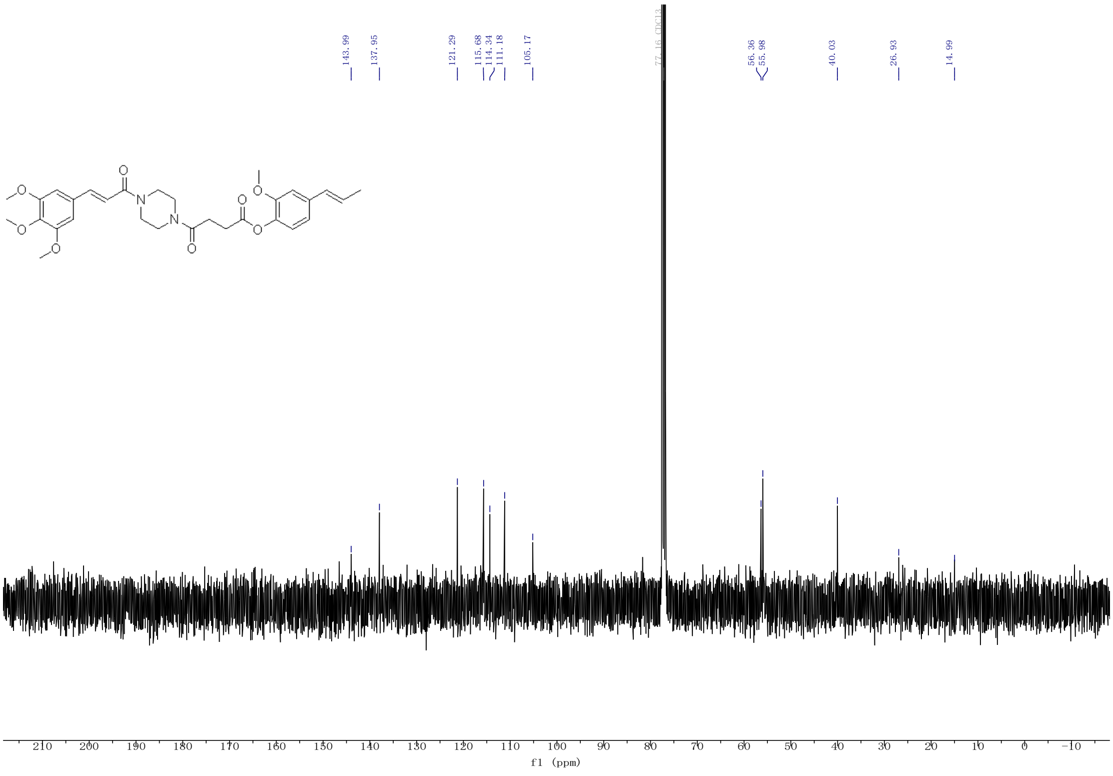

^{13}C NMR (600 MHz, $CDCl_3$) spectrum of **B3**

*2-allylphenyl (E)-4-(4-(3-(3,4,5-trimethoxyphenyl)acryloyl)piperazin-1-yl)butanoate (**A4**):*

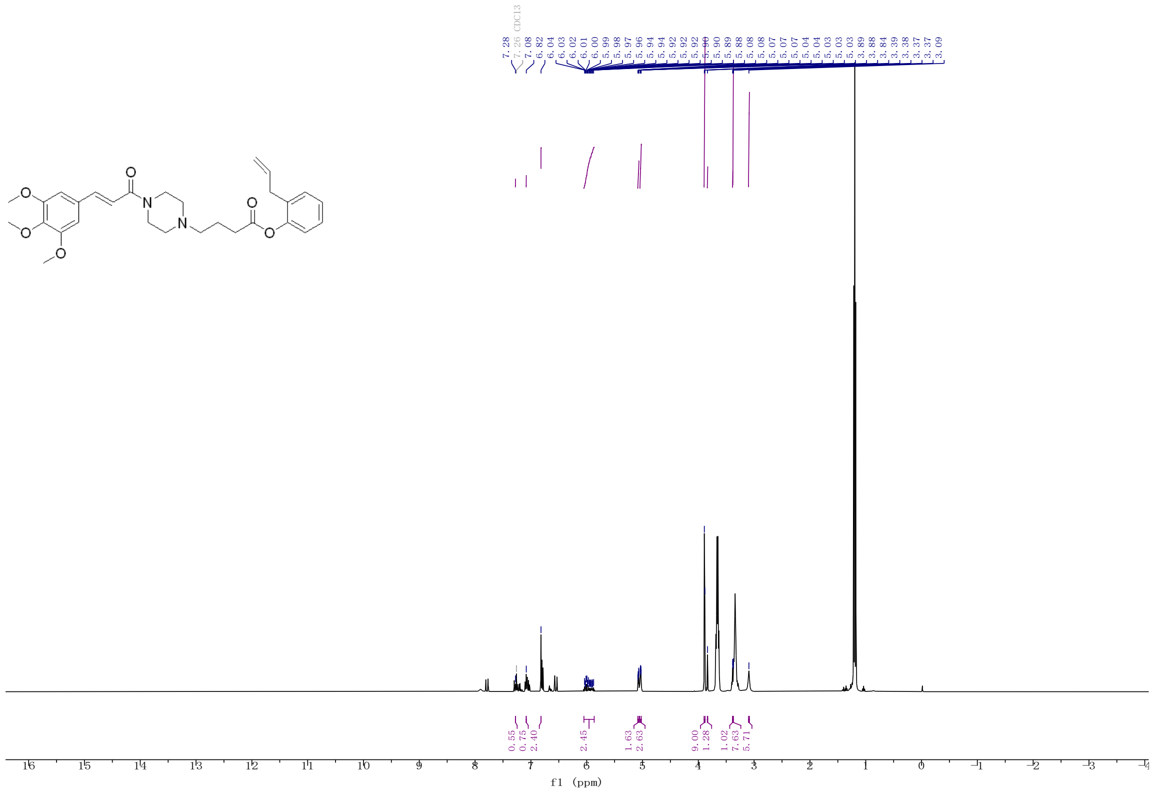

^{1}H NMR (600 MHz, $CDCl_3$) spectrum of **A4**

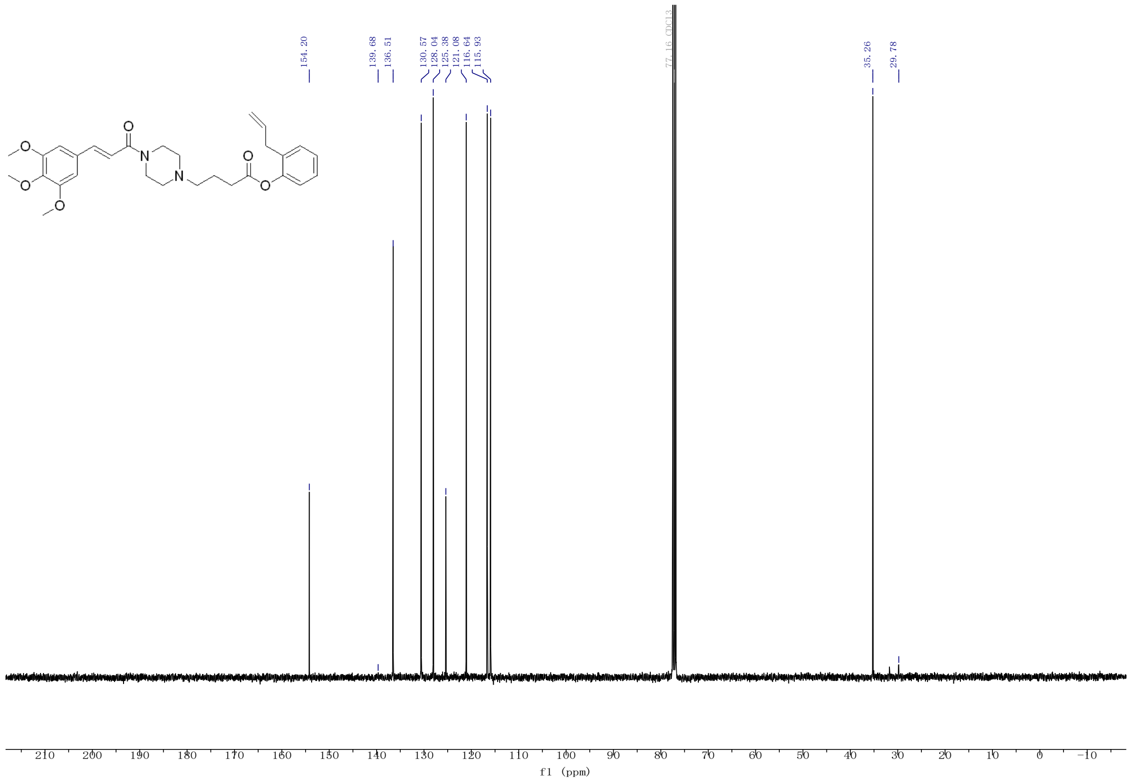

^{13}C NMR (600 MHz, $CDCl_3$) spectrum of **A4**

*2-allylphenyl (E)-4-oxo-4-(4-(3-(3,4,5-trimethoxyphenyl)acryloyl)piperazin-1-yl)butanoate (**B4**):*

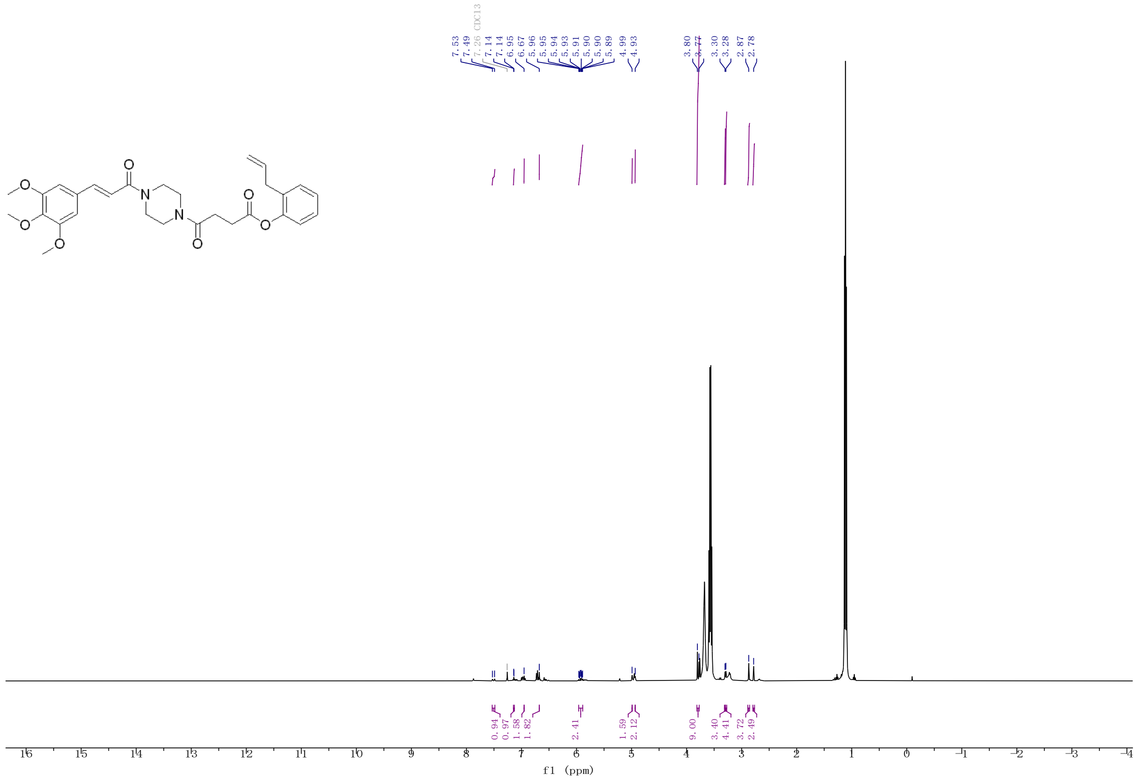

^{1}H NMR (600 MHz, CDCl$_3$) spectrum of **B4**

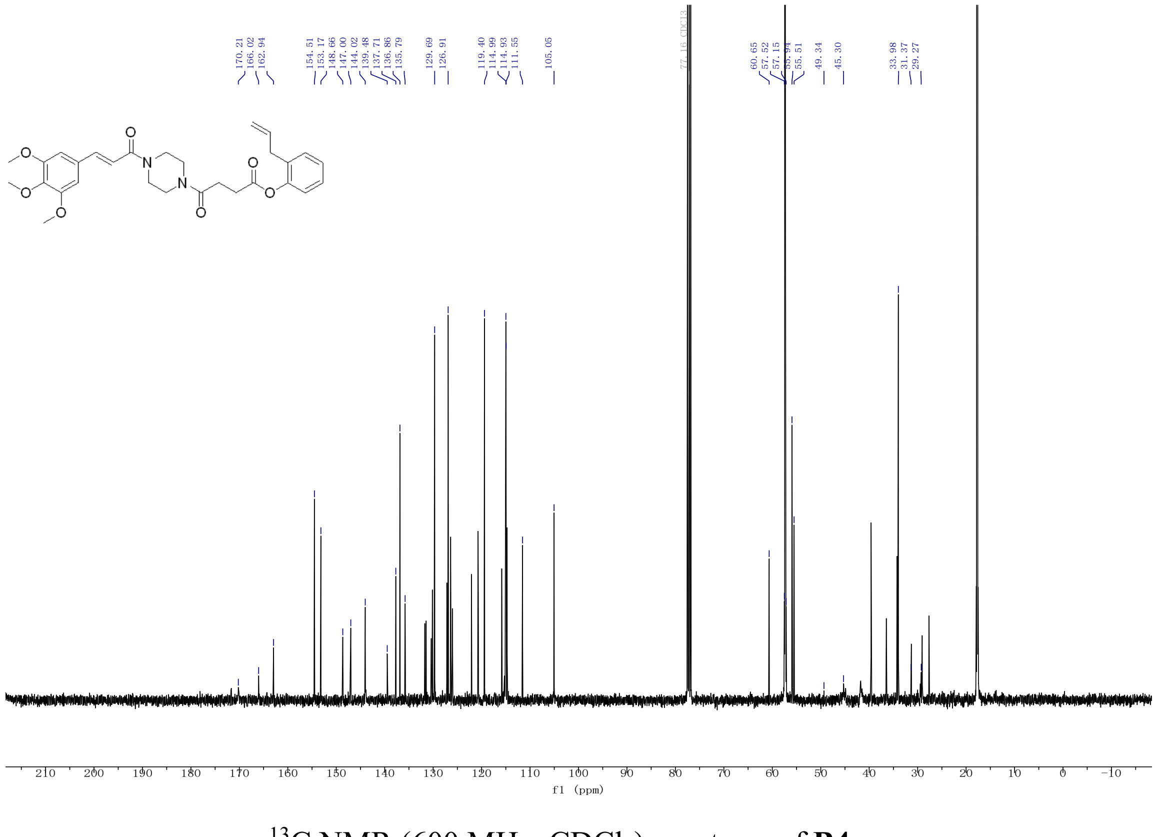

^{13}C NMR (600 MHz, CDCl$_3$) spectrum of **B4**

*(E)-3-(2,4,5-trimethoxyphenyl)allyl 4-(4-((E)-3-(3,4,5-trimethoxyphenyl)acryloyl)piperazin-1-yl)butanoate (**A5**):*

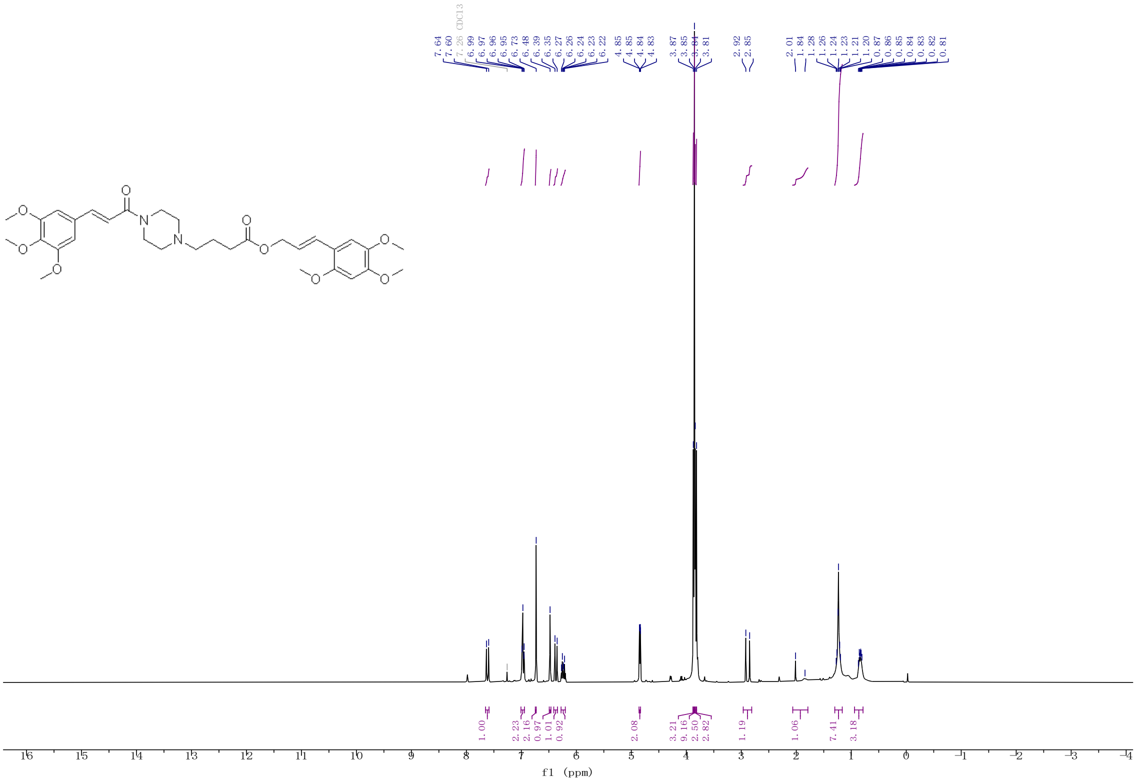

^{1}H NMR (600 MHz, $CDCl_3$) spectrum of **A5**

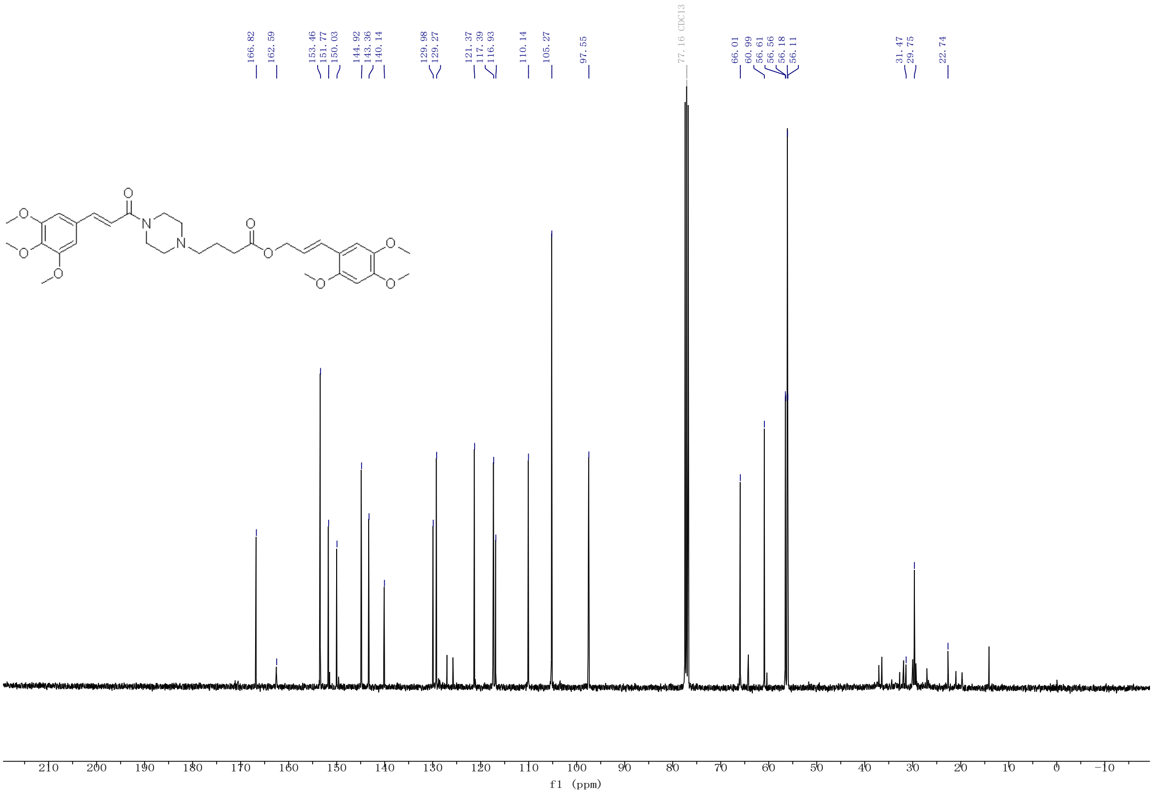

^{13}C NMR (600 MHz, $CDCl_3$) spectrum of **A5**

*(E)-3-(2,4,5-trimethoxyphenyl)allyl 4-oxo-4-(4-((E)-3-(3,4,5-trimethoxyphenyl)acryloyl)piperazin-1-yl)butanoate (**B5**):*

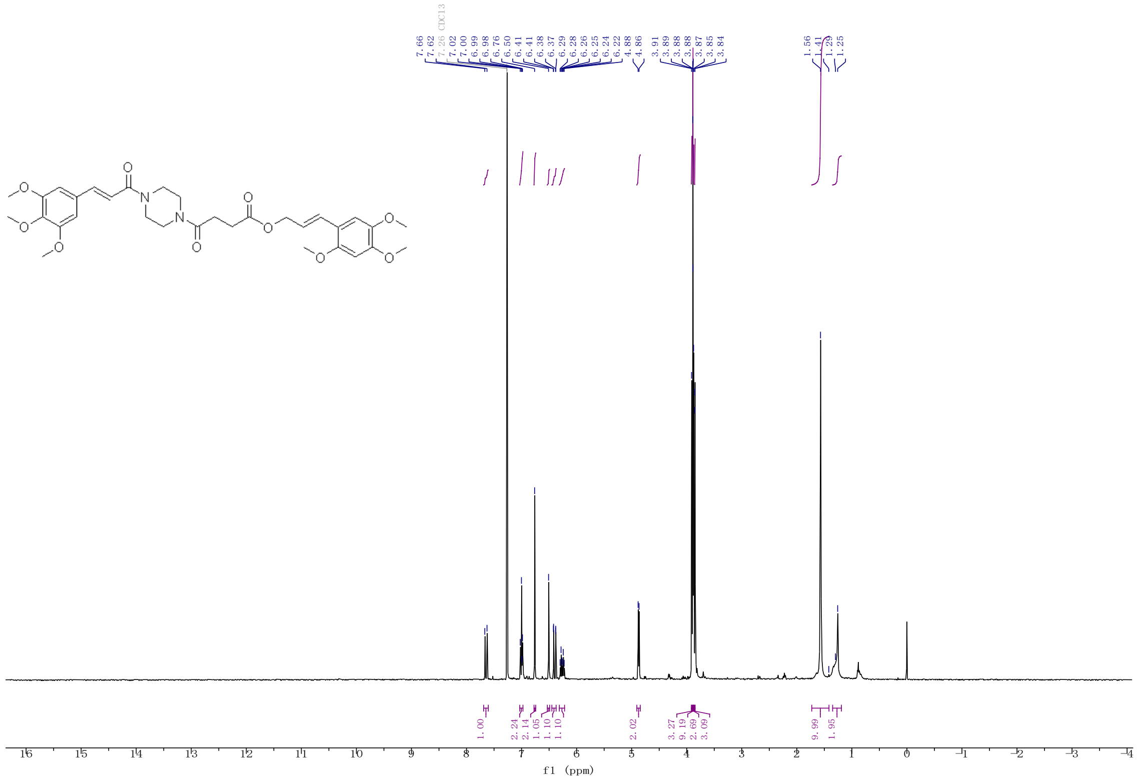

^{1}H NMR (600 MHz, $CDCl_3$) spectrum of **B5**

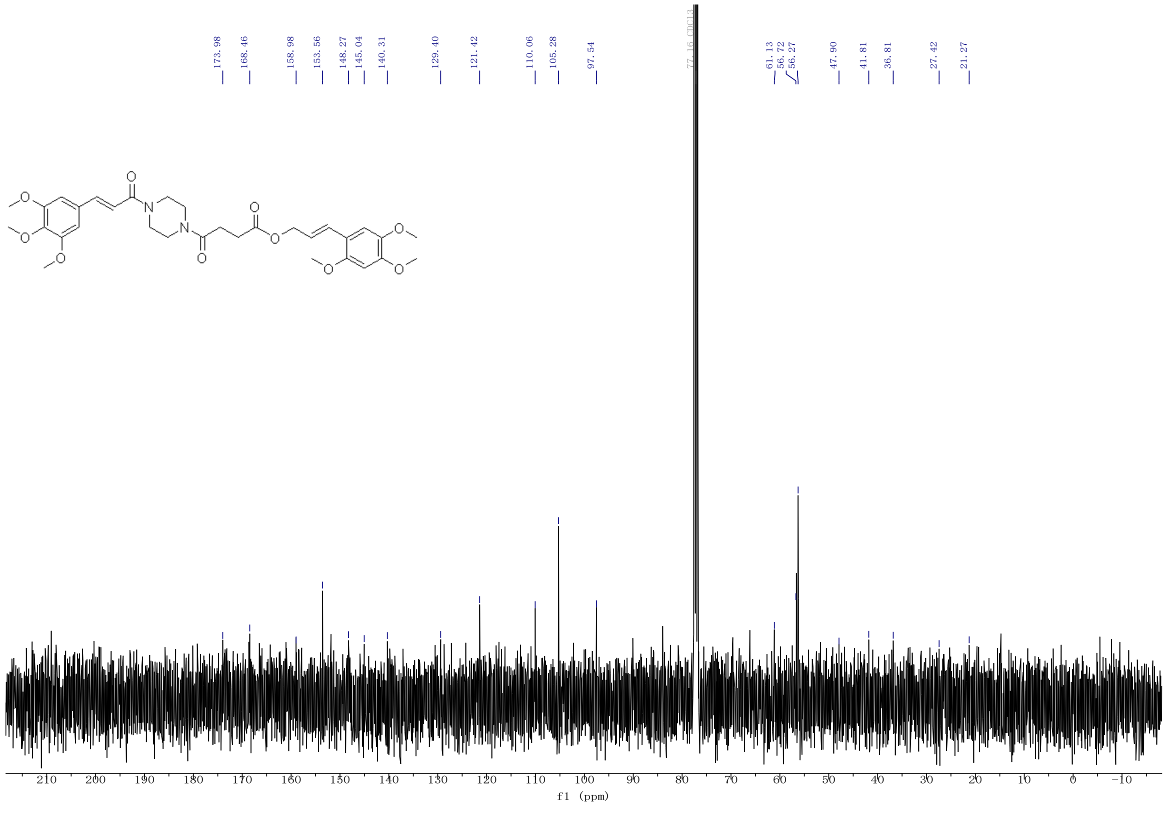

^{13}C NMR (600 MHz, $CDCl_3$) spectrum of **B5**

*cinnamyl 4-(4-((E)-3-(3,4,5-trimethoxyphenyl)acryloyl)piperazin-1-yl)butanoate (**A6**):*

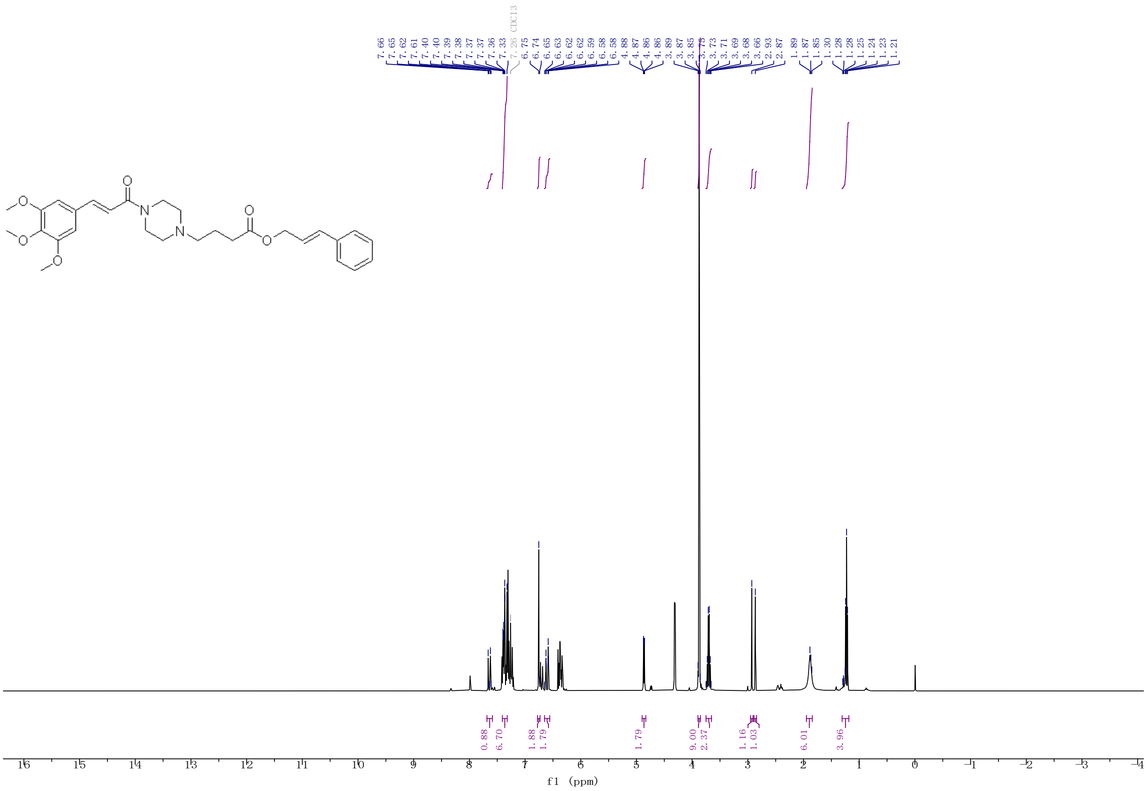

^{1}H NMR (600 MHz, CDCl3) spectrum of **A6**

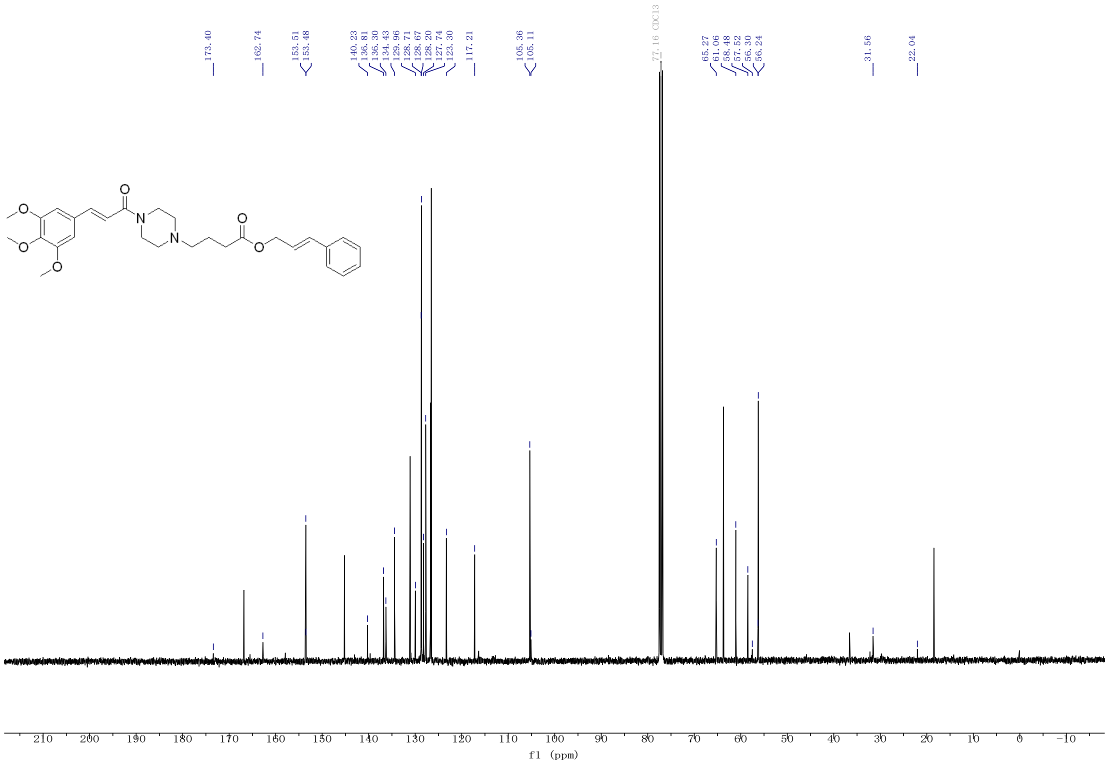

^{13}C NMR (600 MHz, CDCl3) spectrum of **A6**

*cinnamyl 4-oxo-4-(4-((E)-3-(3,4,5-trimethoxyphenyl)acryloyl)piperazin-1-yl)butanoate (**B6**):*

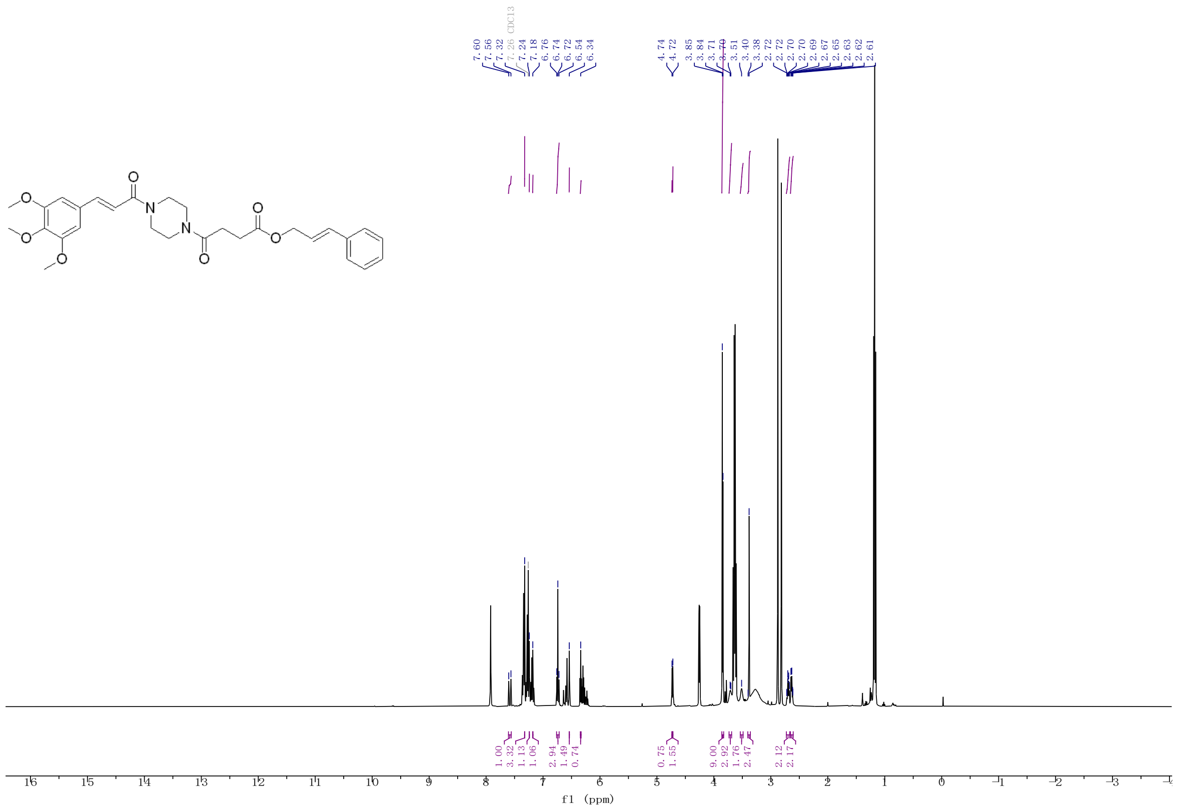

^{1}H NMR (400 MHz, $CDCl_3$) spectrum of **B6**

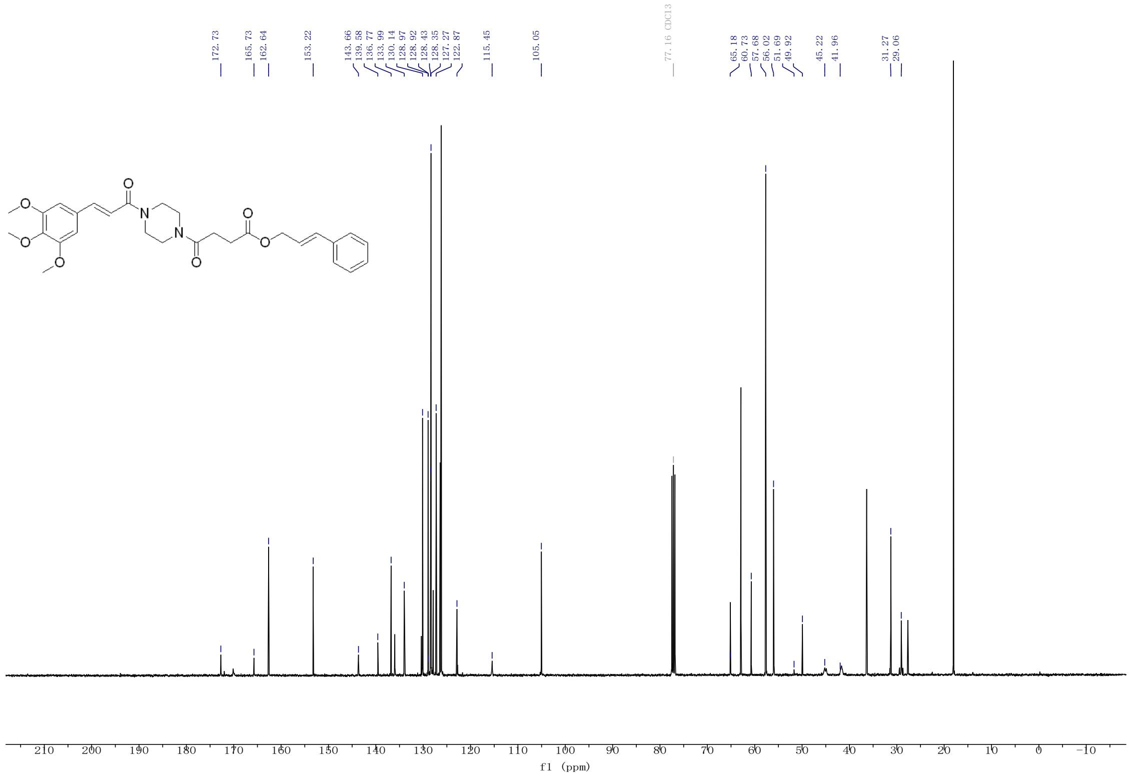

^{13}C NMR (101 MHz, $CDCl_3$) spectrum of **B6**

*rel-(1R,2S,4R)-1,7,7-trimethylbicyclo[2.2.1]heptan-2-yl 4-(4-((E)-3-(3,4,5-trimethoxyphenyl)acryloyl)piperazin-1-yl)butanoate (**A7**):*

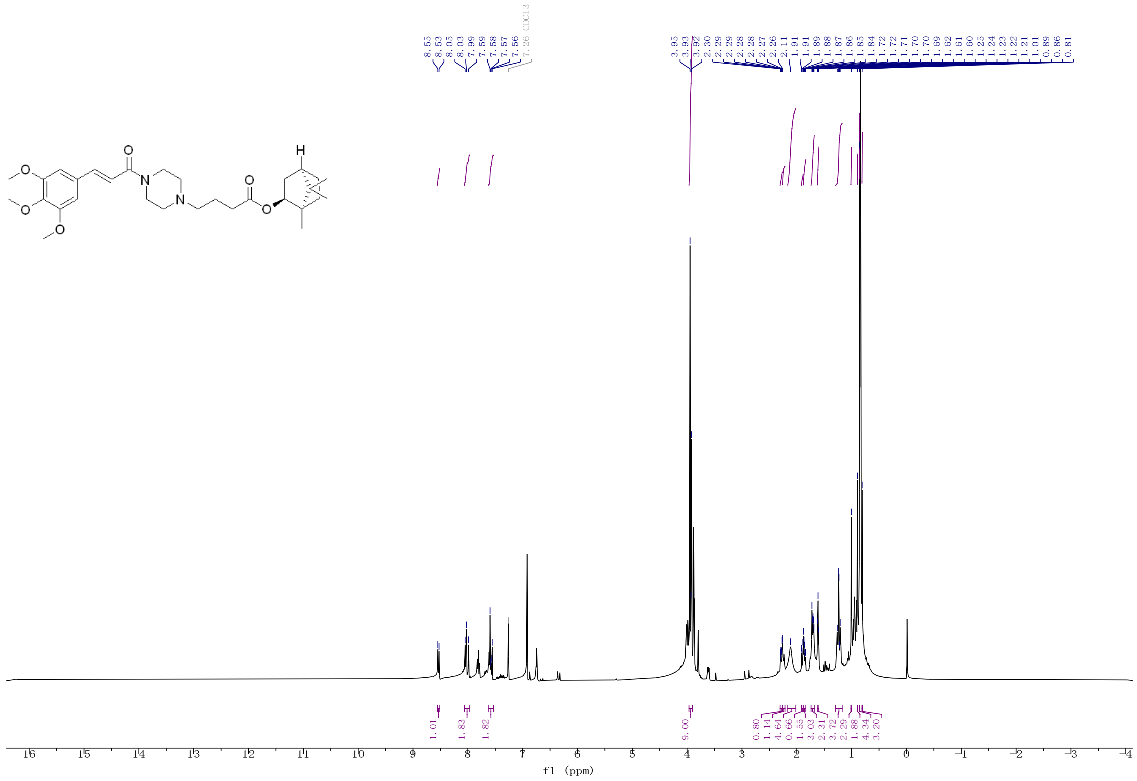

^{1}H NMR (400 MHz, $CDCl_3$) spectrum of **A7**

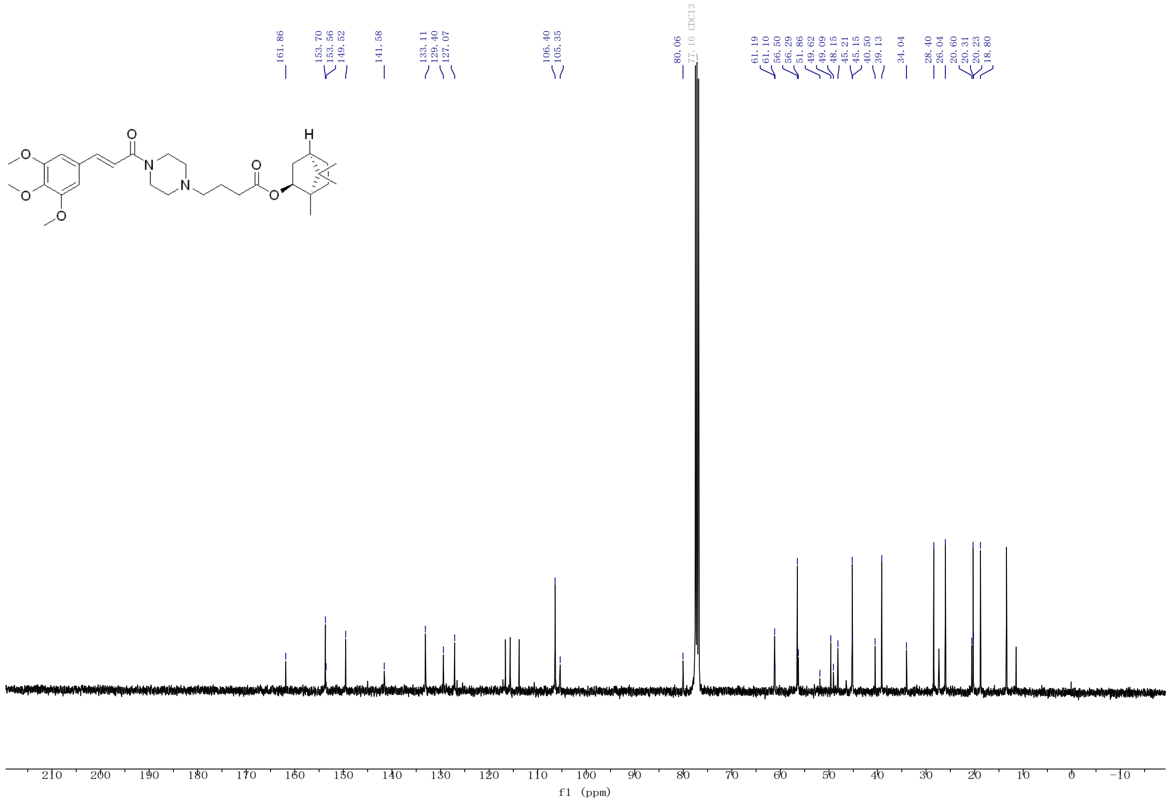

^{13}C NMR (101 MHz, $CDCl_3$) spectrum of **A7**

*(1R,2S,4R)-1,7,7-trimethylbicyclo[2.2.1]heptan-2-yl 4-oxo-4-(4-((E)-3-(3,4,5-trimethoxyphenyl)acryloyl)piperazin-1-yl)butanoate (**B**7):*

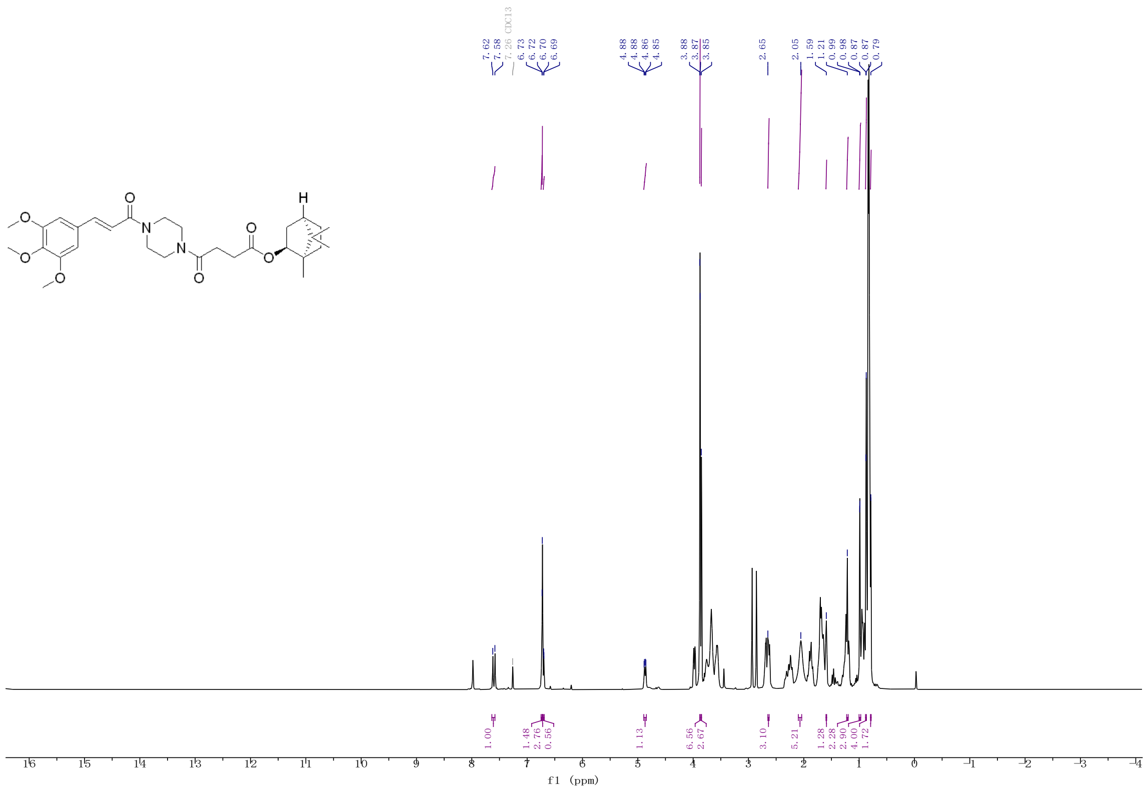

^{1}H NMR (400 MHz, $CDCl_3$) spectrum of **B7**

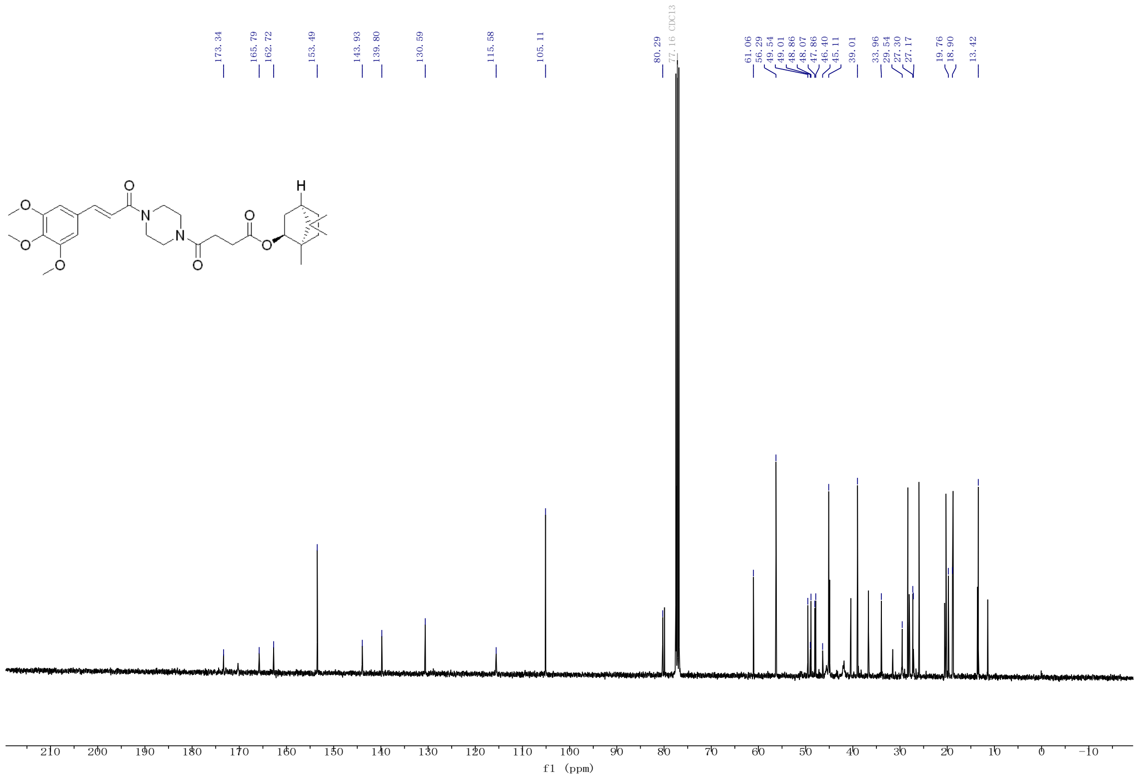

^{13}C NMR (101 MHz, $CDCl_3$) spectrum of **B7**

*(E)-1-(4-benzhydrylpiperazin-1-yl)-4-(4-(3-(3,4,5-trimethoxyphenyl)acryloyl)piperazin-1-yl)butan-1-one (**A8**):*

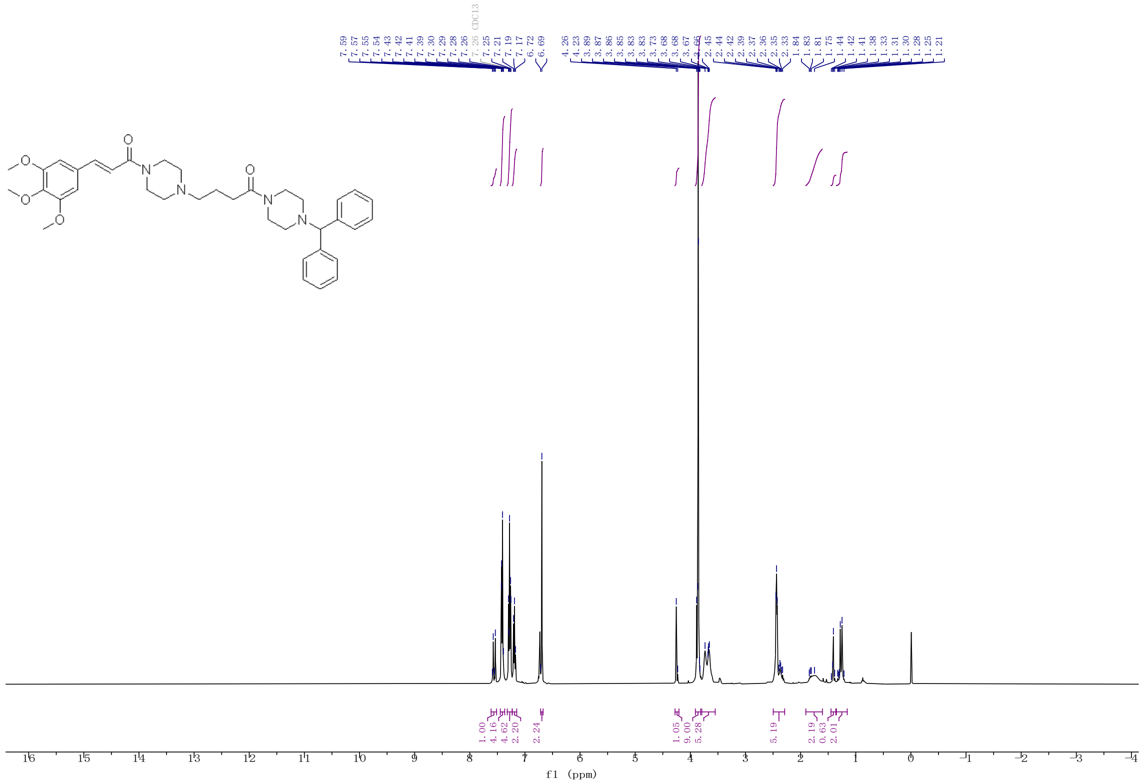

^{1}H NMR (400 MHz, CDCl$_3$) spectrum of **A8**

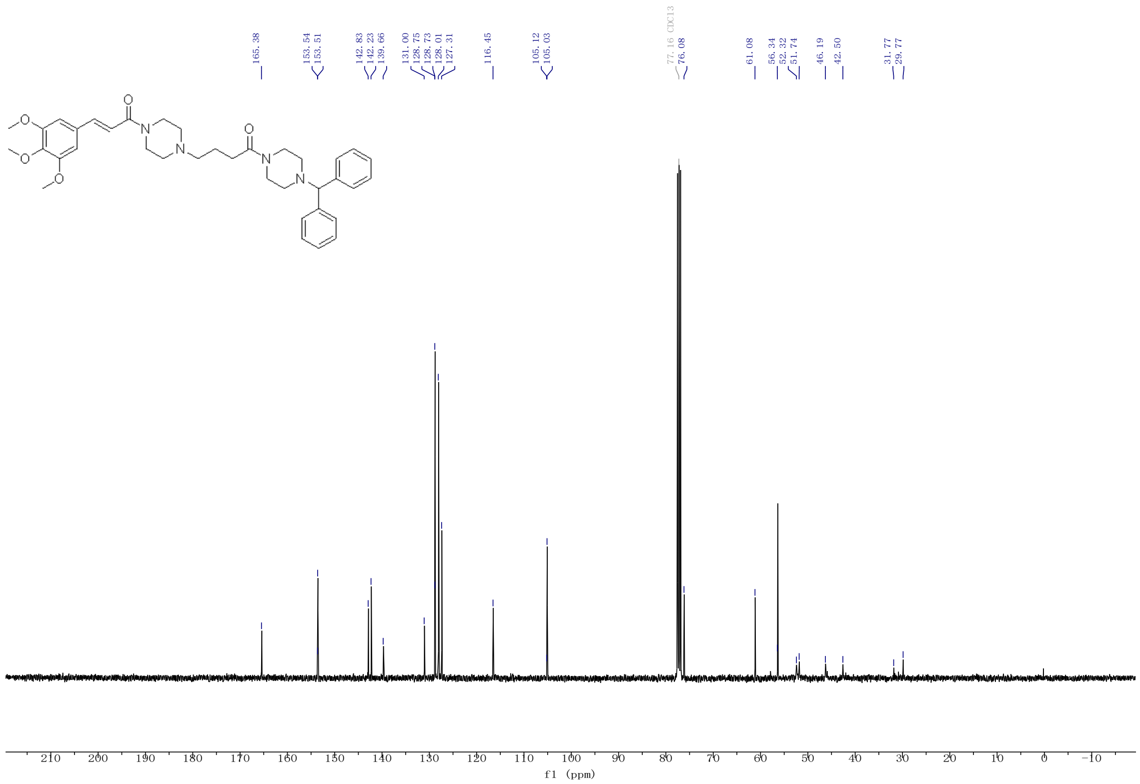

^{13}C NMR (101 MHz, CDCl$_3$) spectrum of **A8**

*(E)-1-(4-benzhydrylpiperazin-1-yl)-4-(4-(3-(3,4,5-trimethoxyphenyl)acryloyl)piperazin-1-yl)butane-1,4-dione (**B8**):*

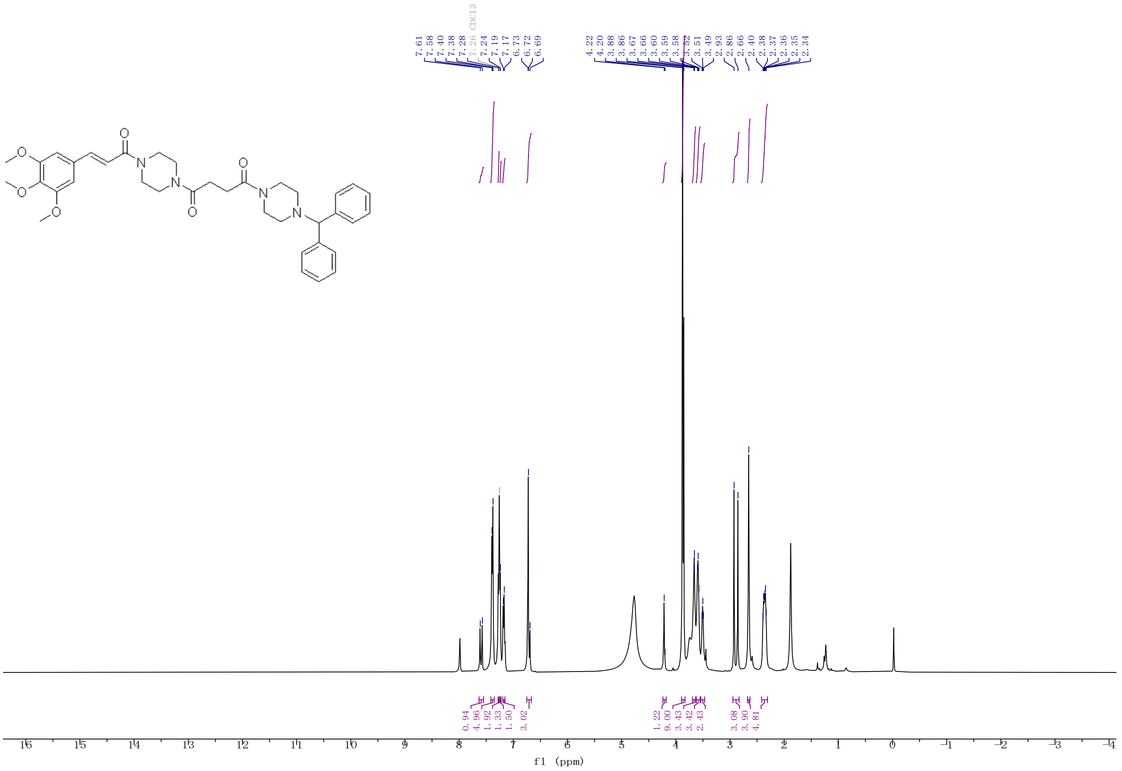

^{1}H NMR (400 MHz, CDCl$_3$) spectrum of **B8**

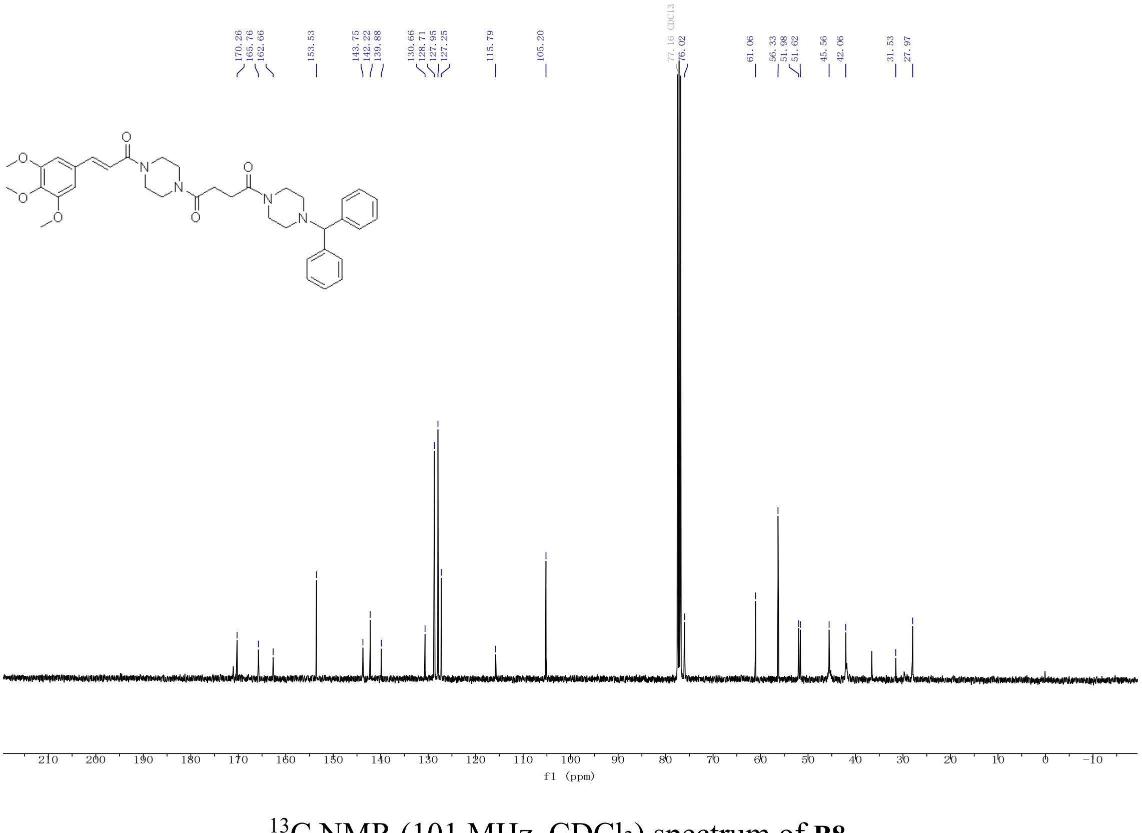

^{13}C NMR (101 MHz, CDCl$_3$) spectrum of **B8**

*(E)-1-(4-(bis(4-fluorophenyl)methyl)piperazin-1-yl)-4-(4-(3-(3,4,5-trimethoxyphenyl)acryloyl)piperazin-1-yl)butan-1-one (**A9**):*

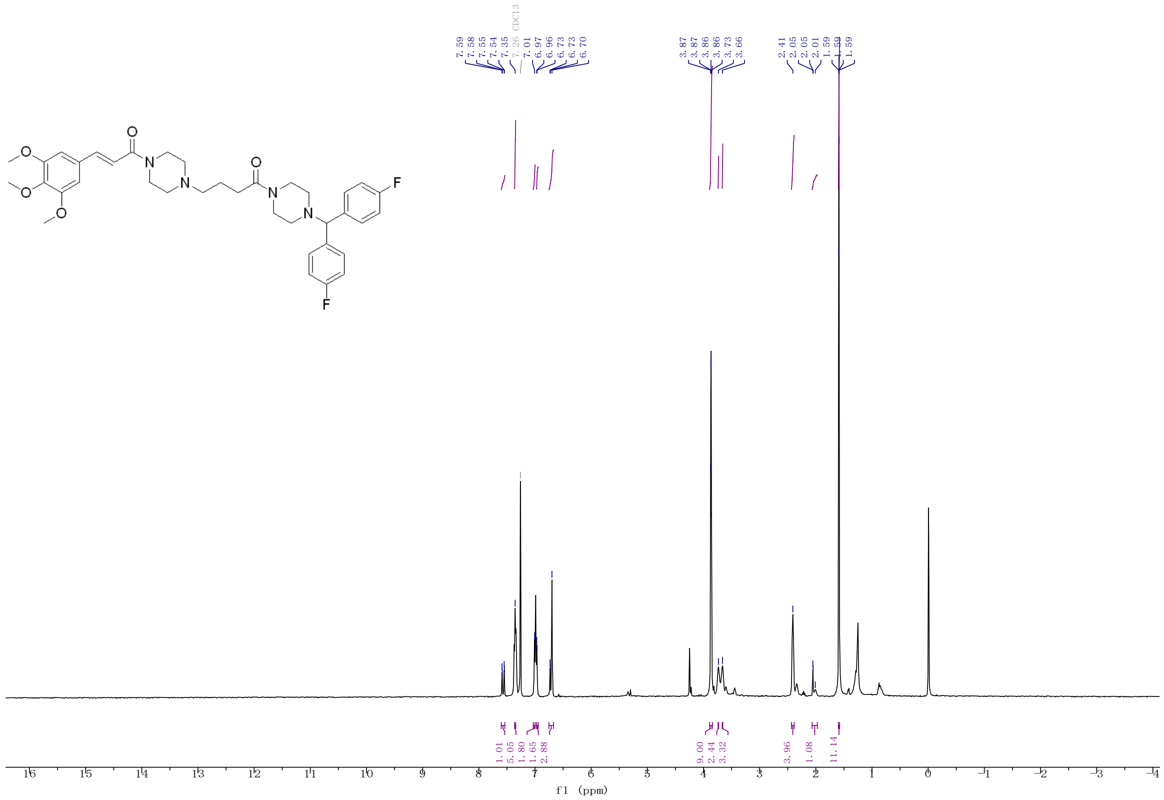

^{1}H NMR (400 MHz, $CDCl_3$) spectrum of **A9**

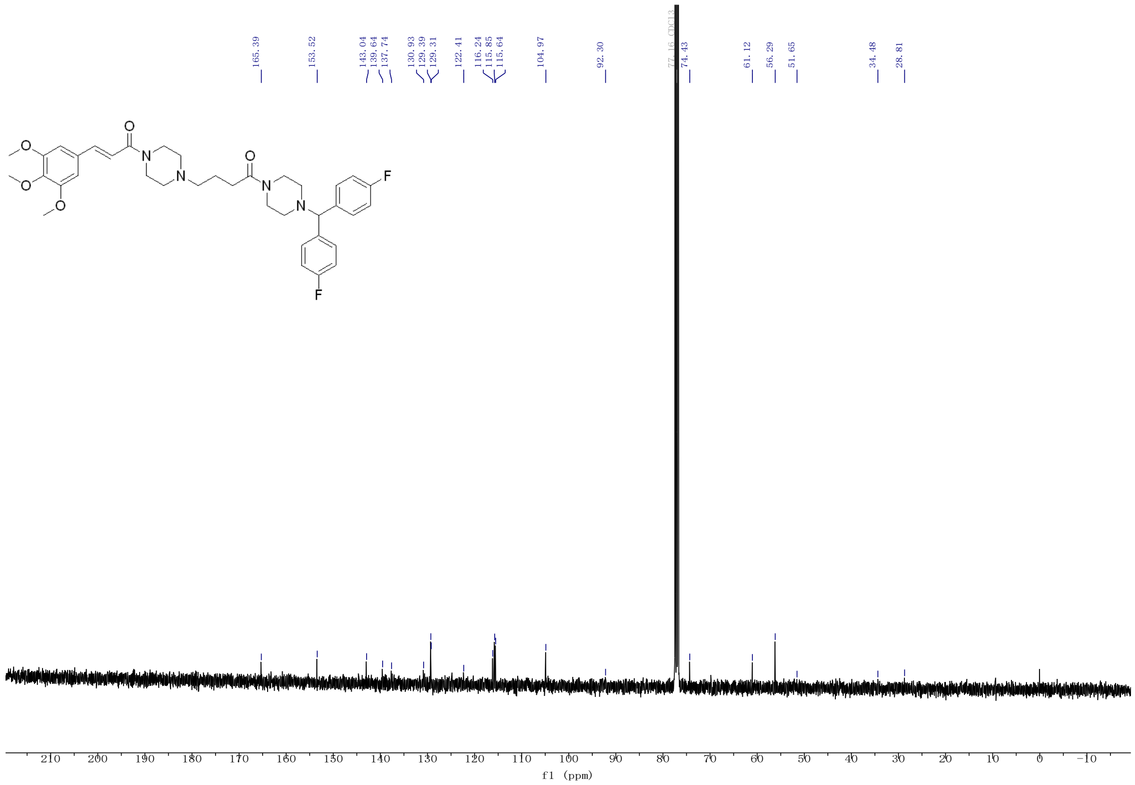

^{13}C NMR (101 MHz, $CDCl_3$) spectrum of **A9**

*(E)-1-(4-(bis(4-fluorophenyl)methyl)piperazin-1-yl)-4-(4-(3-(3,4,5-trimethoxyphenyl)acryloyl)piperazin-1-yl)butane-1,4-dione (**B9**):*

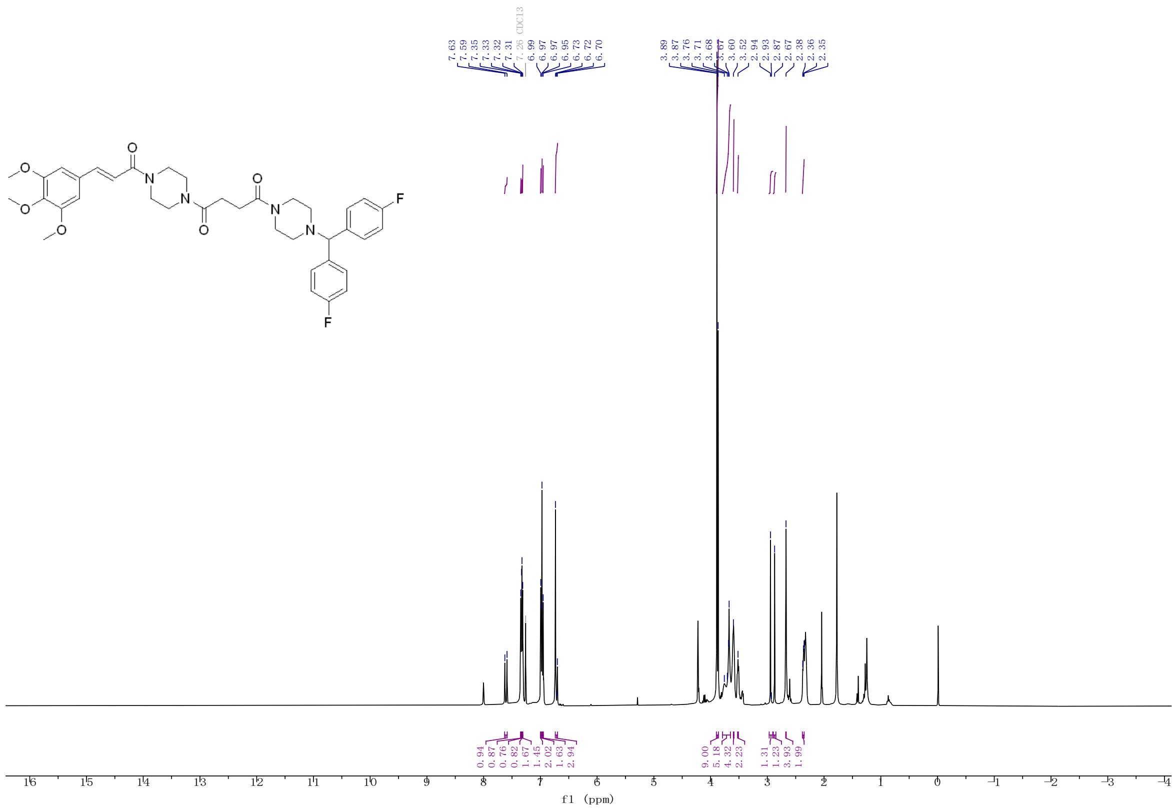

^{1}H NMR (400 MHz, $CDCl_3$) spectrum of **B9**

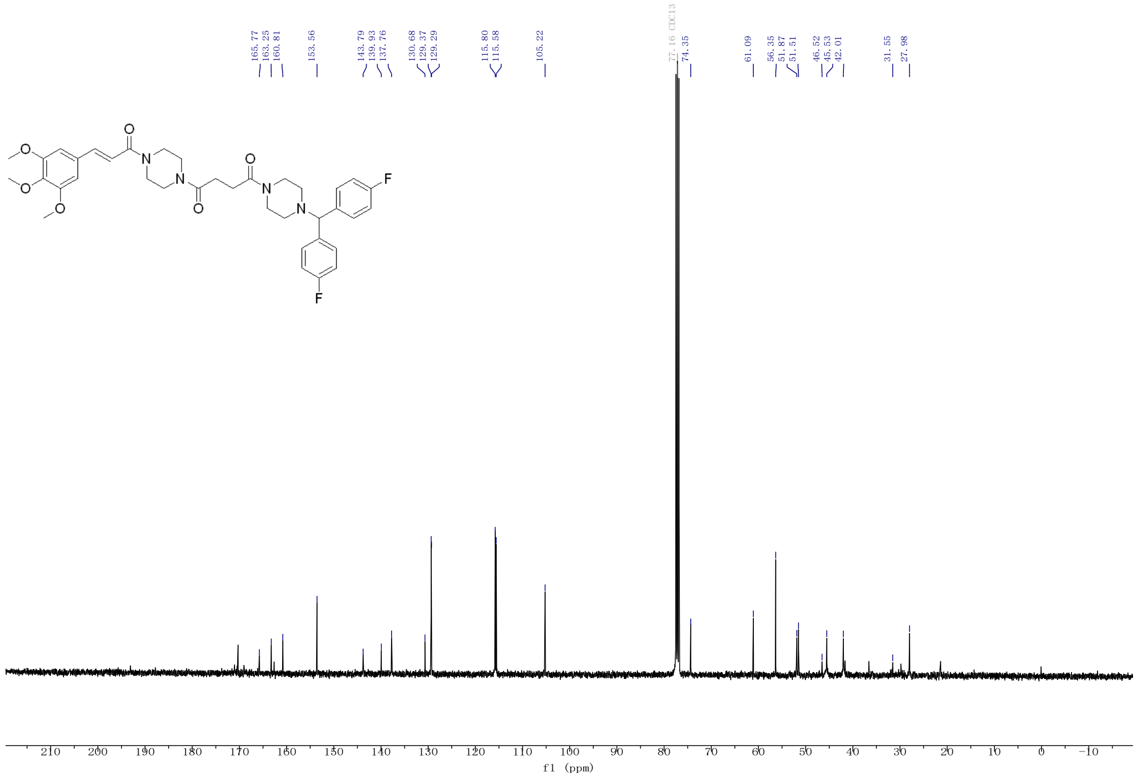

^{13}C NMR (101 MHz, $CDCl_3$) spectrum of **B9**

*(E)-1-(4-(benzo[d]isothiazol-3-yl)piperazin-1-yl)-4-(4-(3-(3,4,5-trimethoxyphenyl)acryloyl)piperazin-1-yl)butan-1-one (**A10**):*

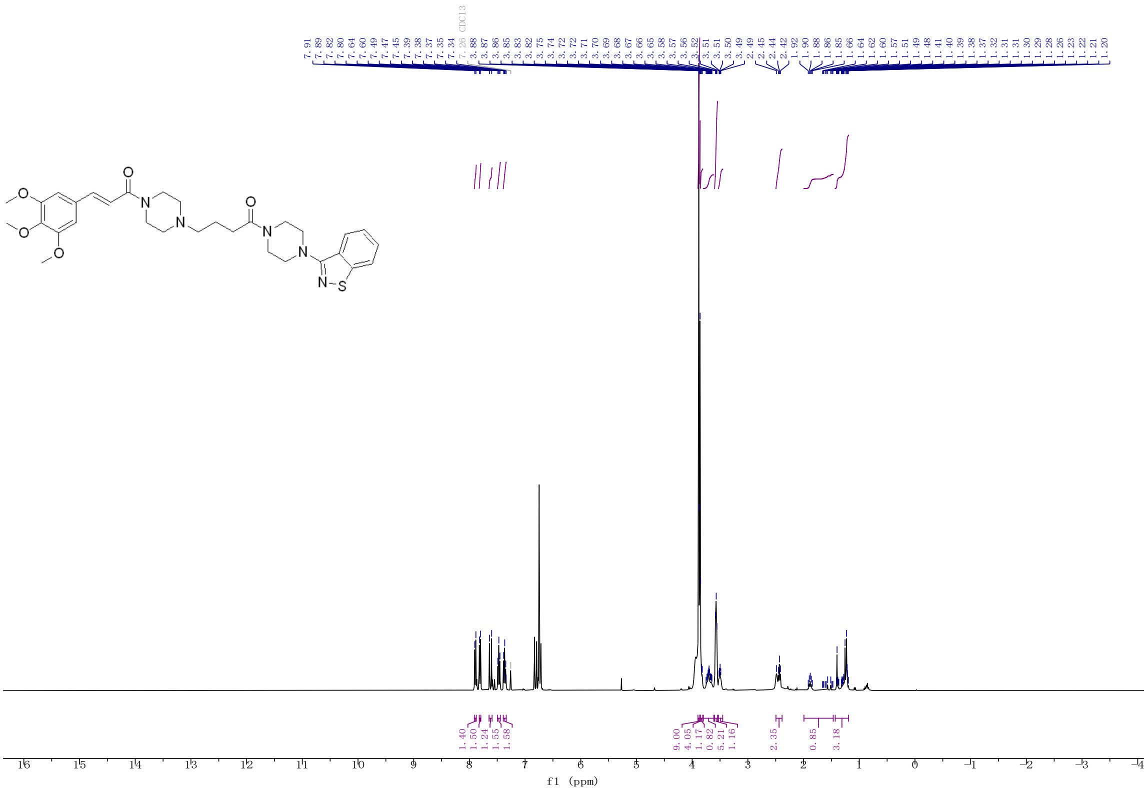

^{1}H NMR (400 MHz, CDCl$_3$) spectrum of **A10**

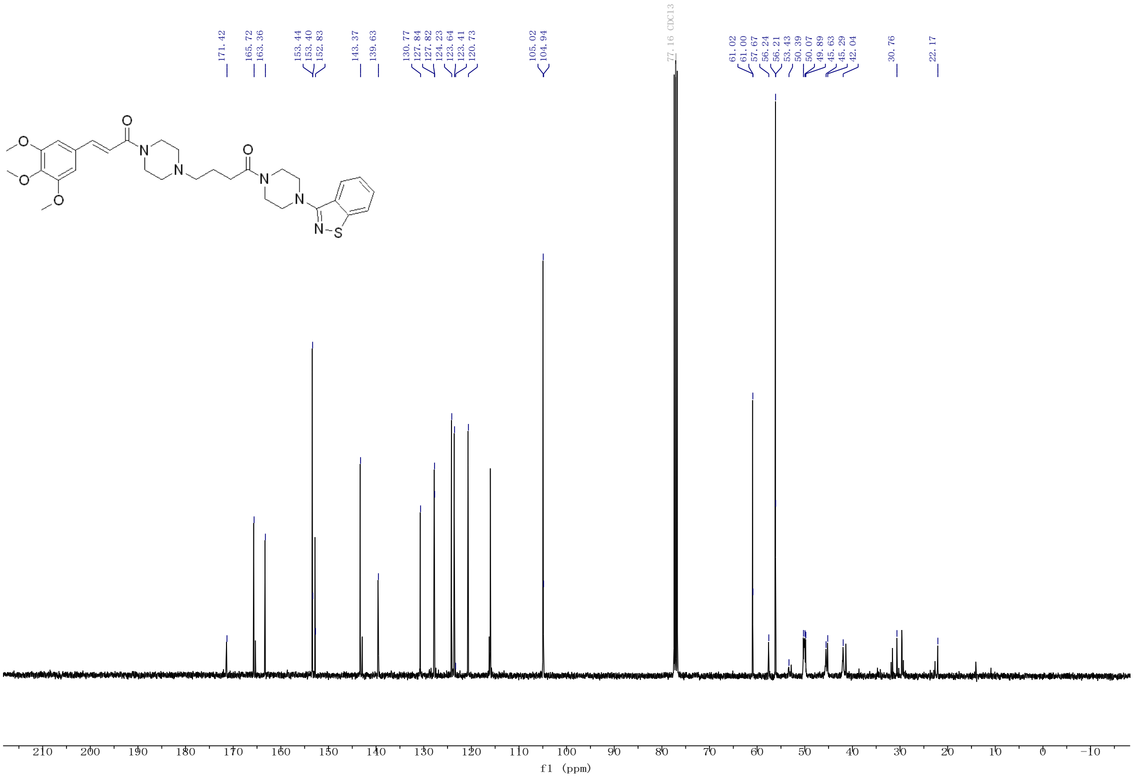

^{13}C NMR (101 MHz, CDCl$_3$) spectrum of **A10**

*(E)-1-(4-(benzo[d]isothiazol-3-yl)piperazin-1-yl)-4-(4-(3-(3,4,5-trimethoxyphenyl)acryloyl)piperazin-1-yl)butane-1,4-dione (**B10**):*

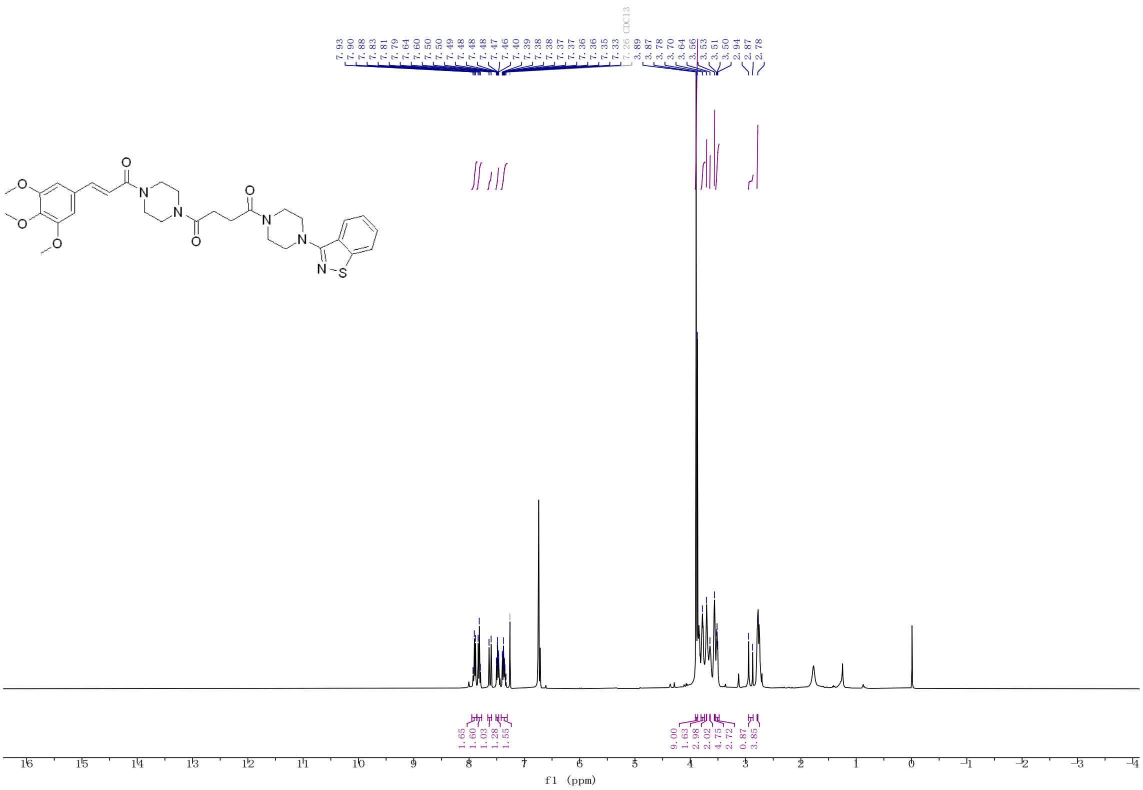

^{1}H NMR (400 MHz, $CDCl_3$) spectrum of **B10**

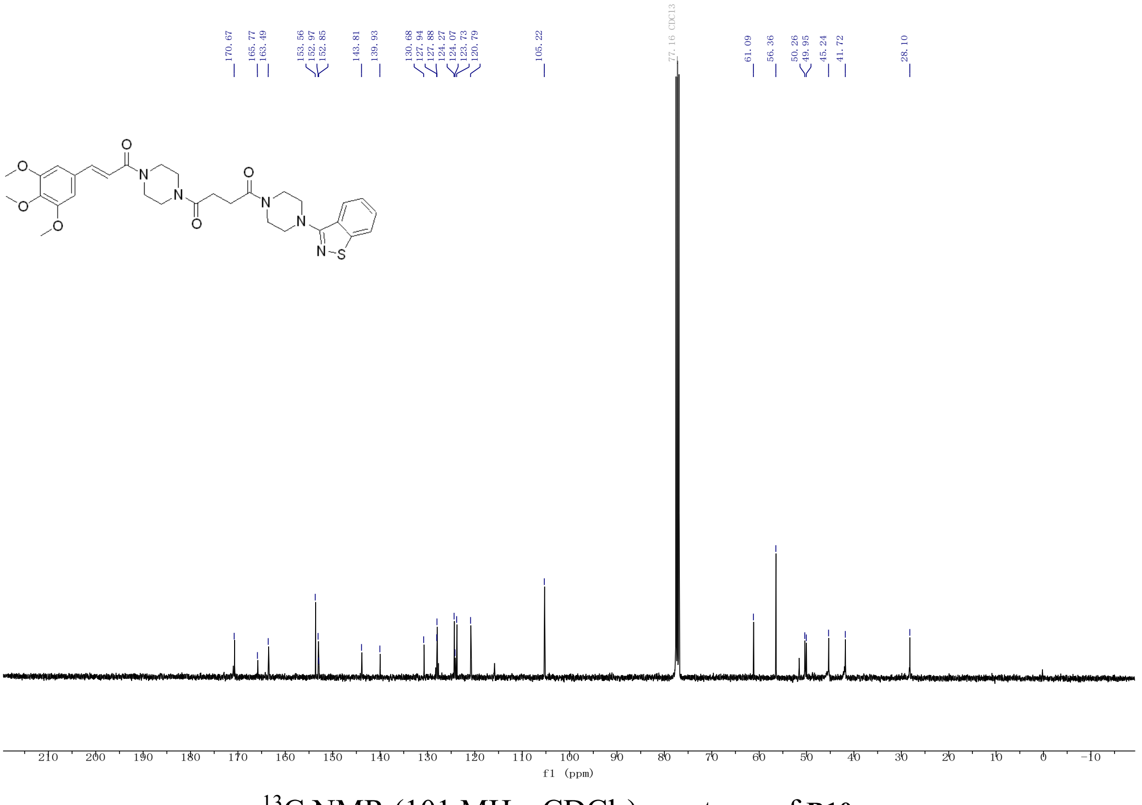

^{13}C NMR (101 MHz, $CDCl_3$) spectrum of **B10**

*(E)-1-(4-phenylpiperazin-1-yl)-4-(4-(3-(3,4,5-trimethoxyphenyl)acryloyl)piperazin-1-yl)butan-1-one (**A11**):*

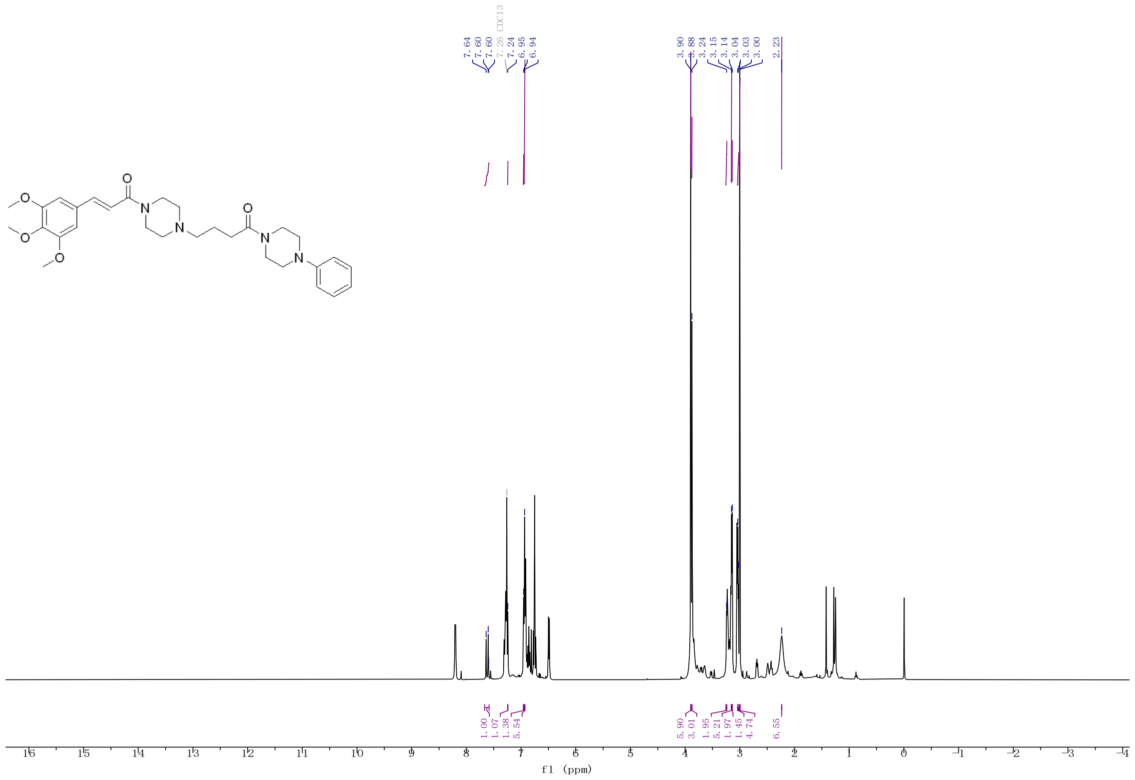

^{1}H NMR (400 MHz, $CDCl_3$) spectrum of **A11**

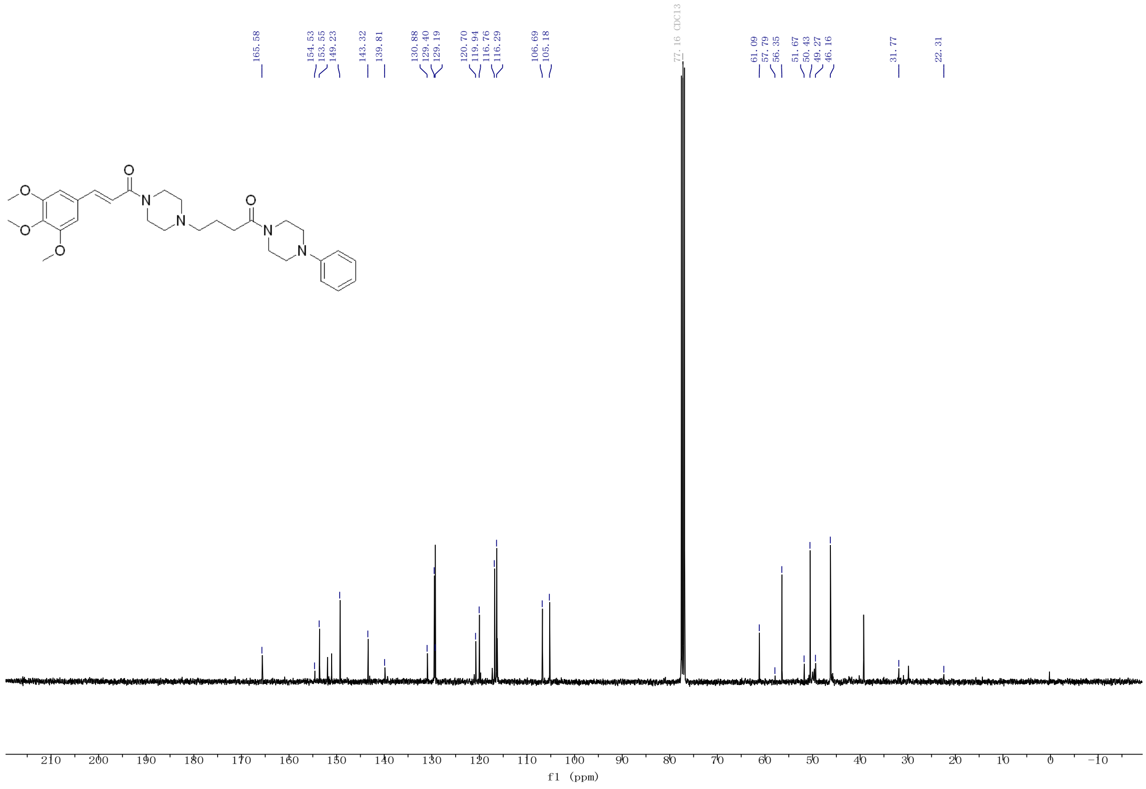

^{13}C NMR (101 MHz, $CDCl_3$) spectrum of **A11**

*(E)-1-(4-phenylpiperazin-1-yl)-4-(4-(3-(3,4,5-trimethoxyphenyl)acryloyl)piperazin-1-yl)butane-1,4-dione (**B11**):*

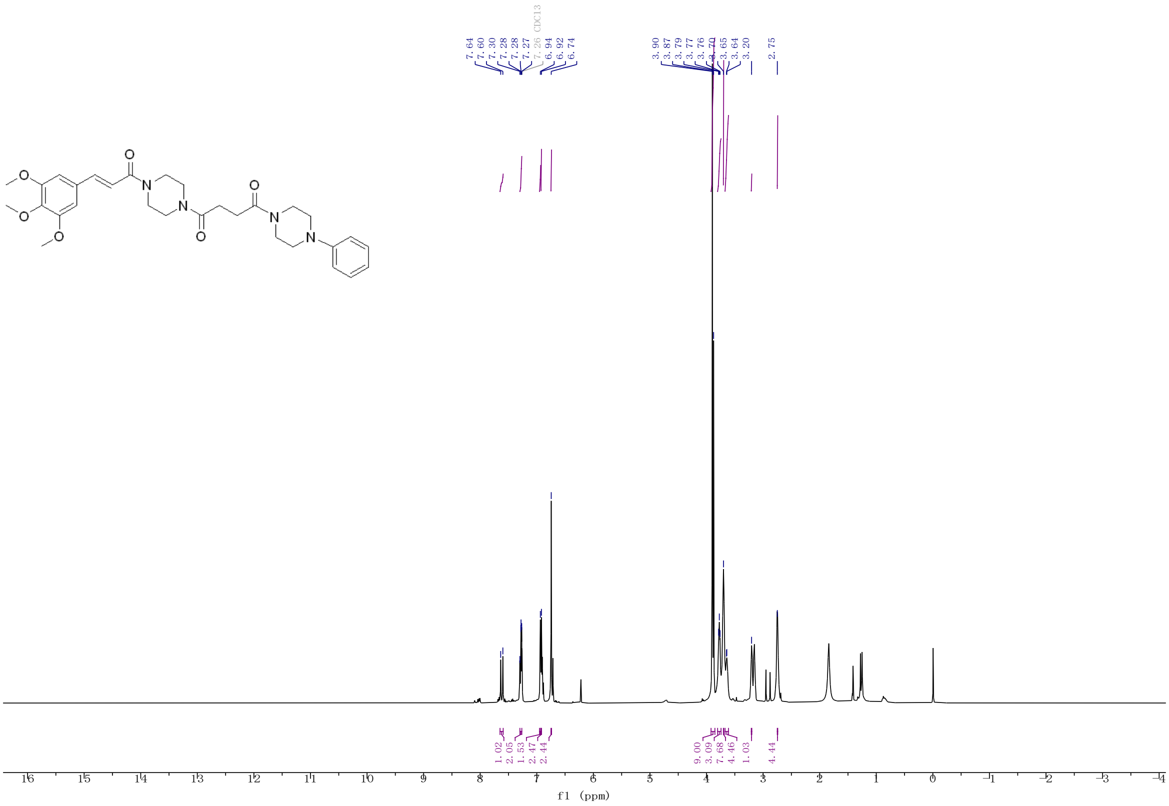

^{1}H NMR (400 MHz, $CDCl_3$) spectrum of **B11**

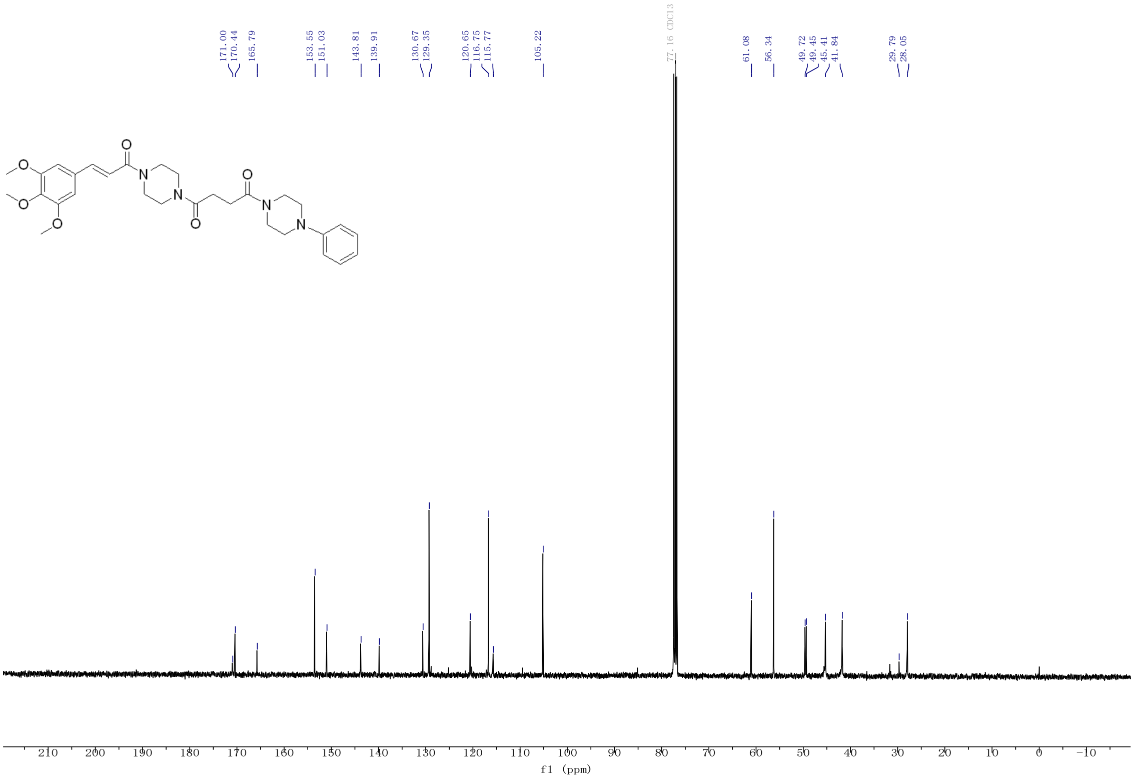

^{13}C NMR (101 MHz, $CDCl_3$) spectrum of **B11**

*(E)-1-(4-(2,3-dichlorophenyl)piperazin-1-yl)-4-(4-(3-(3,4,5-trimethoxyphenyl)acryloyl)piperazin-1-yl)butan-1-one (**A12**):*

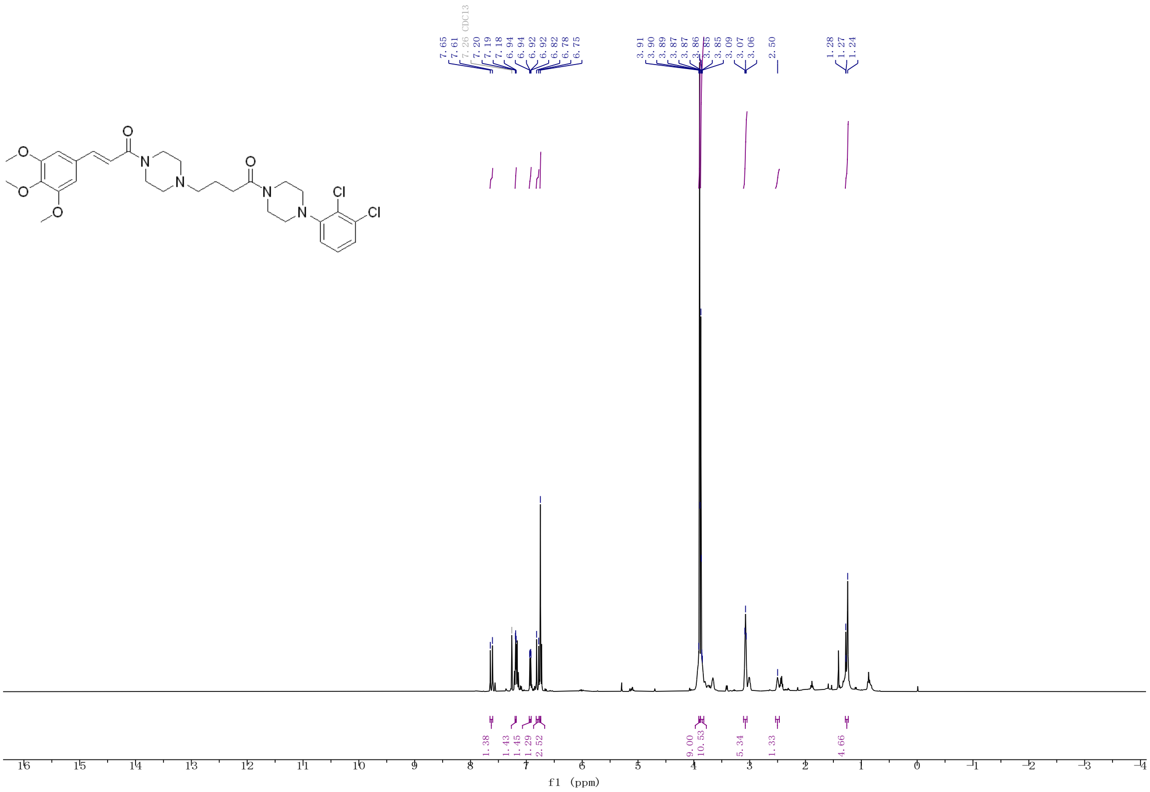

^{1}H NMR (400 MHz, $CDCl_3$) spectrum of **A12**

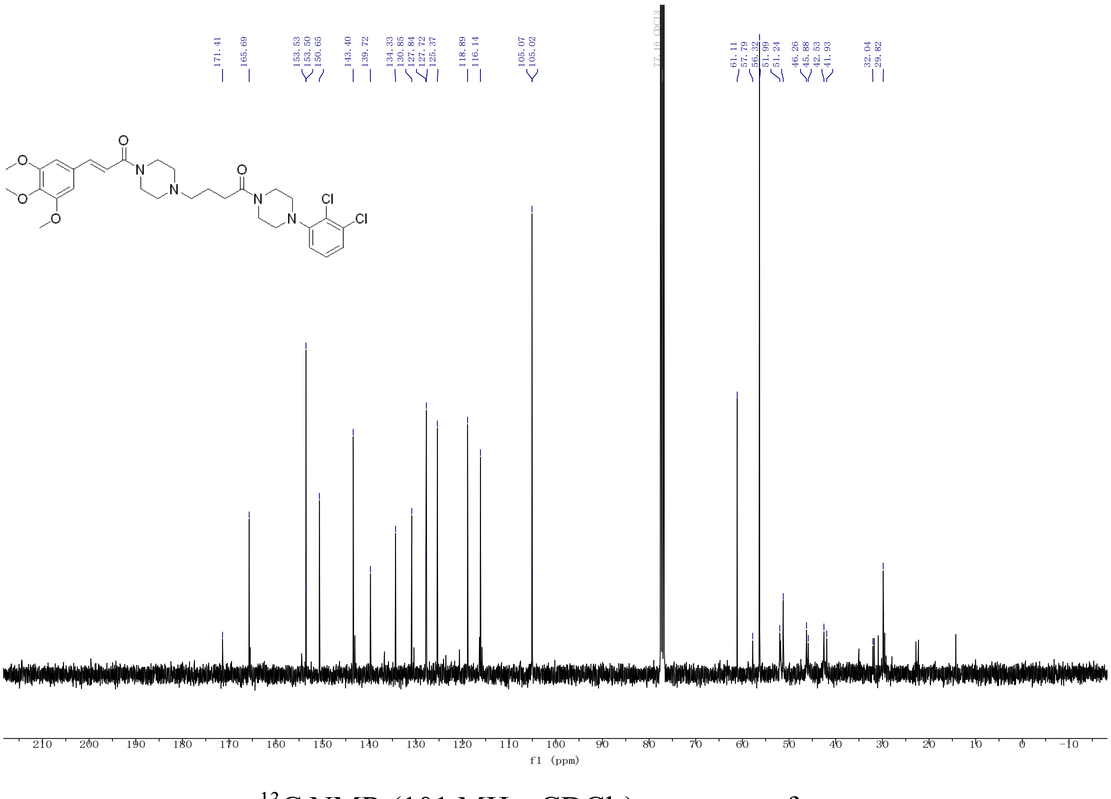

^{13}C NMR (101 MHz, $CDCl_3$) spectrum of **A12**

*(E)-1-(4-(2,3-dichlorophenyl)piperazin-1-yl)-4-(4-(3-(3,4,5-trimethoxyphenyl)acryloyl)piperazin-1-yl)butane-1,4-dione (**B12**):*

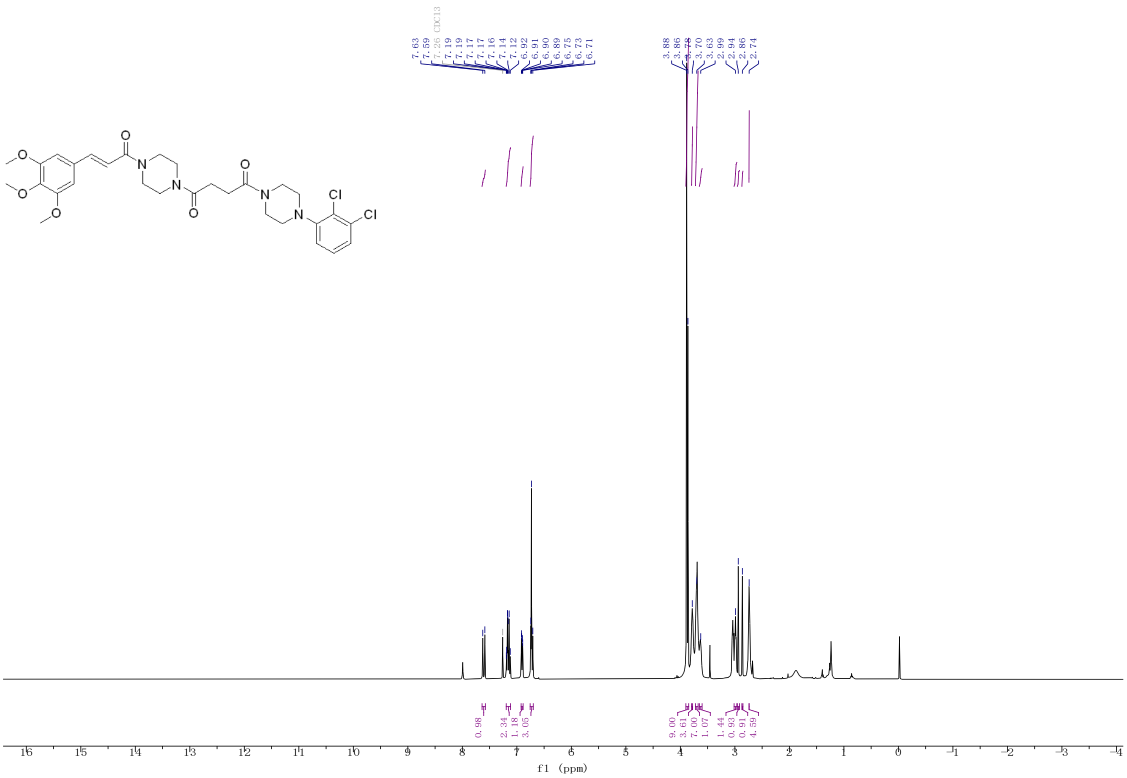

^{1}H NMR (400 MHz, $CDCl_3$) spectrum of **B12**

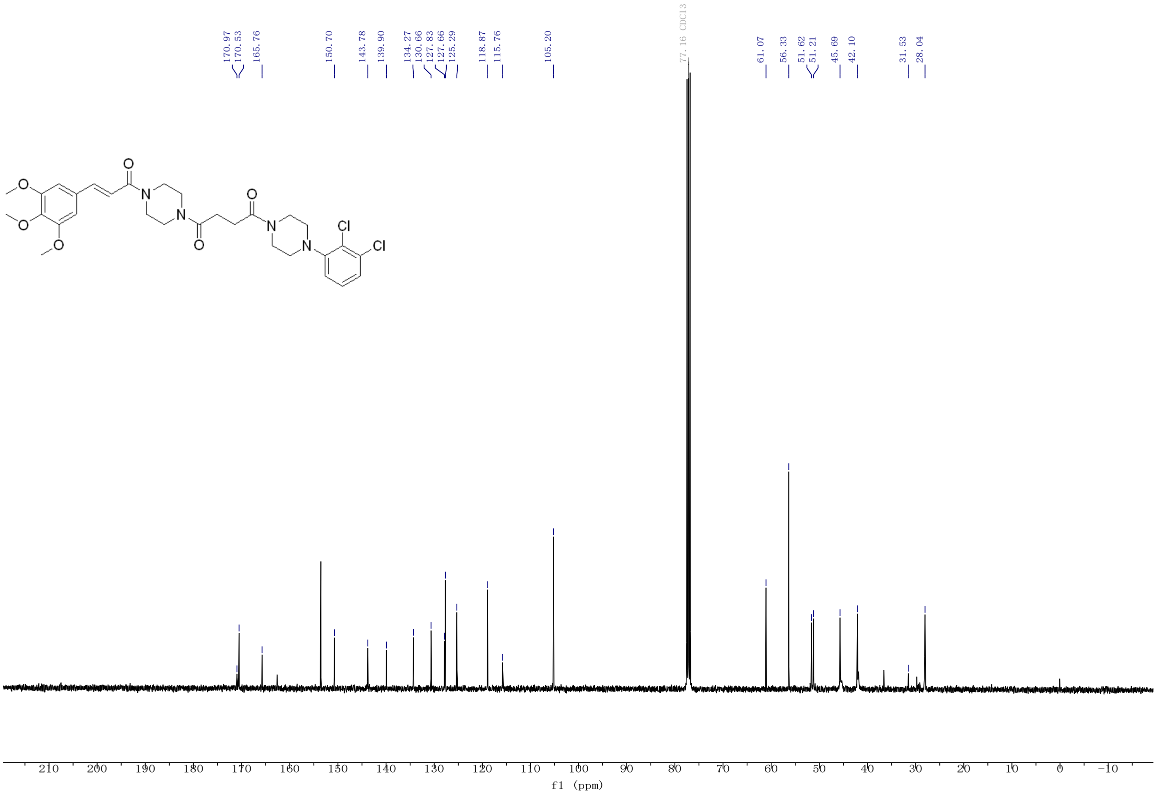

^{13}C NMR (101 MHz, $CDCl_3$) spectrum of **B12**

*(E)-1-(4-(3-chlorophenyl)piperazin-1-yl)-4-(4-(3-(3,4,5-trimethoxyphenyl)acryloyl)piperazin-1-yl)butan-1-one (**A13**):*

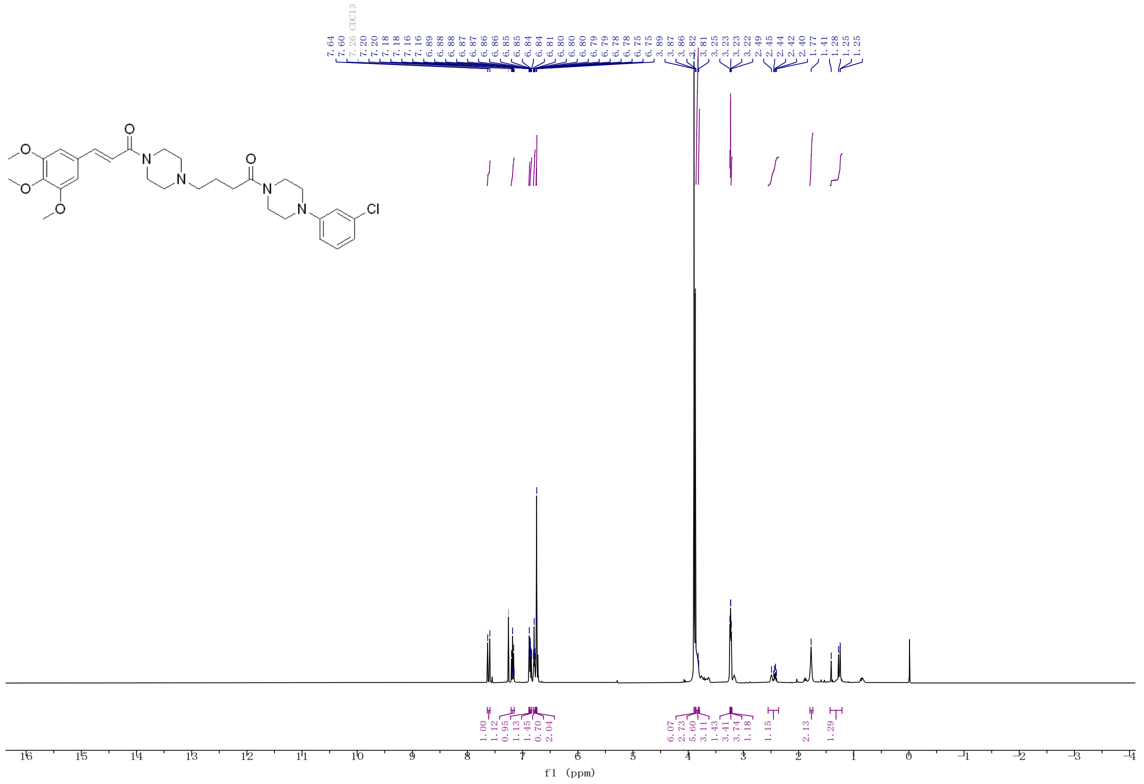

^{1}H NMR (400 MHz, $CDCl_3$) spectrum of **A13**

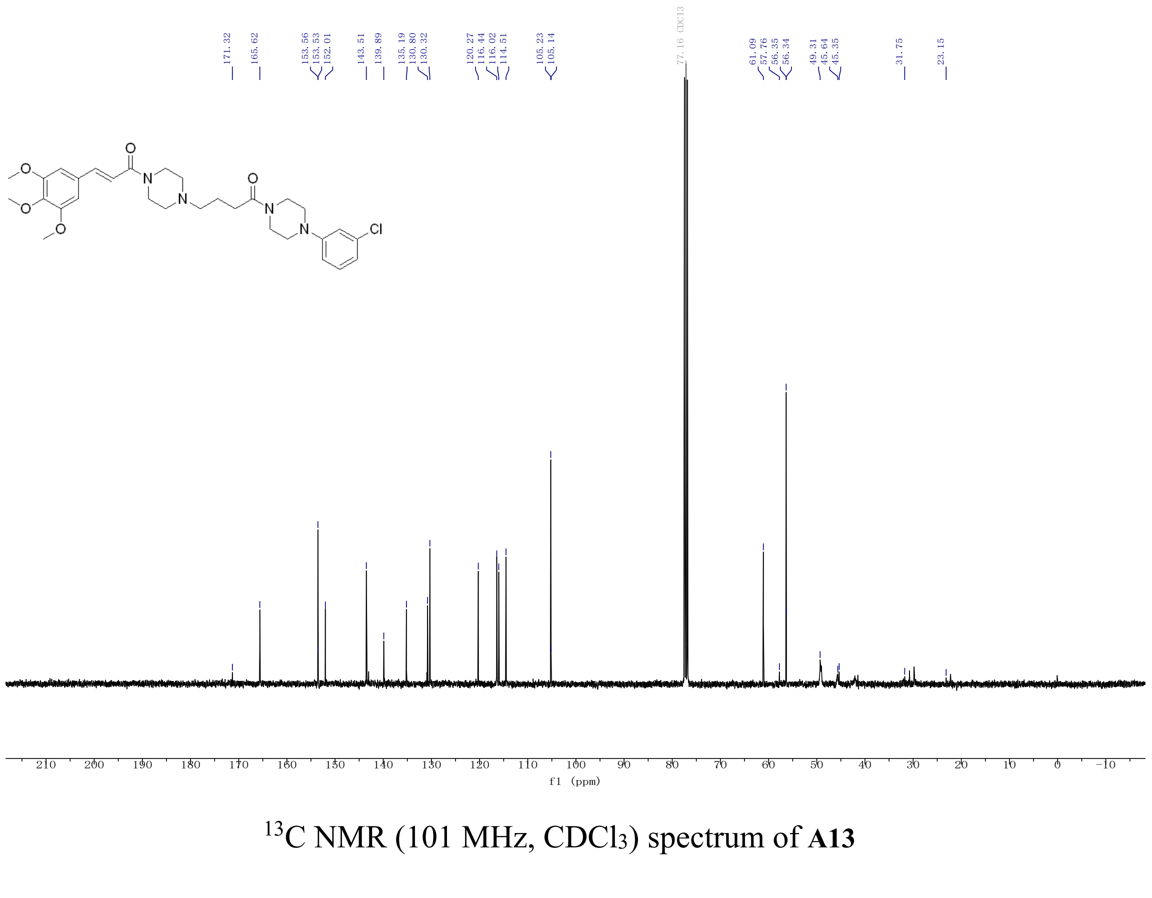

^{13}C NMR (101 MHz, $CDCl_3$) spectrum of **A13**

*(E)-1-(4-(3-chlorophenyl)piperazin-1-yl)-4-(4-(3-(3,4,5-trimethoxyphenyl)acryloyl)piperazin-1-yl)butane-1,4-dione (**B13**):*

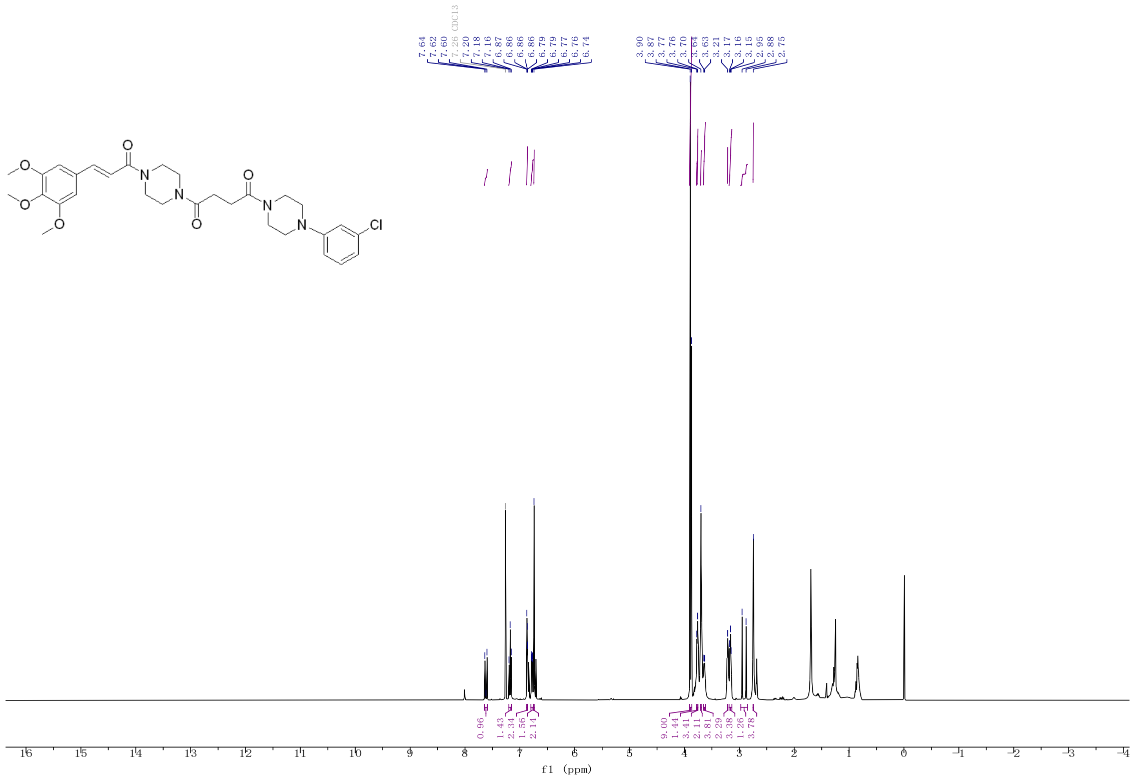

^{1}H NMR (400 MHz, $CDCl_3$) spectrum of **B13**

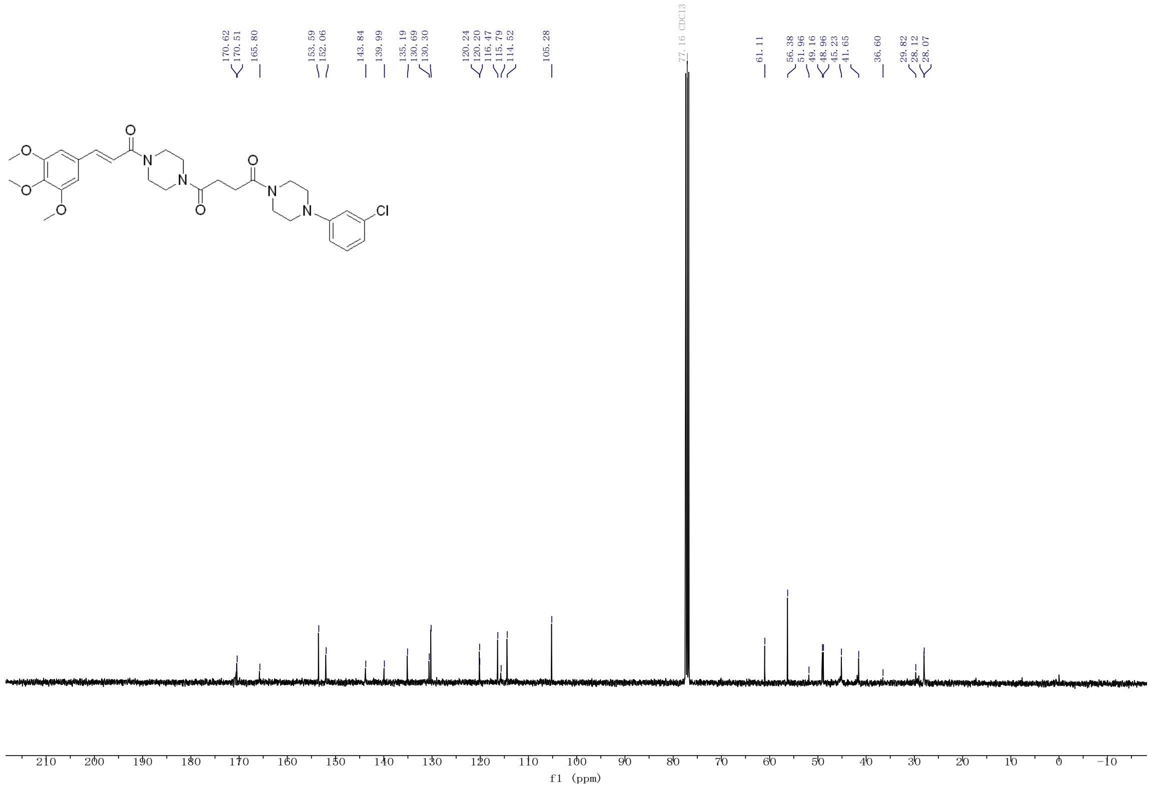

^{13}C NMR (101 MHz, $CDCl_3$) spectrum of **B13**

*(E)-1-(4-(4-(trifluoromethyl)phenyl)piperazin-1-yl)-4-(4-(3-(3,4,5-trimethoxyphenyl)acryloyl)piperazin-1-yl)butan-1-one (**A14**):*

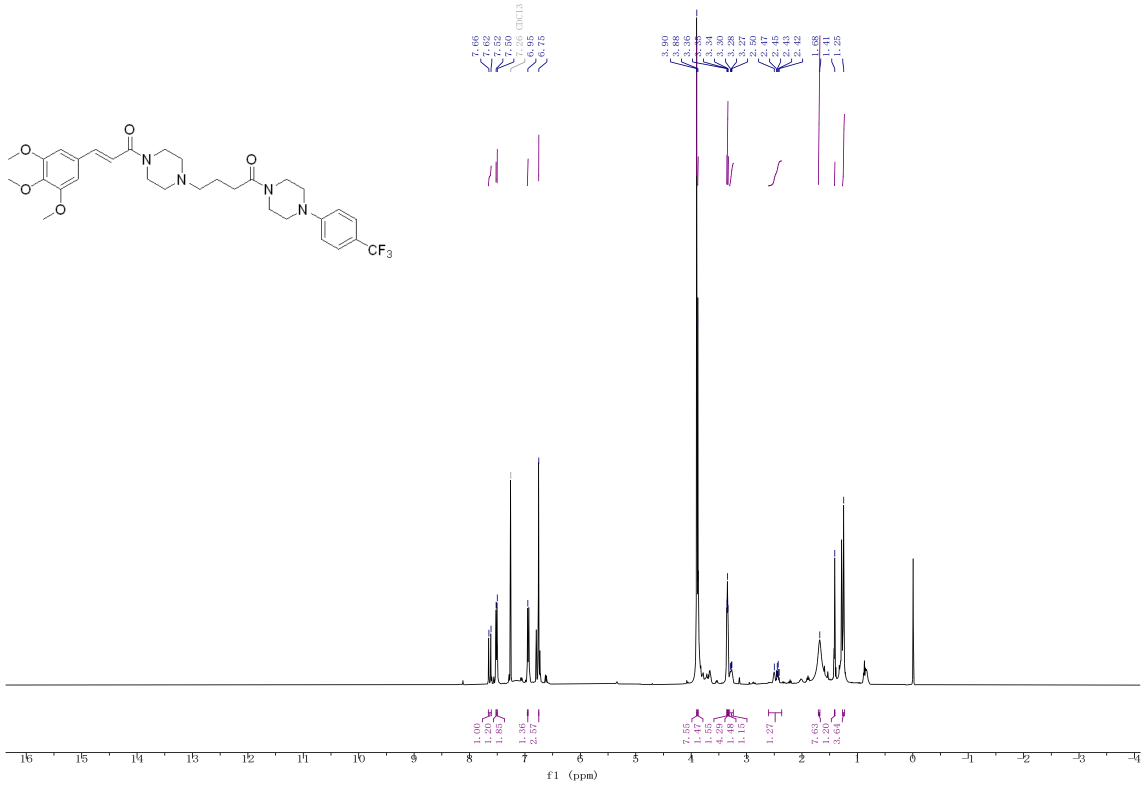

^{1}H NMR (400 MHz, $CDCl_3$) spectrum of **A14**

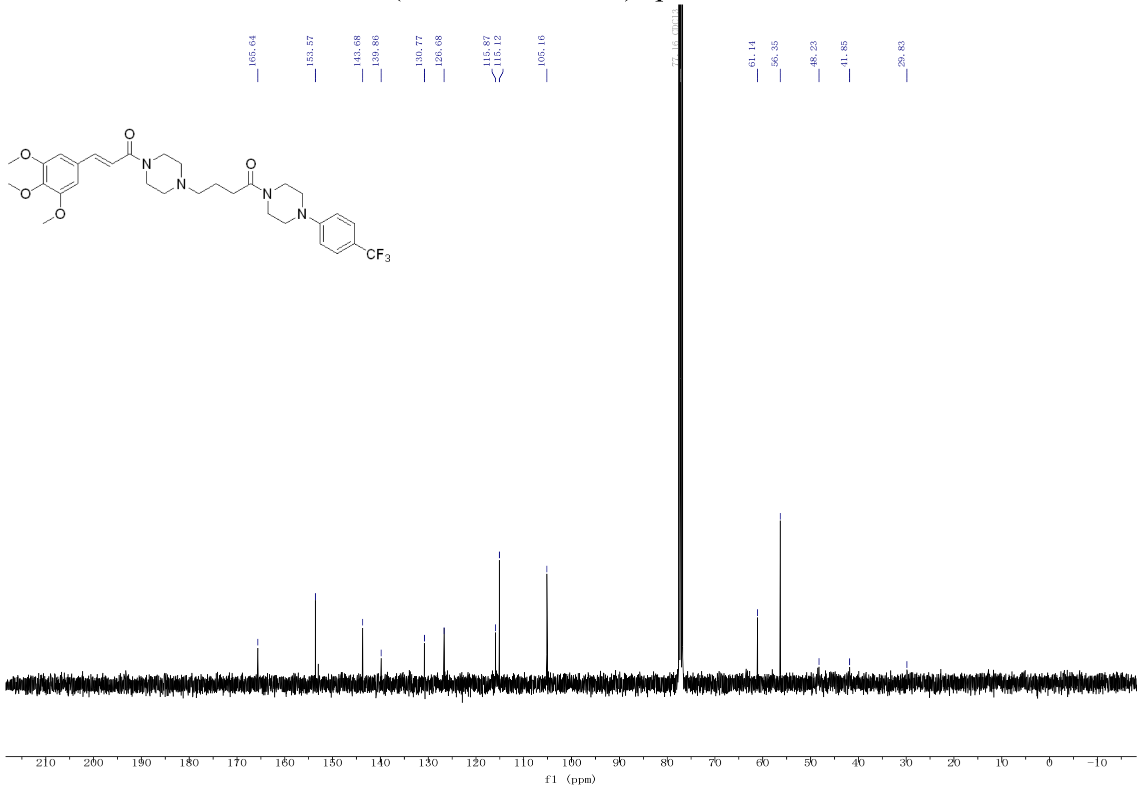

^{13}C NMR (101 MHz, $CDCl_3$) spectrum of **A14**

(E)-1-(4-(4-(trifluoromethyl)phenyl)piperazin-1-yl)-4-(4-(3-(3,4,5-trimethoxyphenyl)acryloyl)piperazin-1-yl)butane-1,4-dione (B14):

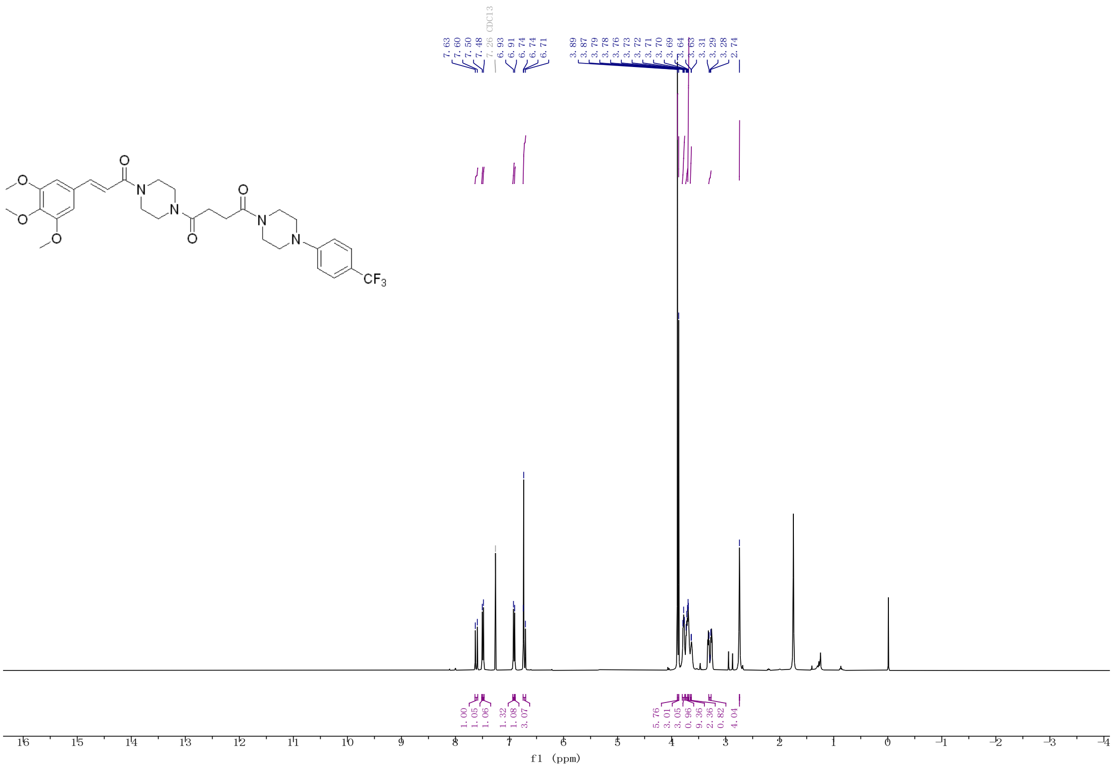

^{1}H NMR (400 MHz, $CDCl_3$) spectrum of **B14**

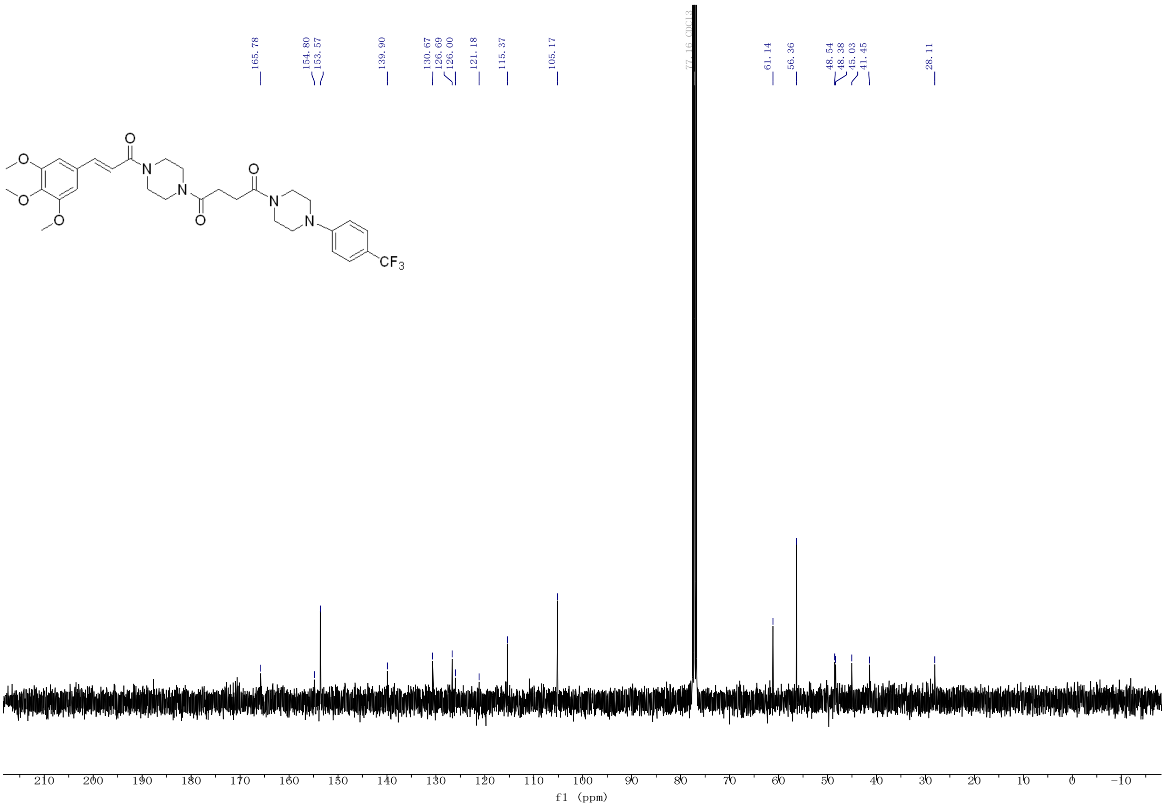

^{13}C NMR (101 MHz, $CDCl_3$) spectrum of **B14**

*(E)-1-(4-(2-methoxyphenyl)piperazin-1-yl)-4-(4-(3-(3,4,5-trimethoxyphenyl)acryloyl)piperazin-1-yl)butan-1-one (**A15**):*

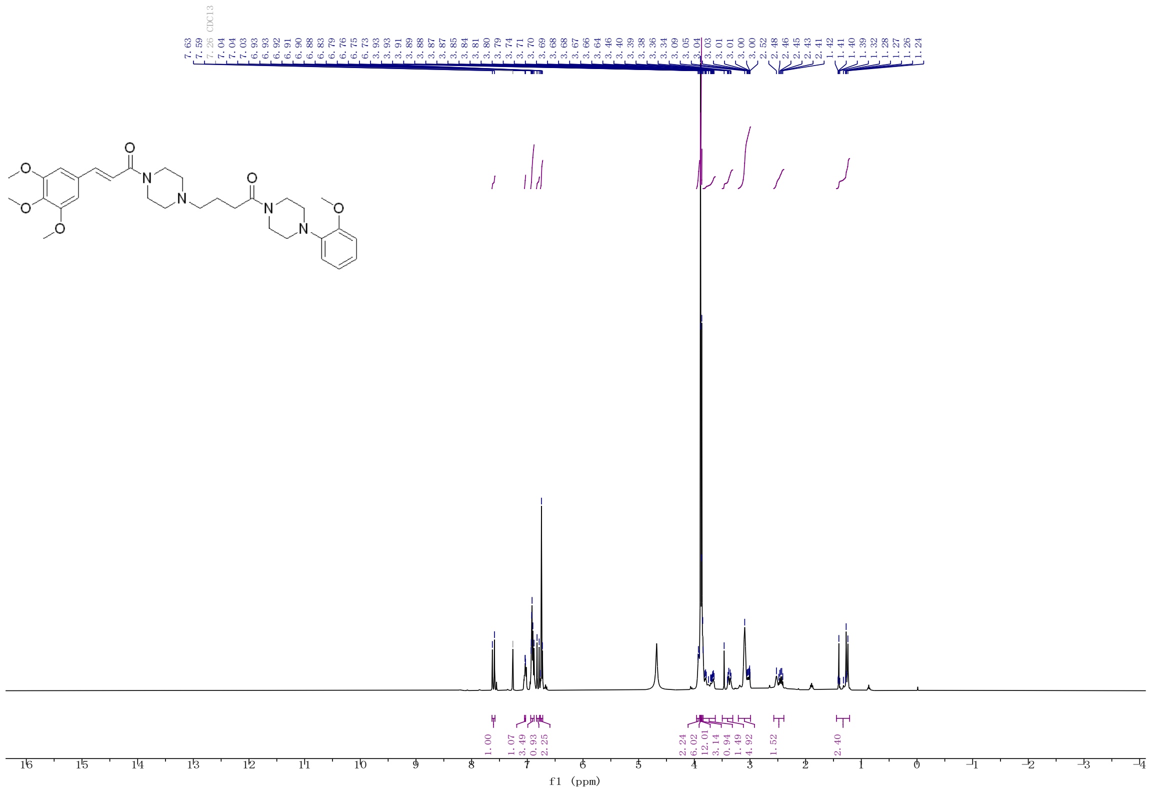

^{1}H NMR (400 MHz, CDCl3) spectrum of **A15**

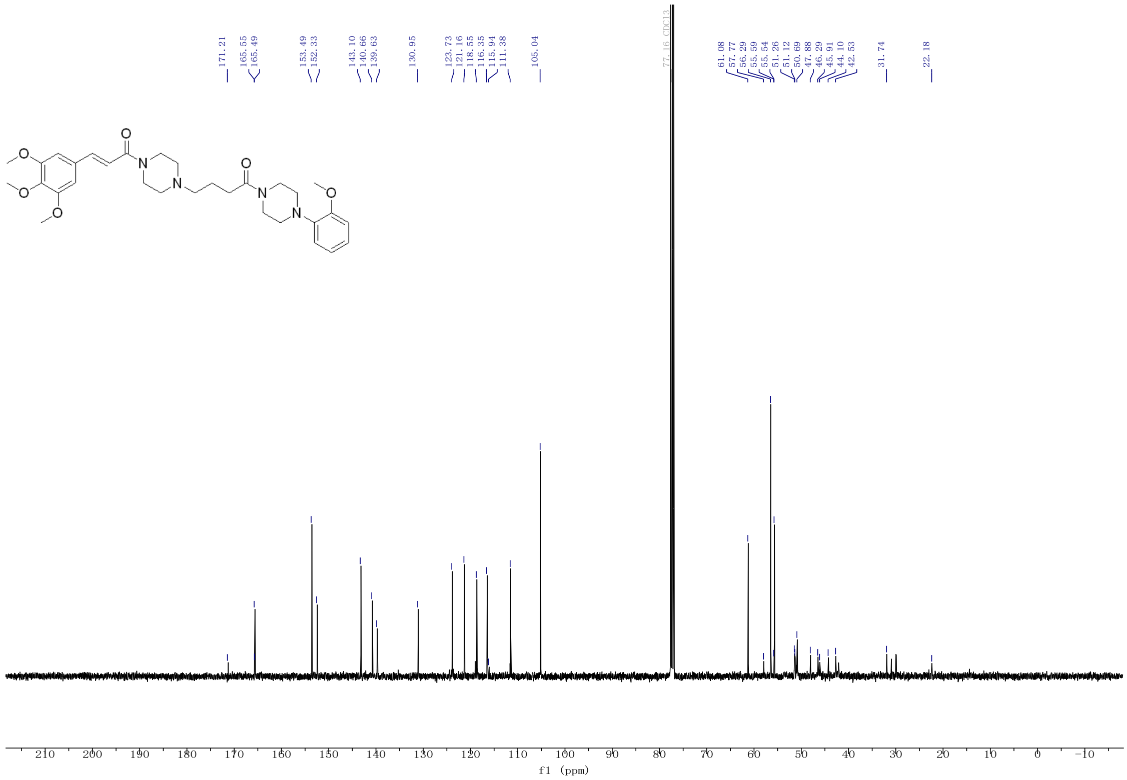

^{13}C NMR (101 MHz, CDCl3) spectrum of **A15**

(E)-1-(4-(2-methoxyphenyl)piperazin-1-yl)-4-(4-(3-(3,4,5-trimethoxyphenyl)acryloyl)piperazin-1-yl)butane-1,4-dione ***(B15)***:

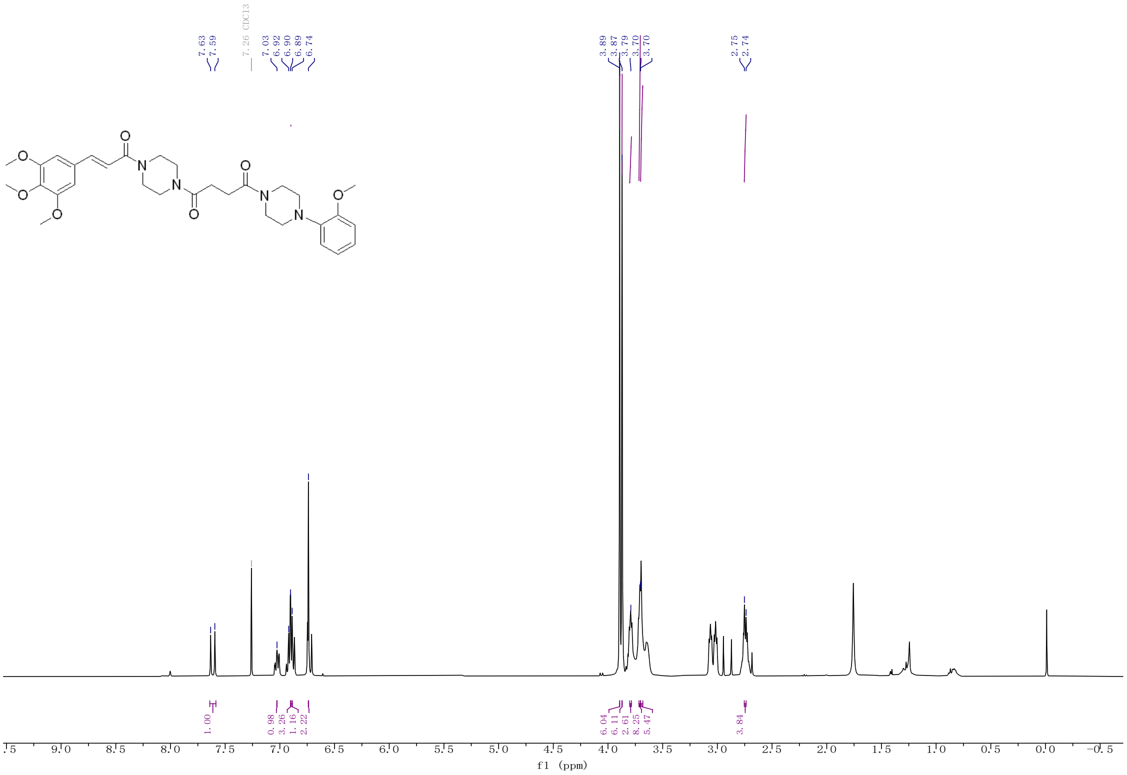

^{1}H NMR (400 MHz, $CDCl_3$) spectrum of **B15**

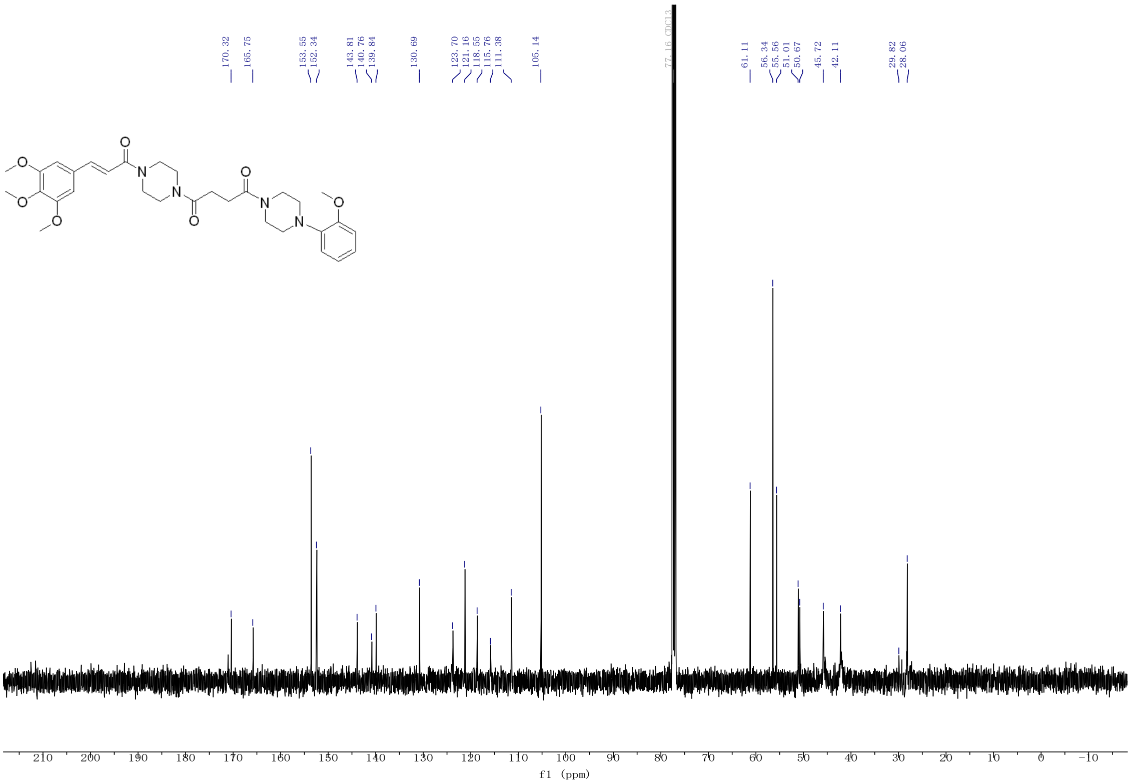

^{13}C NMR (101 MHz, $CDCl_3$) spectrum of **B15**

*(E)-1-(4-(pyrimidin-2-yl)piperazin-1-yl)-4-(4-(3-(3,4,5-trimethoxyphenyl)acryloyl)piperazin-1-yl)butan-1-one (**A16**):*

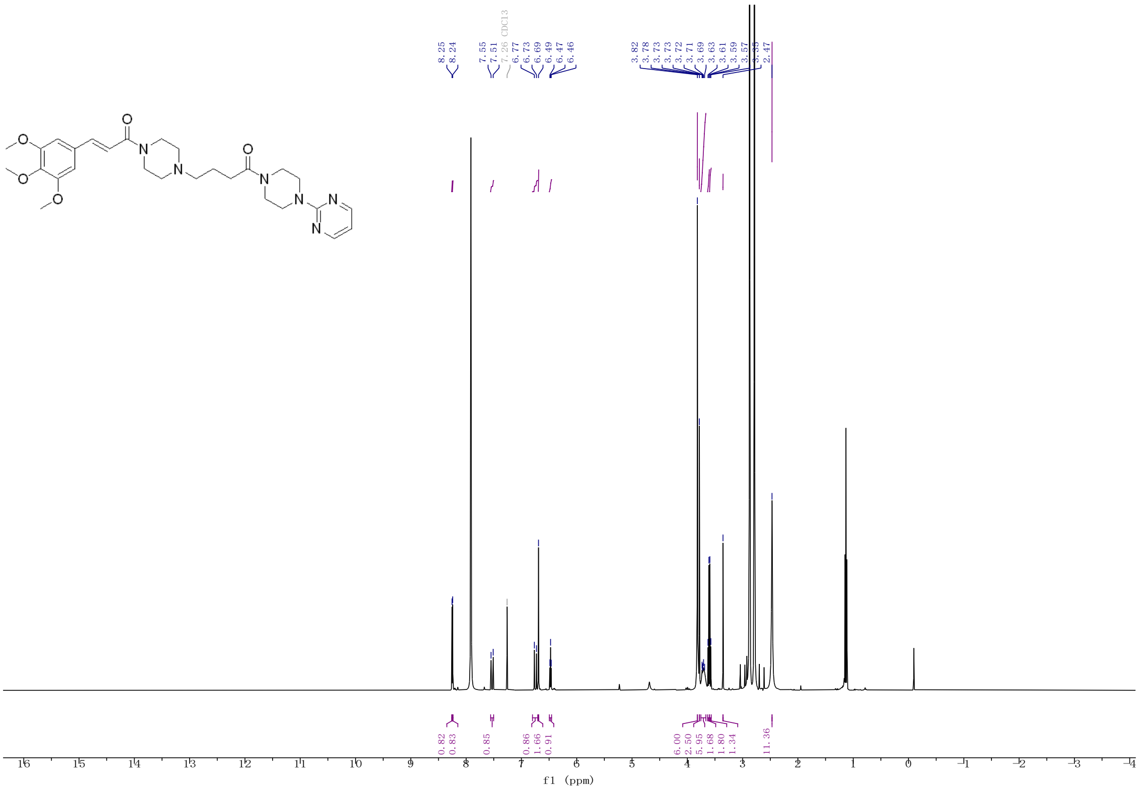

^{1}H NMR (400 MHz, $CDCl_3$) spectrum of **A16**

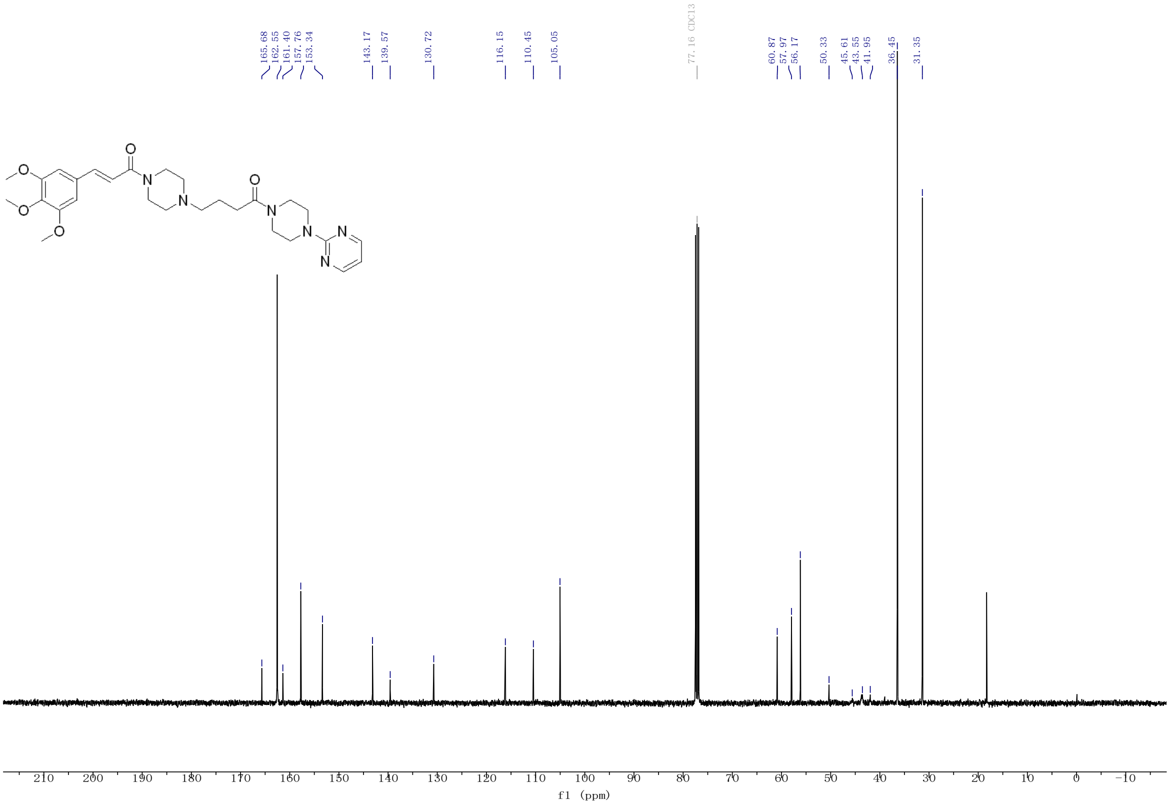

^{13}C NMR (101 MHz, $CDCl_3$) spectrum of **A16**

*(E)-1-(4-(pyrimidin-2-yl)piperazin-1-yl)-4-(4-(3-(3,4,5-trimethoxyphenyl)acryloyl)piperazin-1-yl)butane-1,4-dione (**B16**):*

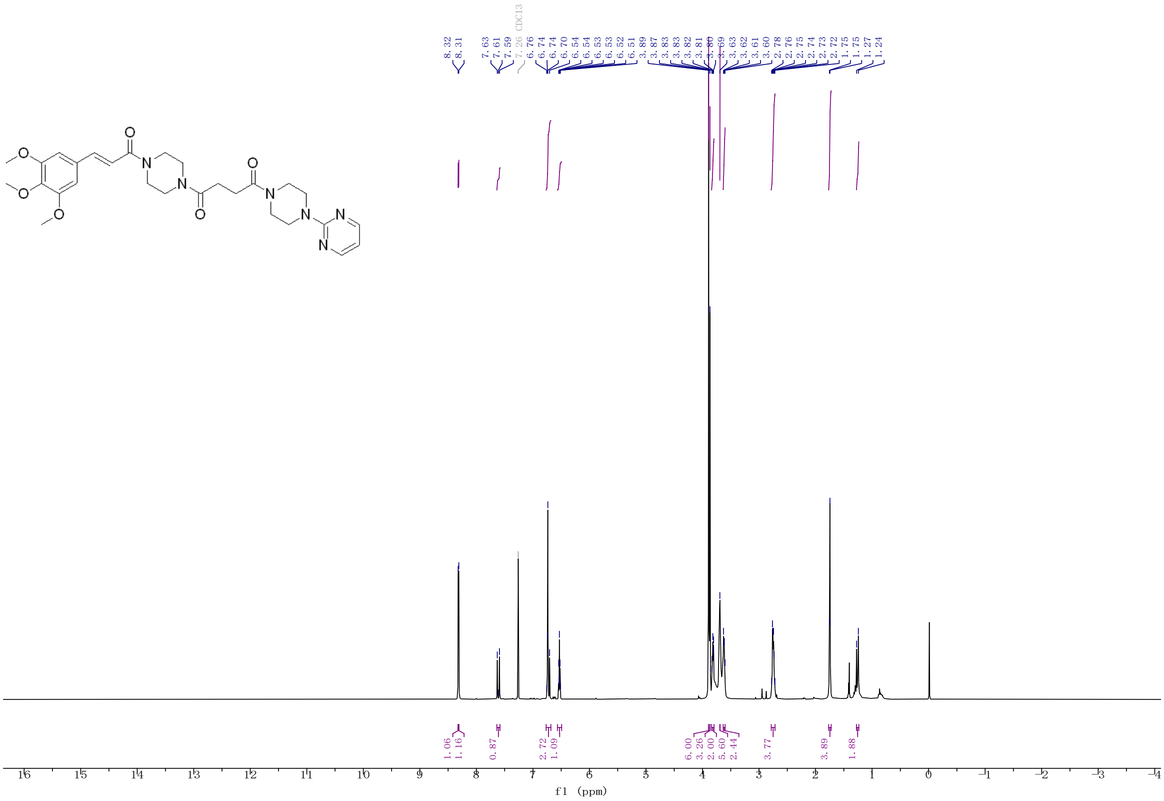

^{1}H NMR (400 MHz, $CDCl_3$) spectrum of **B16**

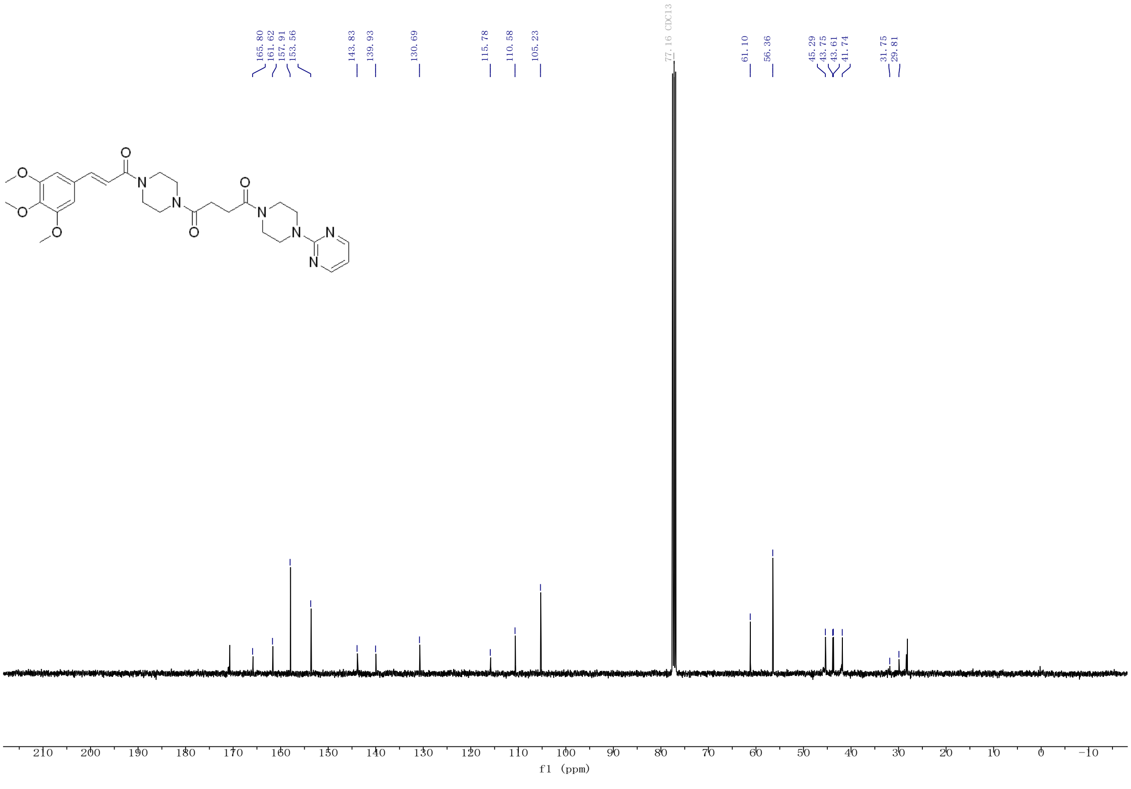

^{13}C NMR (101 MHz, $CDCl_3$) spectrum of **B16**

*(E)-1-(4-(6-fluorobenzo[d]isoxazol-3-yl)piperidin-1-yl)-4-(4-(3-(3,4,5-trimethoxyphenyl)acryloyl)piperazin-1-yl)butan-1-one (**A17**):*

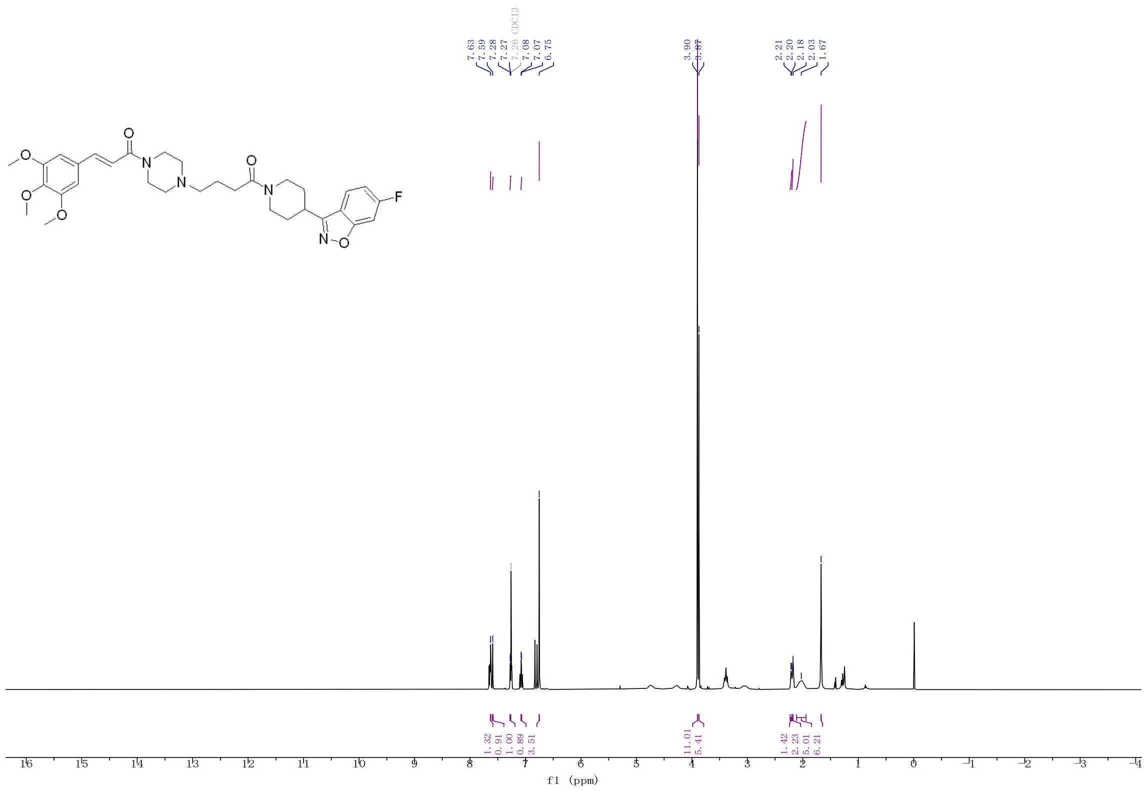

^{1}H NMR (400 MHz, CDCl3) spectrum of **A17**

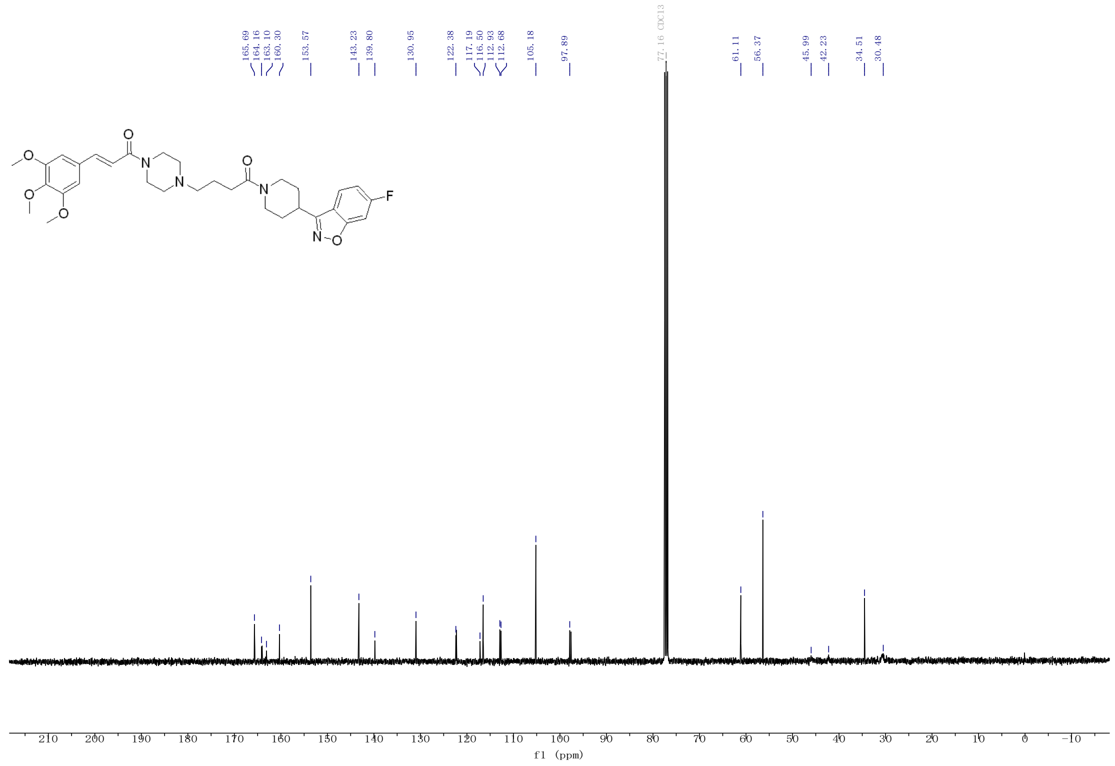

^{13}C NMR (101 MHz, CDCl3) spectrum of **A17**

*(E)-1-(4-(6-fluorobenzo[d]isoxazol-3-yl)piperidin-1-yl)-4-(4-(3-(3,4,5-trimethoxyphenyl)acryloyl)piperazin-1-yl)butane-1,4-dione (**B17**):*

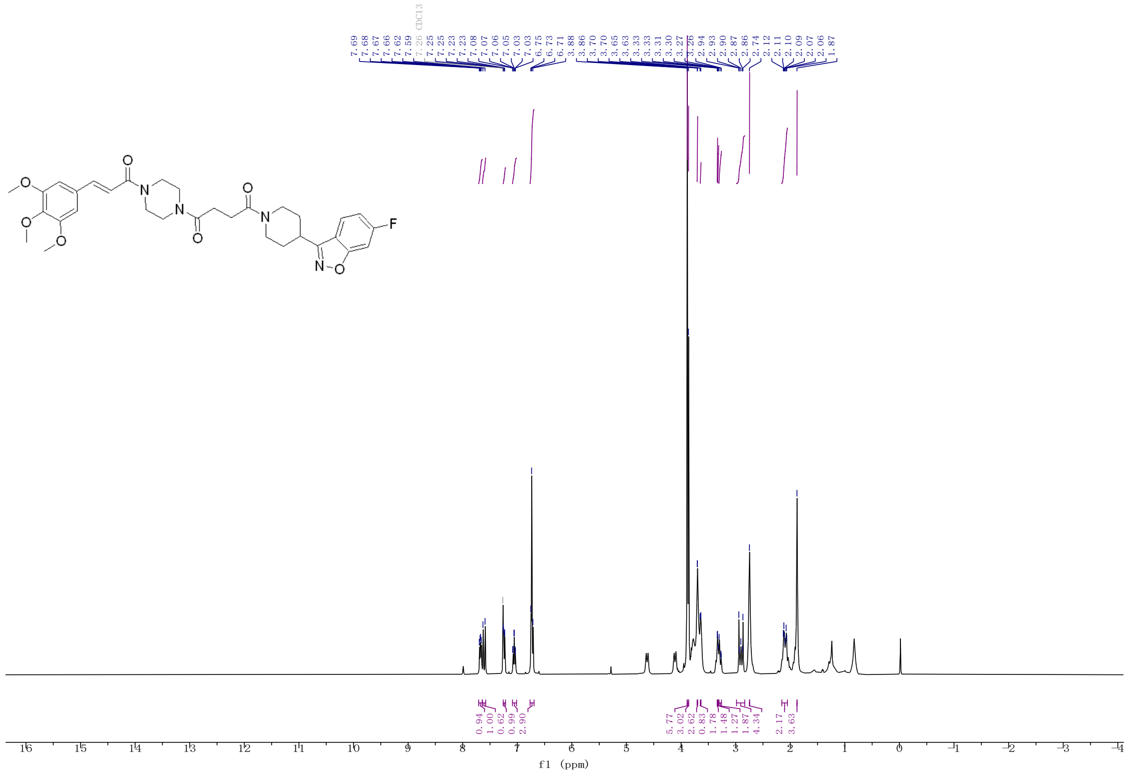

^{1}H NMR (400 MHz, CDCl$_3$) spectrum of **B17**

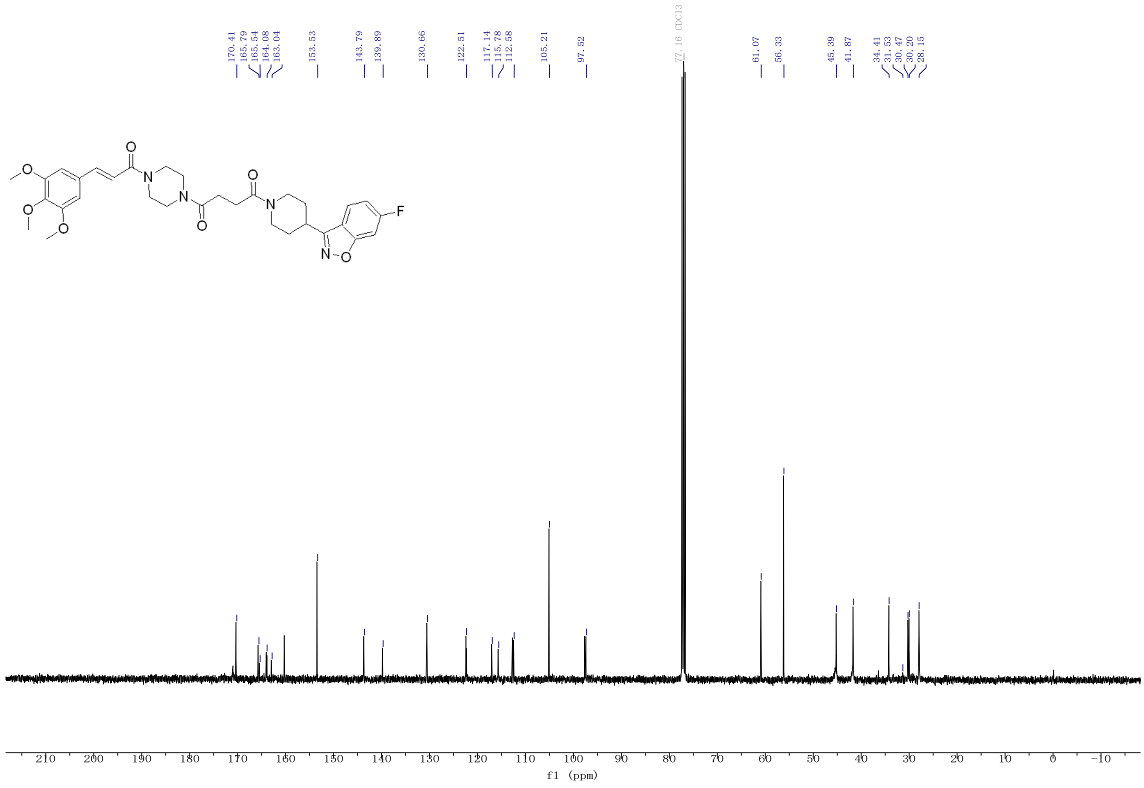

^{13}C NMR (101 MHz, CDCl$_3$) spectrum of **B17**

LC-MS spectrum of synthesized derivatives

Spectrum from DataSET13.wiff (sample 42) - 230505 synthetic 017, +TOF MS (100 - 1300) from 0.074 min

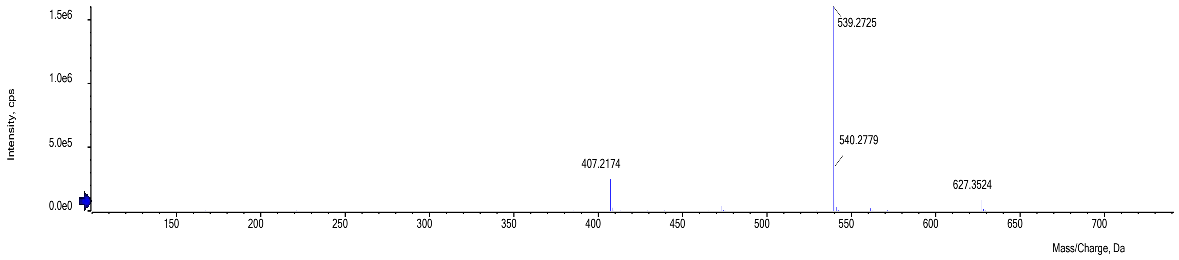

LC-MS spectrum of **A2**

Spectrum from DataSET14.wiff (sample 38) - 230529 syn030, +TOF MS (100 - 1300) from 0.107 min

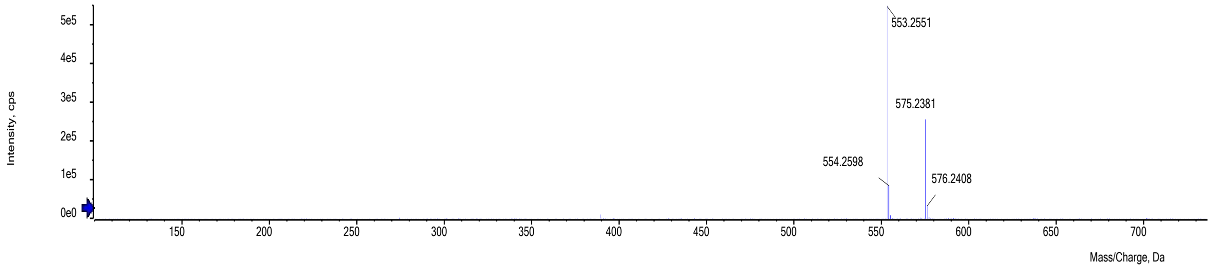

LC-MS spectrum of **B2**

Spectrum from DataSET14.wiff (sample 24) - 230529 syn016, +TOF MS (100 - 1300) from 0.051 min

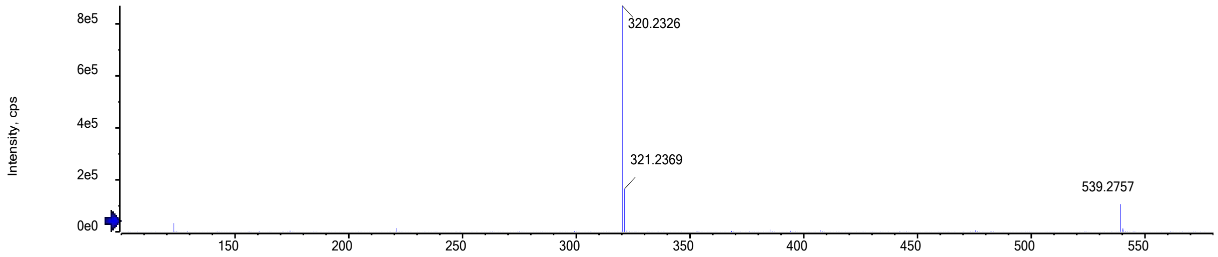

LC-MS spectrum of **A3**

Spectrum from DataSET14.wiff (sample 28) - 230529 syn020, +TOF MS (100 - 1300) from 0.088 min

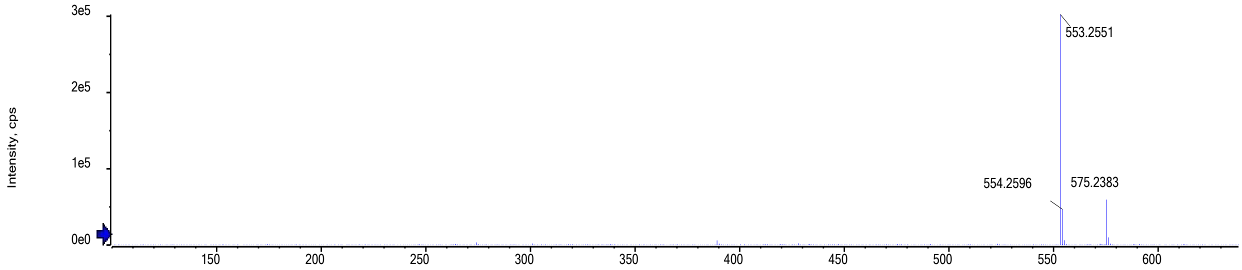

LC-MS spectrum of **B3**

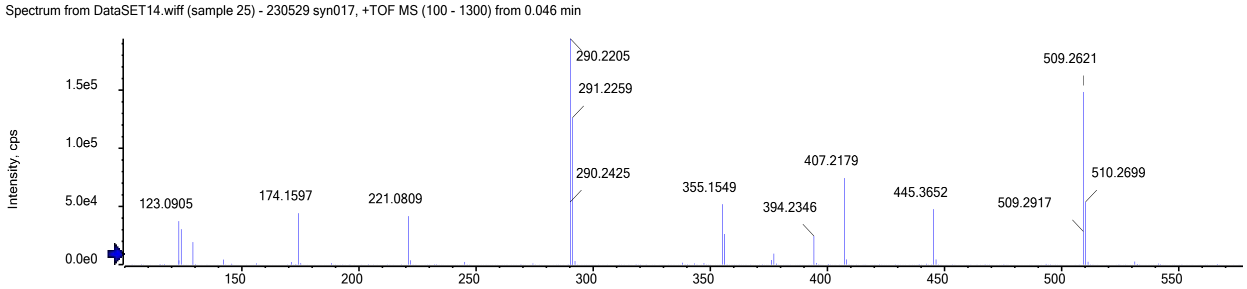

LC-MS spectrum of **A4**

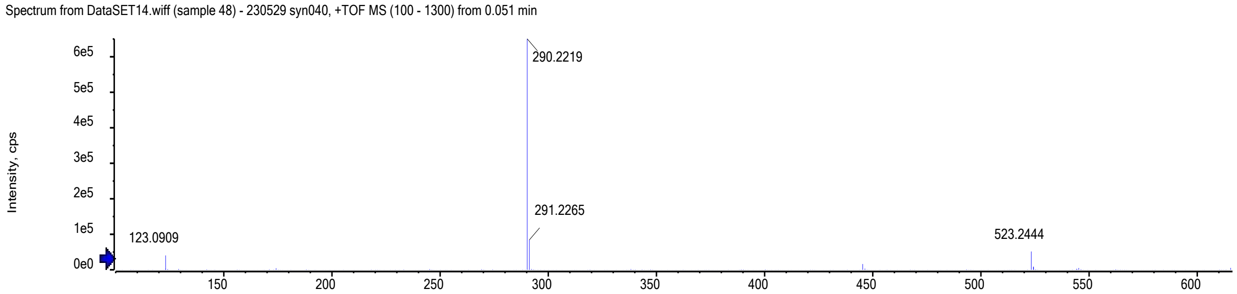

LC-MS spectrum of **B4**

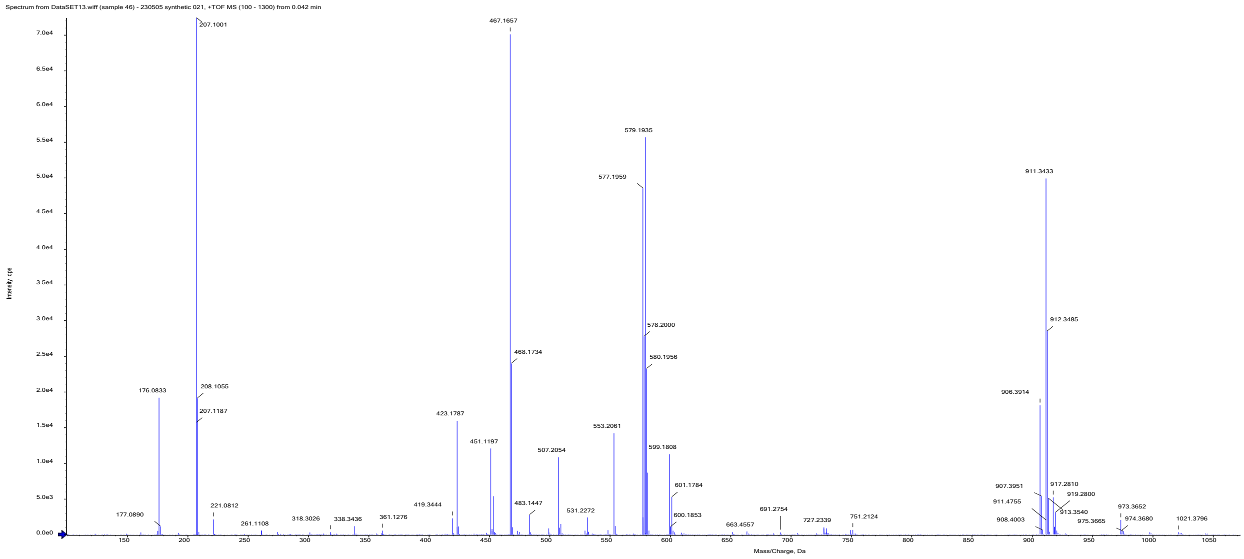

LC-MS spectrum of **A5**

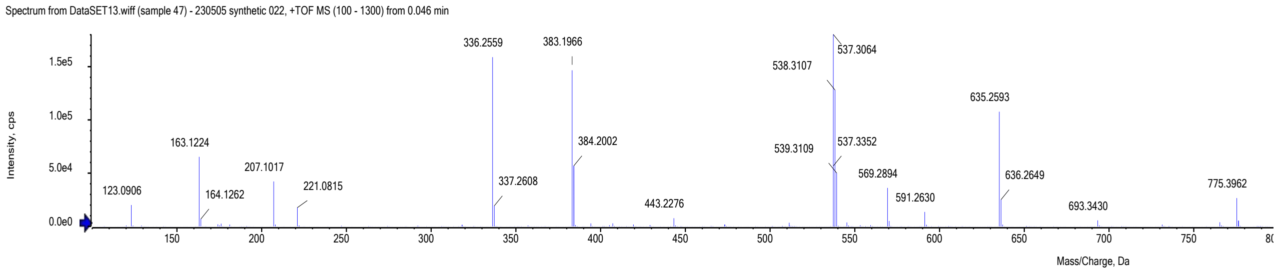

LC-MS spectrum of **B5**

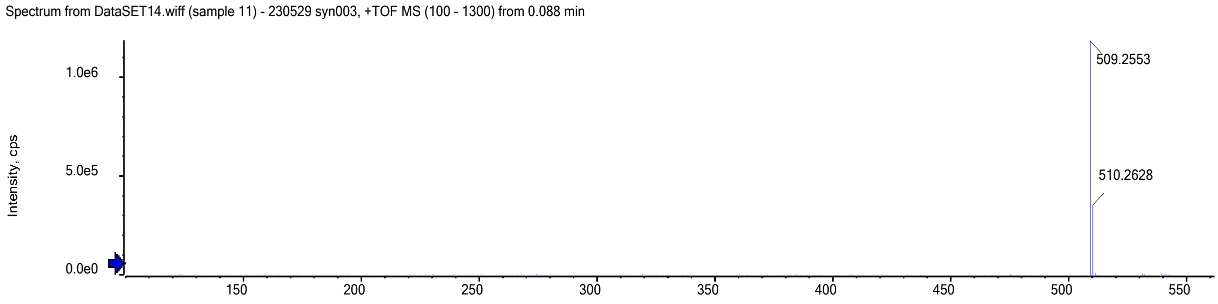

LC-MS spectrum of **A6**

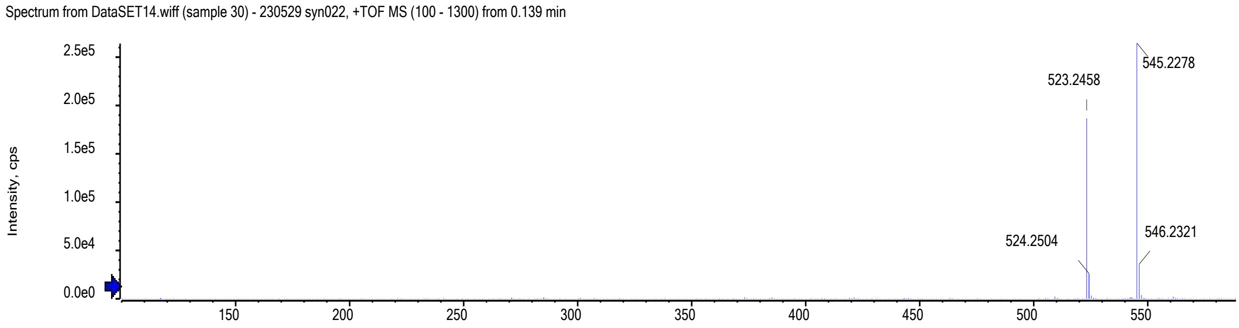

LC-MS spectrum of **B6**

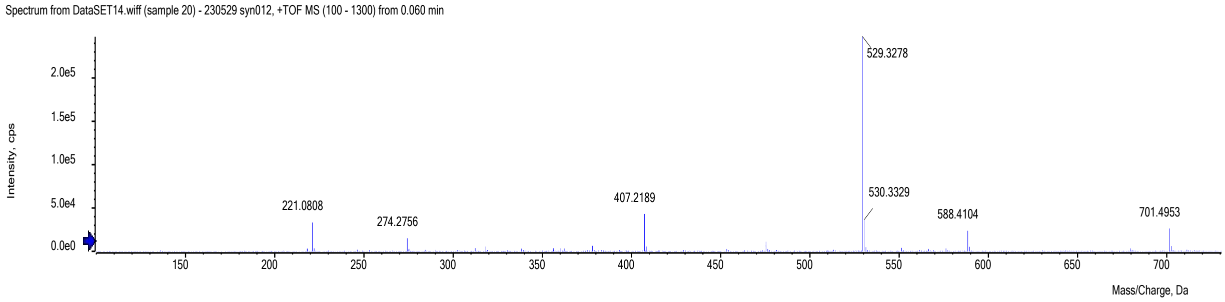

LC-MS spectrum of **A7**

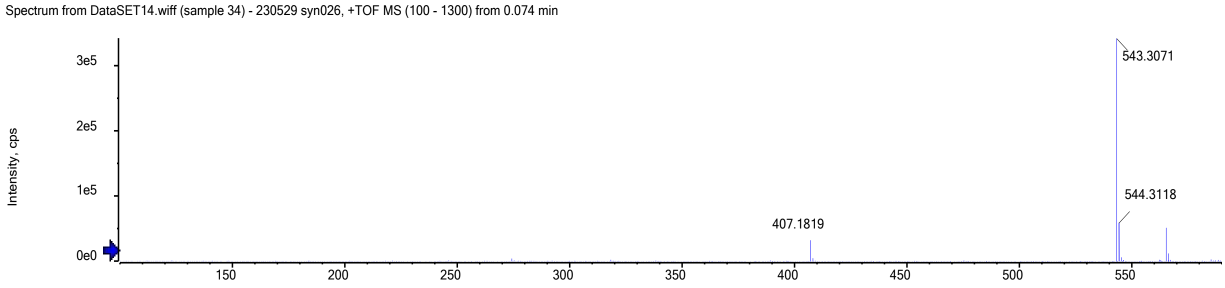

LC-MS spectrum of **B7**

Spectrum from DataSET13.wiff (sample 27) - 230505 synthetic 002, +TOF MS (100 - 1300) from 0.070 min

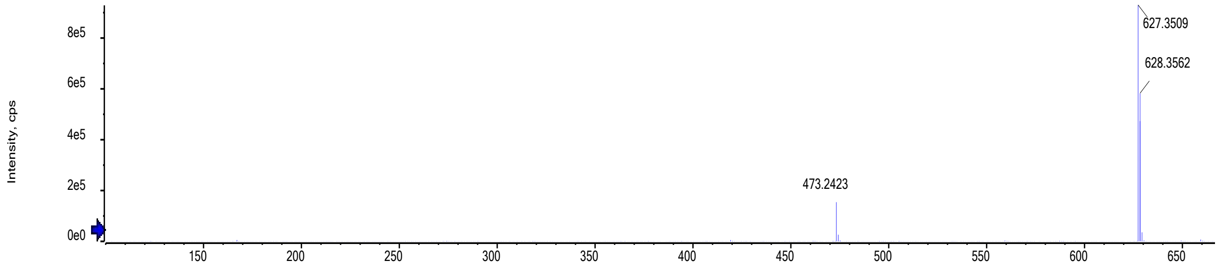

LC-MS spectrum of **A8**

Spectrum from DataSET14.wiff (sample 37) - 230529 syn029, +TOF MS (100 - 1300) from 0.139 min

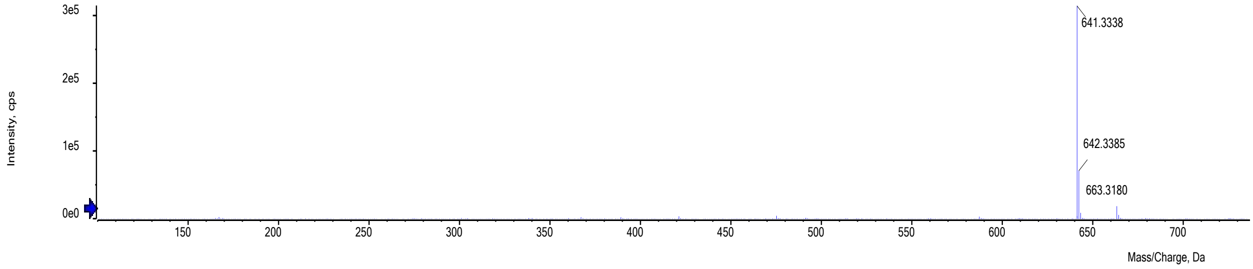

LC-MS spectrum of **B8**

Spectrum from DataSET13.wiff (sample 58) - Sam0609004, +TOF MS (100 - 1300) from 0.042 min

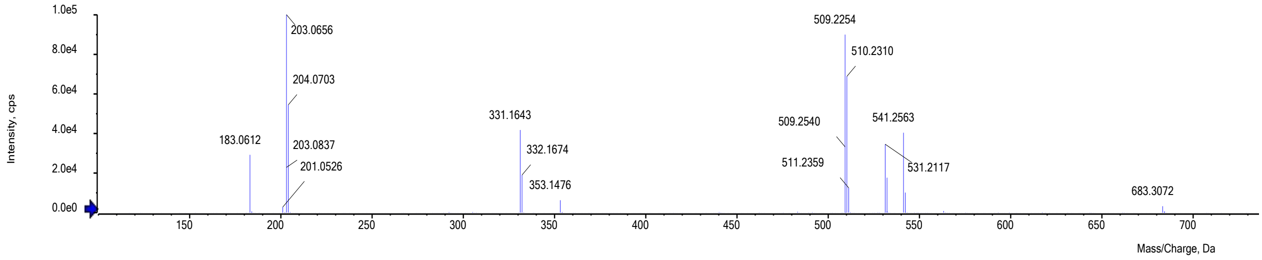

LC-MS spectrum of **A9**

Spectrum from DataSET13.wiff (sample 66) - Sam0609012, +TOF MS (100 - 1300) from 0.116 min

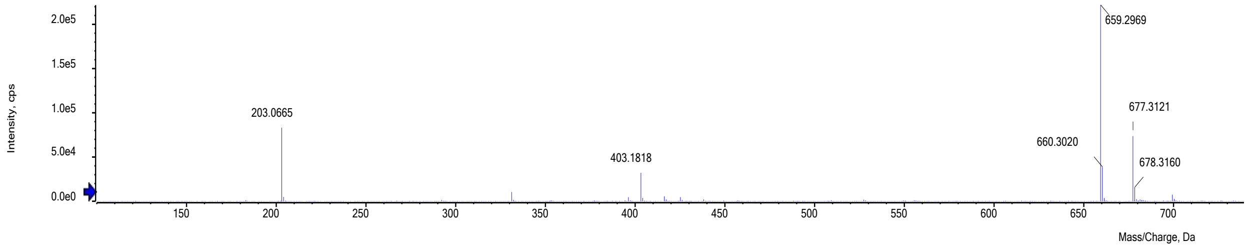

LC-MS spectrum of **B9**

Spectrum from DataSET13.wiff (sample 55) - Sam0609001, +TOF MS (100 - 1300) from 0.046 min

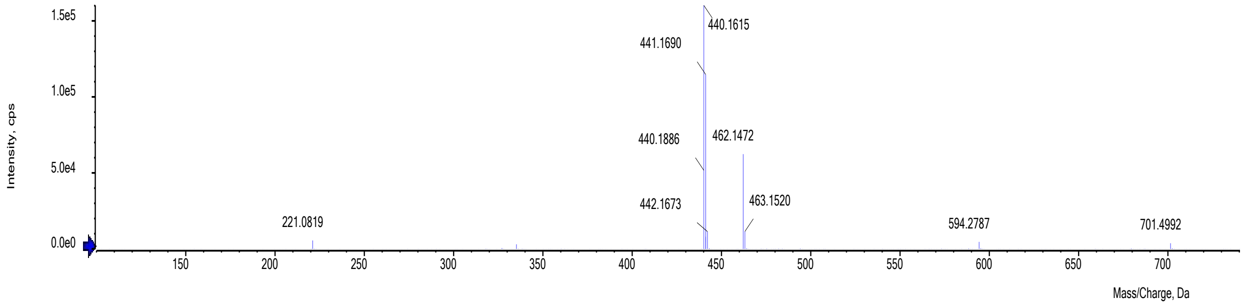

LC-MS spectrum of **A10**

Spectrum from DataSET14.wiff (sample 43) - 230529 syn035, +TOF MS (100 - 1300) from 0.065 min

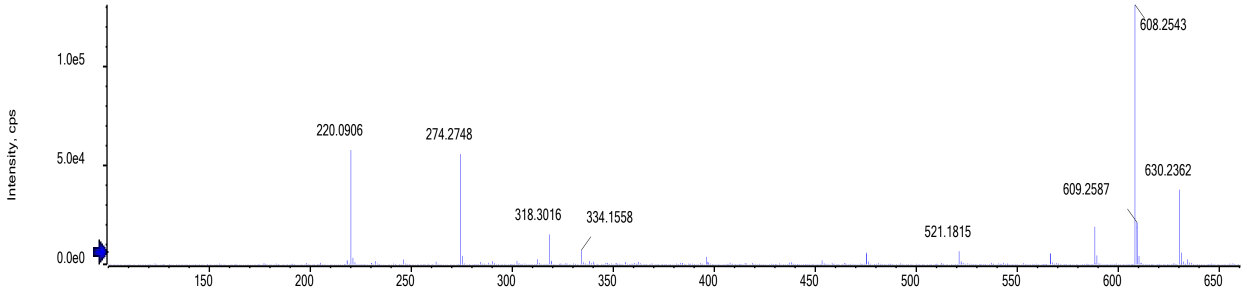

LC-MS spectrum of **B10**

Spectrum from DataSET13.wiff (sample 31) - 230505 synthetic 006, +TOF MS (100 - 1300) from 0.051 min

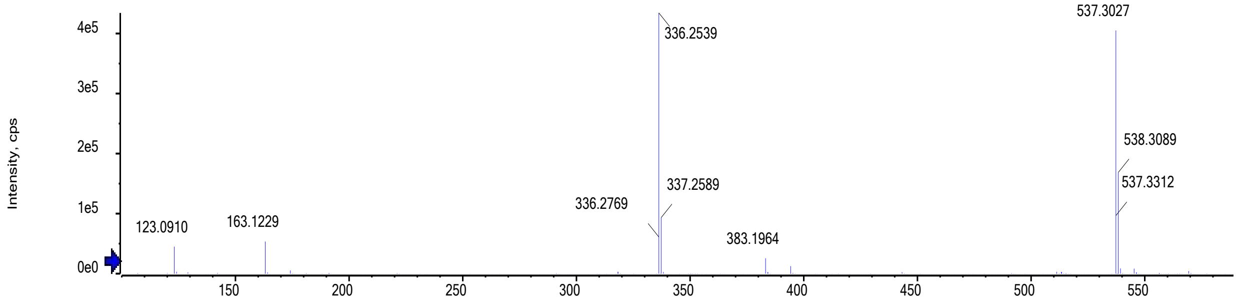

LC-MS spectrum of **A11**

Spectrum from DataSET14.wiff (sample 36) - 230529 syn028, +TOF MS (100 - 1300) from 0.107 min

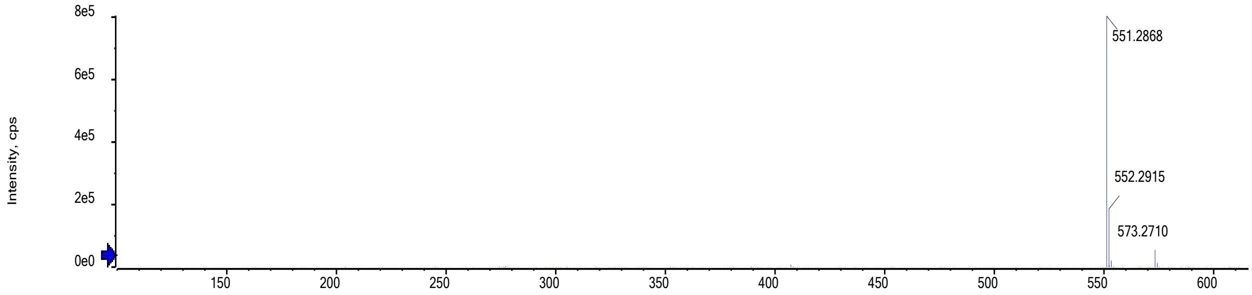

LC-MS spectrum of **B11**

Spectrum from DataSET13.wiff (sample 29) - 230505 synthetic 004, +TOF MS (100 - 1300) from 0.046 min

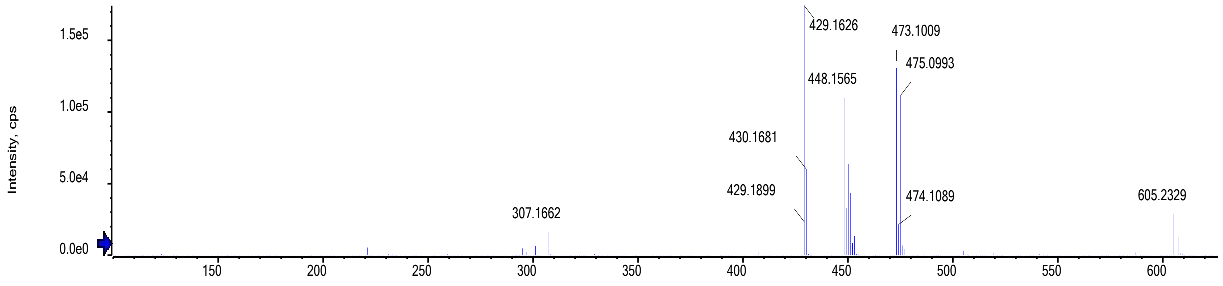

LC-MS spectrum of **A12**

Spectrum from DataSET14.wiff (sample 31) - 230529 syn023, +TOF MS (100 - 1300) from 0.070 min

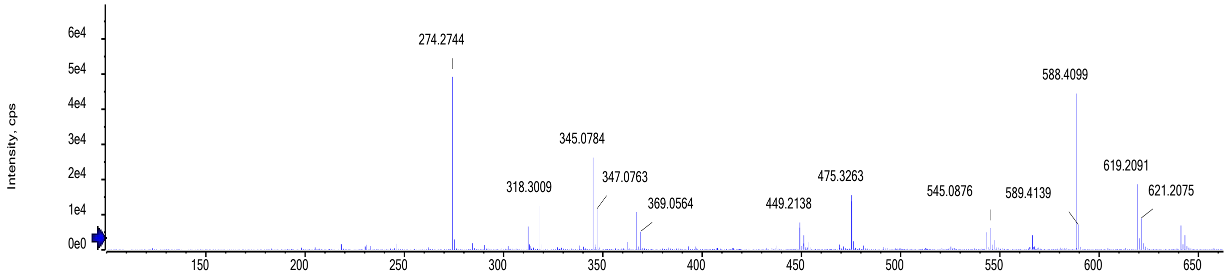

LC-MS spectrum of **B12**

Spectrum from DataSET13.wiff (sample 32) - 230505 synthetic 007, +TOF MS (100 - 1300) from 0.056 min

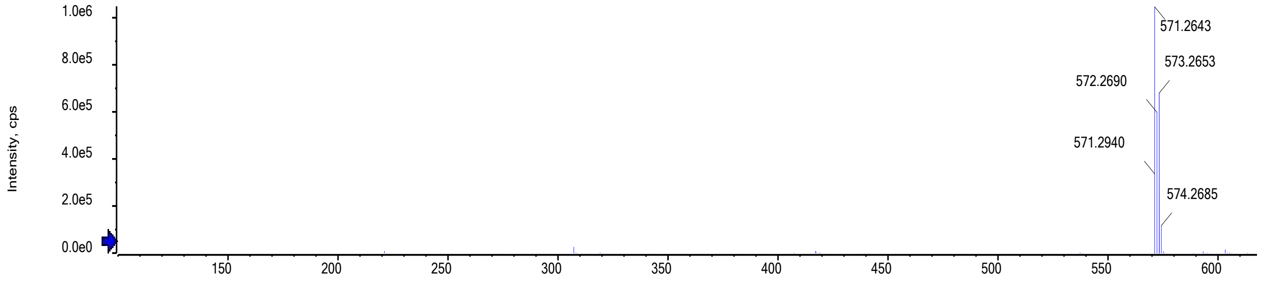

LC-MS spectrum of **A13**

Spectrum from DataSET14.wiff (sample 39) - 230529 syn031, +TOF MS (100 - 1300) from 0.056 min

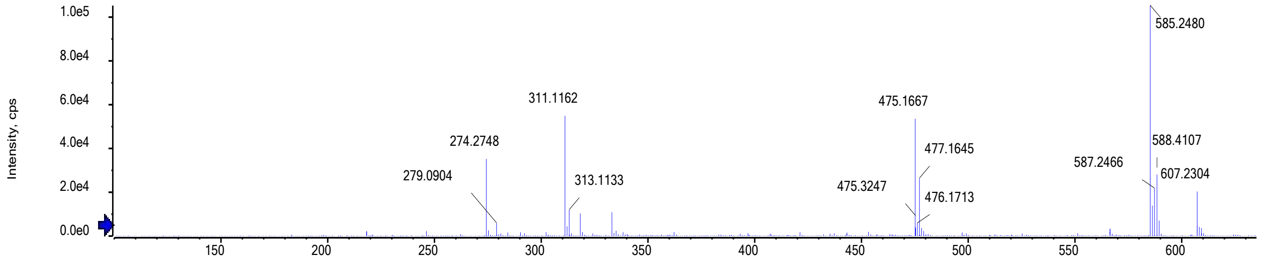

LC-MS spectrum of **B13**

Spectrum from DataSET13.wiff (sample 38) - 230505 synthetic 013, +TOF MS (100 - 1300) from 0.088 min

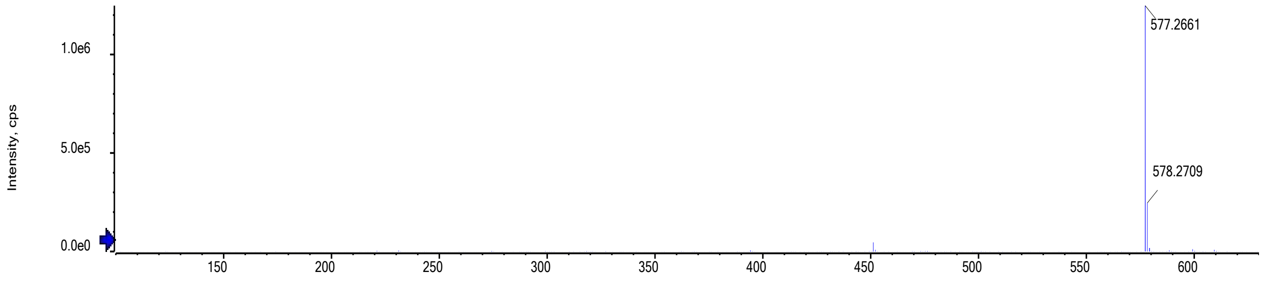

LC-MS spectrum of **A14**

Spectrum from DataSET14.wiff (sample 32) - 230529 syn024, +TOF MS (100 - 1300) from 0.051 min

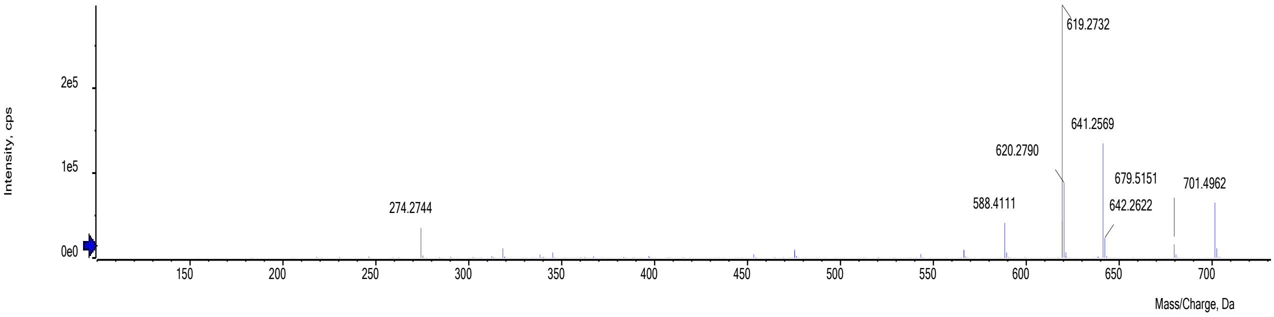

LC-MS spectrum of **B14**

Spectrum from DataSET13.wiff (sample 33) - 230505 synthetic 008, +TOF MS (100 - 1300) from 0.121 min

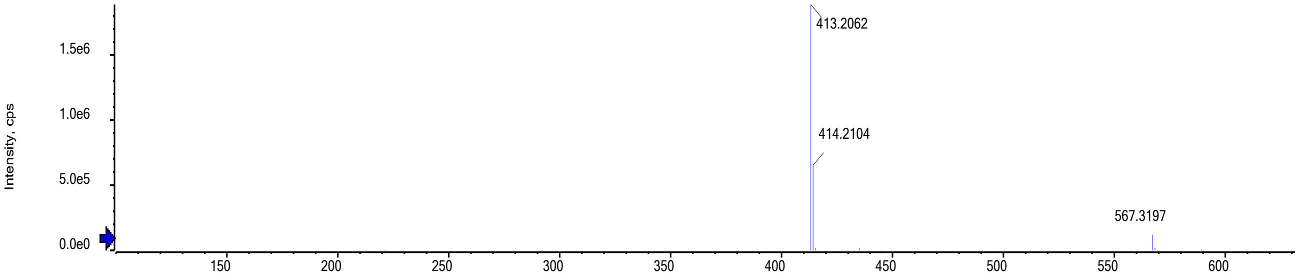

LC-MS spectrum of **A15**

Spectrum from DataSET14.wiff (sample 40) - 230529 syn032, +TOF MS (100 - 1300) from 0.065 min

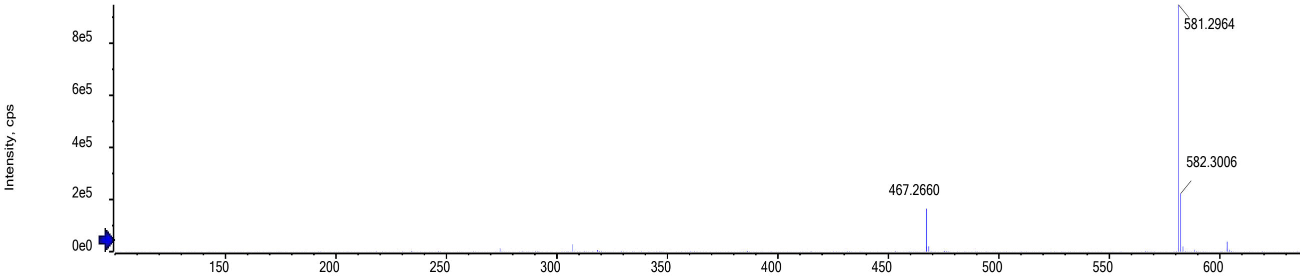

LC-MS spectrum of **B15**

Spectrum from DataSET14.wiff (sample 12) - 230529 syn004, +TOF MS (100 - 1300) from 0.051 min

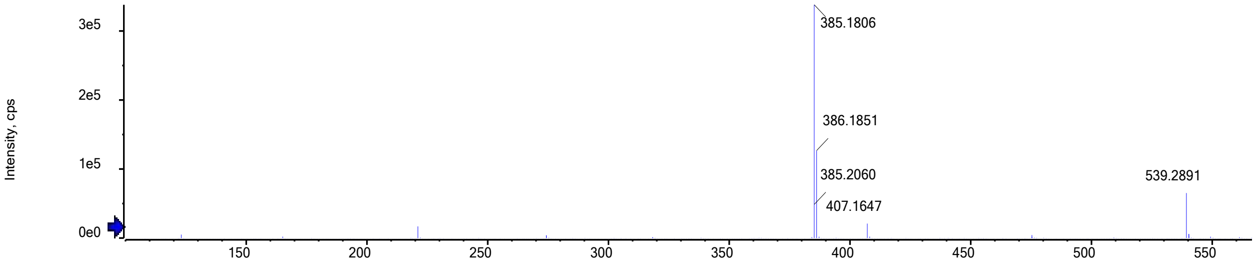

LC-MS spectrum of **A16**

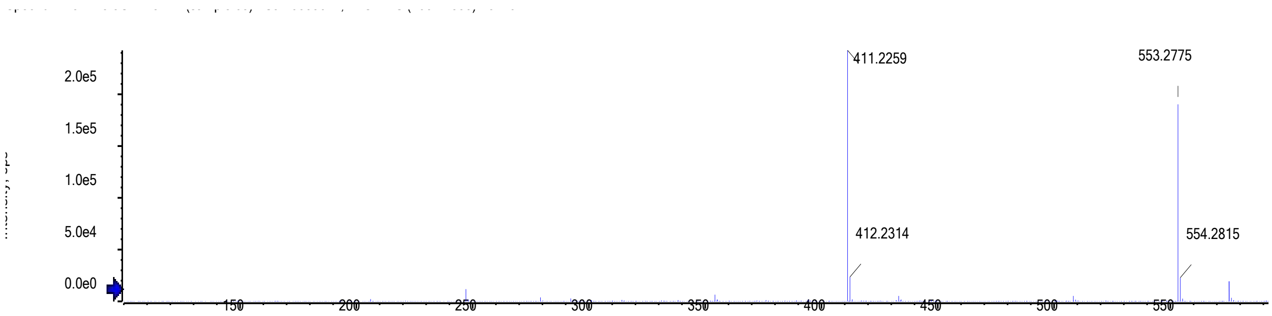

LC-MS spectrum of **B16**

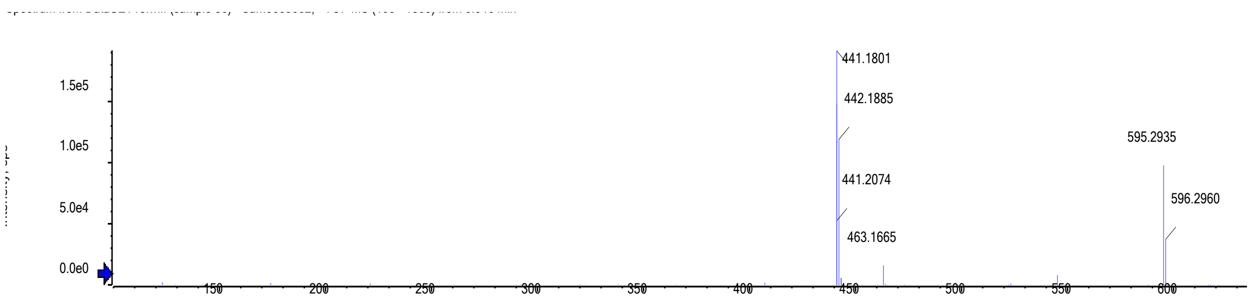

LC-MS spectrum of **A17**

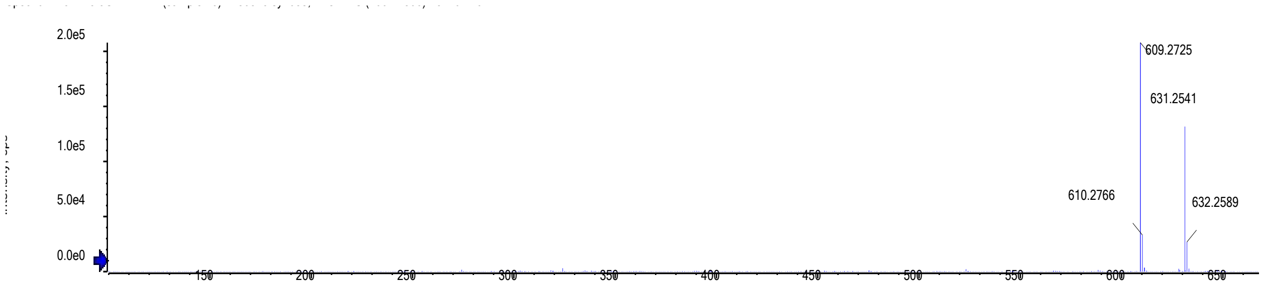

LC-MS spectrum of **B17**

Table S1. ADMET properties of compounds **A14** and STP.

Category	Property	ADMET lab 3	VNN-ADMET	PreADMET	PkCSM	Consensus
**A14**						
**Absorption**	Water solubility	-	-	14.512 mg/L	-5.446 log mol/L	Low
	Caco-2 permeability	-4.898 log unit	-	51.220 nm/sec	1.199 log Papp in 10^{-6} cm/s	Moderate
	Human intestinal absorption (%)	-	-	97.562	93.621	High
	Skin permeability (log Kp)	-	-	-2.215	-2.756	Low
	P-gp inhibitor	Yes	No	Yes	Yes	Yes
**Distribution**	VDss (human, log L/kg)	-	-	-	0.729	Low
	BBB permeability (log BB)	No	Yes	Yes	-1.435	Yes
**Metabolism**	CYP1A2 inhibitor	No	No	-	No	No
	CYP2C9 inhibitor	No	No	No	No	No
	CYP2C19 inhibitor	No	No	No	No	No
	CYP2D6 inhibitor	No	No	No	No	No
	CYP3A4 inhibitor	Yes	No	Yes	Yes	Yes
**Excretion**	Total clearance (log ml/min/kg)	-	-	-	0.509	Low
**Toxicity**	AMES toxicity	No	No	No	No	No
	hERG blocker	Yes	Yes	Medium risk	No	Yes
**STP**						
**Absorption**	Water solubility	-	-	373.226 mg/L	-3.098 log mol/L	Moderate
	Caco-2 permeability	-4.492 log unit	-	44.979 nm/sec	1.945 log Papp in 10^{-6} cm/s	Moderate
	Human intestinal absorption (%)	-	-	93.304	93.322	High
	Skin permeability (log Kp)	-	-	-2.161	-2.444	Low
	P-gp inhibitor	Yes	Yes	No	Yes	Yes
**Distribution**	VDss (human, log L/kg)	-	-	-	0.371	Low
	BBB permeability (log BB)	No	No	Yes	0.079	Yes
**Metabolism**	CYP1A2 inhibitor	Yes	No	-	Yes	Yes
	CYP2C9 inhibitor	No	No	Yes	No	No
	CYP2C19 inhibitor	Yes	No	Yes	Yes	Yes
	CYP2D6 inhibitor	Yes	No	No	No	No
	CYP3A4 inhibitor	Yes	No	Yes	No	Yes
**Excretion**	Total clearance (log ml/min/kg)	-	-	-	-0.02	Low
**Toxicity**	AMES toxicity	No	No	No	No	No
	hERG blocker	No	No	No	No	No

Docking results of original ligand GEN-140 interacting with LDH (PDB:4ZVV).

Original co-crystallised interaction

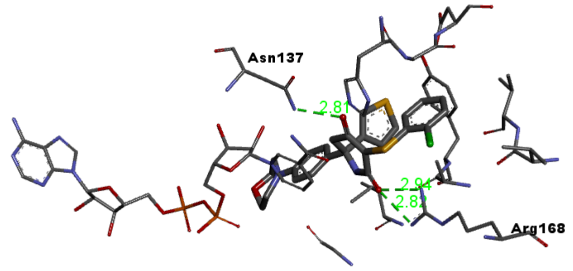

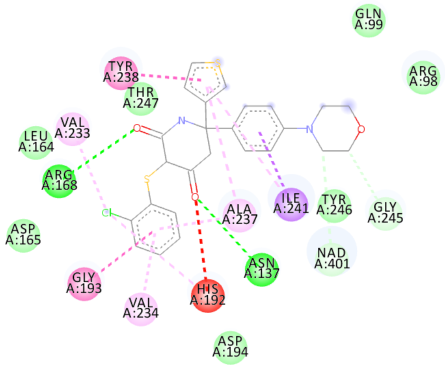

Data result

mode	affinity (kcal/mol)	dist from best mode rmsd l.b.	rmsd u.b.
1	-7.8	0.000	0.000
2	-7.8	1.105	1.518
3	-7.2	4.455	8.159
4	-7.2	5.898	7.647
5	-7.1	4.473	6.889
6	-7.1	5.019	7.268
7	-7.1	2.652	3.234
8	-7.0	4.453	8.035
9	-7.0	3.073	4.708
10	-6.9	1.748	3.177

Typical interaction

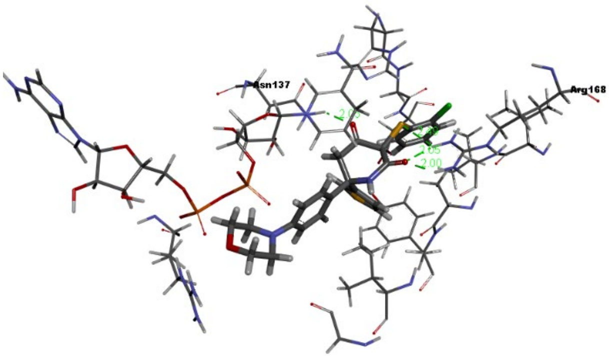

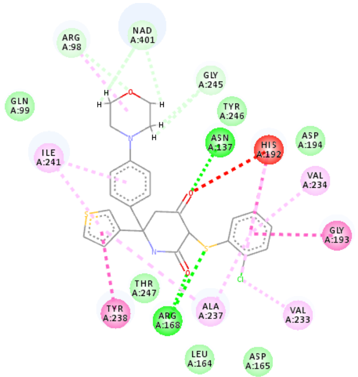

Conformational superposition results and RMSD values

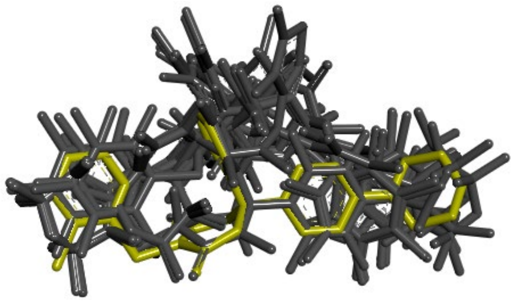

Heavy Atom RMSD to Ori 11

Name	Reference	RMSD (A)
1 1	Ori 11	0.8281
2 2	Ori 11	9.1980
3 3	Ori 11	9.1716
4 4	Ori 11	8.6824
5 5	Ori 11	9.0707
6 6	Ori 11	9.4101
7 7	Ori 11	8.7080
8 8	Ori 11	3.0607
9 9	Ori 11	3.9411
10 10	Ori 11	9.0680
